# Multi‐omics analysis identifies a *CYP9K1* haplotype conferring pyrethroid resistance in the malaria vector *Anopheles funestus* in East Africa

**DOI:** 10.1111/mec.16497

**Published:** 2022-05-24

**Authors:** Jack Hearn, Carlos S. Djoko Tagne, Sulaiman S. Ibrahim, Billy Tene‐Fossog, Leon M. J. Mugenzi, Helen Irving, Jacob M. Riveron, Gareth D. Weedall, Charles S. Wondji

**Affiliations:** ^1^ 9655 Vector Biology Department Liverpool School of Tropical Medicine Liverpool UK; ^2^ LSTM Research Unit Centre for Research in Infectious Diseases (CRID) Yaoundé Cameroon; ^3^ 422598 Department of Biochemistry Faculty of Science University of Bamenda Bamenda Cameroon; ^4^ 4589 School of Biological and Environmental Sciences Liverpool John Moores University Liverpool UK

**Keywords:** multi‐omics, pyrethroid

## Abstract

Metabolic resistance to pyrethroids is a menace to the continued effectiveness of malaria vector controls. Its molecular basis is complex and varies geographically across Africa. Here, we used a multi‐omics approach, followed‐up with functional validation to show that a directionally selected haplotype of a cytochrome P450, *CYP9K1* is a major driver of resistance in *Anopheles funestus*. A PoolSeq GWAS using mosquitoes alive and dead after permethrin exposure, from Malawi and Cameroon, detected candidate genomic regions, but lacked consistency across replicates. Targeted sequencing of candidate resistance genes detected several SNPs associated with known pyrethroid resistance QTLs. The most significant SNPs were in the cytochrome P450 *CYP304B1* (Cameroon), *CYP315A1* (Uganda) and the ABC transporter gene ABCG4 (Malawi). However, when comparing field resistant mosquitoes to laboratory susceptible, the pyrethroid resistance locus *rp1* and SNPs around the ABC transporter ABCG4 were consistently significant, except for Uganda where SNPs in the P450 *CYP9K1* was markedly significant. In vitro heterologous metabolism assays with recombinant *CYP9K1* revealed that it metabolises type II pyrethroid (deltamethrin; 64% depletion) but not type I (permethrin; 0%), while moderately metabolising DDT (17%). *CYP9K1* exhibited reduced genetic diversity in Uganda underlying an extensive selective sweep. Furthermore, a glycine to alanine (G454A) amino acid change in *CYP9K1* was fixed in Ugandan mosquitoes but not in other *An*. *funestus* populations. This study sheds further light on the evolution of metabolic resistance in a major malaria vector by implicating more genes and variants that can be used to design field‐applicable markers to better track resistance Africa‐wide.

## 1 INTRODUCTION

Malaria control relies heavily on insecticide‐based interventions, notably long‐lasting insecticidal nets (LLINs) incorporating pyrethroid insecticides, and indoor residual spraying (IRS). Together, these interventions are credited with a greater than 70% decrease in malaria burdens since their introduction (Bhatt et al., 2015). However, unless resistance to insecticides is managed, the recent gains in reducing malaria transmission could be lost (Hemingway, 2017). Worryingly, several mosquito populations are developing multiple and cross‐resistance to a broad range of insecticides, increasing the risks that such populations could be better equipped to rapidly develop resistance to novel classes of insecticides. Therefore, elucidating the genetic basis and evolution of resistance is crucial to design resistance management strategies and prevent malaria resurgence (Hemingway, 2017).

In the major malaria vector *Anopheles funestus*, metabolic resistance mechanisms are driving resistance to most insecticides, including pyrethroids (Amenya et al., 2008; Riveron et al., 2013; Weedall et al., 2019). The molecular basis of this resistance is diverse and complex across Africa, with different resistance mechanisms spreading, and potentially intermixing, from independent origins (Barnes, Irving, et al., 2017; Djuicy et al., 2020; Riveron, Ibrahim, et al., 2014; Riveron, Yunta, et al., 2014; Weedall et al., 2020). These mechanisms are driven by extensive genetic variation between regions, preventing the use of existing findings to inform control efforts across the continent. Progress was recently made in this area through the detection of a DNA‐marker in the *cis*‐regulatory region of the cytochrome P450s *CYP6P9a and CYP6P9b* allowing the design of PCR assays for detecting and tracking pyrethroid resistance in the field (Mugenzi et al., 2019; Weedall et al., 2019). This resistance marker, however, only explains resistance in southern Africa (Weedall et al., 2019, 2020). This is a major obstacle in designing effective resistance management strategies across the continent, to better control this major malaria vector.

Transcriptomic analyses have successfully been used to detect key genes conferring resistance to insecticides in the principal malaria vectors (Ingham et al., 2018; Riveron et al., 2013; Weedall et al., 2019). Despite large‐scale whole genome sequencing, it has proven difficult to conclusively associate variants with resistance. This indicates a need for a combination of sequencing methods followed by functional validation to detect metabolic resistance markers. Genome‐wide association of pooled individuals (GWAS‐PoolSeq) has successfully detected candidate genomic regions of specific phenotypes, including variation in pigmentation in *Drosophila* (Bastide et al., 2013). In *An*. *funestus*, we recently discovered a duplication of the X chromosome cytochrome P450 *CYP9K1* associated with increased gene expression using this method (Weedall et al., 2020). Deep sequencing of target‐enriched data has successfully been implemented to elucidate mechanisms of insecticide resistance in the dengue mosquito vector, *Aedes aegypti* (Faucon et al., 2015). Therefore, a GWAS‐PoolSeq approach in tandem with targeted enrichment of candidate genomics regions could offer further opportunities to elucidate the complexities of metabolic resistance in *An*. *funestus*, while also helping to detect causative resistance alleles.

Here, we used a multi‐omics approach combining GWAS‐PoolSeq and target enrichment with deep sequencing experiments to elucidate the molecular basis of pyrethroid resistance in the major malaria vector *An*. *funestus*. The genome‐wide association study used pooled mosquitoes with binary “resistant” or “putatively susceptible” phenotypes from two locations representing Southern and Central Africa, respectively. The fine‐scale targeted sequencing approach was used to enrich a portion of the genome of individual mosquitoes Southern, Central and East Africa. The set of genes targeted represent many candidate metabolic resistance loci and previously identified resistance‐associated loci. In the target enrichment data, we identified an allele of the X‐linked cytochrome P450 gene *CYP9K1* probably driving pyrethroid resistance in East Africa. In vitro heterologous expression of *CYP9K1* in *E*. *coli* revealed this P450 capable of efficiently metabolising the type II pyrethroids deltamethrin.

## 2 MATERIALS AND METHODS

### 2.1 Design of SureSelect baits

The sequence capture array was designed prior to the release of the *An*. *funestus* genome assembly, using a mix of de novo assembled *An*. *funestus* transcripts (Crawford et al., 2010; Gregory et al., 2011) selected from previous pyrethroid resistance microarray experiments (Riveron, Ibrahim, et al., 2014; Riveron et al., 2013). Among these were heat shock proteins (HSPs), Odorant Binding Proteins and immune response genes such as serine peptidases, *Anopheles gambiae* detoxification genes sequences (282 genes) and all target‐site resistance genes sequences from *An*. *funestus*. We also included the entire genomic regions of the major quantitative trait locus (QTLs) associated with pyrethroid resistance which are the 120 kb BAC clone of the *rp1* containing the major *CYP6* P450 cluster on chromosome 2 (right arm), as well as the 113 kb BAC clone sequence for the *rp2* chromosome 2 (left arm). A total of 1302 target sequences were included (with redundancy). Baits were designed using the SureSelect DNA Advanced Design Wizard in the eArray program of Agilent. The bait size was 120 bp for paired‐end sequencing using the “centred” option with a bait tiling frequency (indicating the amount of bait overlap) of “x3”.

### 2.2 Collection, rearing and sequencing of mosquitoes

Two *An*. *funestus* laboratory colonies (the FANG and FUMOZ) and field mosquitoes from Cameroon, Malawi and Uganda were utilised in this study. The FANG colony is a fully insecticide susceptible colony derived from Angola (Hunt et al., 2005). The FUMOZ colony is a multi‐insecticide resistant colony derived from southern Mozambique (Hunt et al., 2005). Field populations of mosquitoes representative of Central, East and southern Africa were sampled from Mibellon (6°46′N, 11°70′E), Cameroon in February 2015; in March 2014 from Tororo (0°45′N, 34°5′E), Uganda (Mulamba, Riveron, et al., 2014) and in January 2014 from Chikwawa (16°1′S, 34°47′E), southern Malawi (Riveron et al., 2015). Mosquitoes were kept until fully gravid and forced to lay eggs using the forced‐egg laying method (Morgan et al., 2010). All *F*_0_ females/parents that laid eggs were morphologically and molecularly identified as belonging to the *An*. *funestus* group according to a morphological key and cocktail PCR, respectively (Gillies & Coetzee, 1987; Koekemoer et al., 2002). Egg batches were transported to the Liverpool School of Tropical Medicine under a DEFRA licence (PATH/125/2012). Eggs were allowed to hatch in cups and mosquitoes reared to adulthood in the insectaries under conditions described previously (Morgan et al., 2010). Insecticide resistance bioassays on these samples have been previously described (Mulamba, Riveron, et al., 2014; Riveron et al., 2015, 2016). In summary, two‐to‐five‐day old *F*_1_ females were exposed to permethrin for differing lengths of time to define a set of putatively susceptible (dead after 60 min permethrin exposure for Malawi and Uganda populations, and 20 min for Cameroon) and resistant (alive after 180 min permethrin exposure; 60 min in Cameroon) mosquitoes. The variation of exposure time was associated with the level of resistance in the population.

For the PoolSeq experiment, there were sufficient individuals for two likely “susceptible” and three “resistant” replicates of 40 individuals each from Malawi and one “susceptible” and one “resistant” replicate also of 40 individuals from Cameroon. Genomic DNA was extracted per individual using the DNeasy Blood and Tissue kit (Qiagen) and individuals were species ID molecularly (Koekemoer et al., 2002) and pooled per replicate in equal amounts. Library preparation and whole‐genome sequencing by Illumina HiSeq2500 (2 × 150 bp paired‐end) was carried out by the Centre for Genomic Research (CGR), University of Liverpool, UK. The SureSelect experiment consisted of 10 putatively permethrin susceptible and 10 resistant mosquitoes from Malawi (Southern), Cameroon (Central) and Uganda (Eastern) Africa from the set used for the PoolSeq, above. An additional 10 mosquitoes from the completely susceptible FANG strain were also included. The library construction and capture were performed by the CGR using the SureSelect target enrichment custom kit with the 41,082 probes. Libraries were pooled in equimolar amounts and paired‐end sequenced (2 × 150 bp) with 20 samples per run of an Illumina MiSeq by CGR, using v4 chemistry.

### 2.3 Population genomic pipelines

#### 2.3.1 Analysis of PoolSeq data

The PoolSeq data was analysed in the R package poolfstat (Gautier et al., 2021) and popoolation2 (Kofler et al., 2011). For poolfstat, PoolSeq R1/R2 read pairs were aligned to the VectorBase version 52 *An*. *funestus* reference sequence using bwa (Li & Durbin, 2009). Output BAM alignment files were coordinate sorted and duplicates marked in picard (http://broadinstitute.github.io/picard). For *F*_ST_ analyses, variant calling was carried out using varscan (2.4.4) (Koboldt et al., 2012), with a minimum variant frequency of 0.01 and *p*‐value of .05 and filtered in bcftools (1.9) (Danecek et al., 2021) to retain only SNPs greater than 3 bp away from predicted indels and the resulting the VCF file input to poolfstat. For intra‐Malawi and Cameroon “resistant versus susceptible” comparisons average *F*_ST_ was calculated pairwise between replicates and summarised into nonoverlapping 1000 bp windows using windowscanr (https://github.com/tavareshugo/WindowScanR/). Additionally, average *F*_ST_ for nonoverlapping sliding windows of 1000 SNPs was calculated in poolfstat for (i) all Malawi replicates combined and (ii) Cameroon versus Malawi replicates.

For Popoolation 2 analyses, a sync file was created from a samtools mpileup (version 1.12) and separate comparisons of “Malawi Dead (2×) versus Alive (3×) replicates” and “Cameroon versus Malawi” input to the Cochran‐Mantel‐Haenszel (CMH) test script “CMH‐test.pl”. The “Cameroon versus Malawi” comparison served as a positive control for this method as we expected to see a strong peak around the *rp1* locus according to prior research (Weedall et al., 2019, 2020). Only sites with total coverage greater than 10‐fold and lower than the 95th coverage percentile for each sample were considered. This test uses multiple independent pairwise comparisons to identify the signals common to all. Here, independent exposure assays were used to generate the dead and the alive mosquitoes, therefore any pair of samples used to generate a 2 × 2 contingency table is arbitrary. Using all six possible pairwise combinations of the two Dead and three Alive samples means that the 2 × 2 tables are not independent of one another and violates the assumptions of the test. This test was run, however, to compare the results with those of combined and pairwise *F*_ST_ tests and with the intention of functionally validating inferences. We also ran a two‐way independent CMH test of “Alive 1” versus “Dead 1” and “Alive 2” versus “Dead 2” replicates for comparison with the CMH test incorporating all replicates. There were six runs of the test made each with two different, independent pairwise combinations of dead and alive samples. Genome‐wide *F*_ST_ and ‐log_10_ *p*‐value plots were created in R using ggplot2 (Wickham, 2016) for poolfstat and Popoolation results, respectively.

#### 2.3.2 Analysis of SureSelect data

Initial processing and quality assessment of the sequenced data was performed as for the PoolSeq data and analysed using strandngs 3.4 (Strand Life Sciences). Alignment and mapping were performed using the “DNA alignment” option against the whole genome (version AfunF1) which was constructed into three chromosomes using synteny from *An*. *gambiae* (Weedall et al., 2019, 2020). To summarise Weedall et al. (2020), the 1392 AfunF1 *Anopheles funestus* genome assembly contigs were ordered relative to *Anopheles gambiae* (AgamP4) chromosomes using nucmer, from mummer v3.0. Nucmer alignment placed 46% (644 of 1392), totalling 217,255,185 bp or 96% (225,223,604 bp) of the AfunF1 assembly (96%). For reference, the AfunF3 chromosomal assembly is slightly shorter in length at 210,975,222 bp as of VectorBase release 56. The 2L to 3R transposition between *An*. *gambiae* and *An*. *funestus* was accounted for by renaming of the ordered contigs and unplaced scaffolds were placed at the end of the ordered contigs. Aligned and mapped reads were used to create a DNA variant analysis experiment. Before variant detection, SNP preprocessing was performed to reduce false positive calls: (i) split read realignment of partially aligned split reads and full‐length reads with gaps introduced by initial alignment, (ii) local realignment to reduce alignment artefacts around indels, and (iii) base quality score recalibration to reduce errors and systematic bias.

All variant types (SNPs, MNPs [multiple nucleotide polymorphisms] and indels) were detected by comparing against the FUMOZ AfunF1 genome using the MAQ independent model implemented in strandngs 3.4 and default parameters. A SNP multi sample report was generated for each sample. For each variant, its effect was predicted using the transcript annotation (version AfunF1.4). To identify SNPs significantly associated with permethrin resistance, two approaches were used. First, we used a differential allele frequency‐based approach where a variant was significant in relation to permethrin resistance if the supporting read of the SNP is found in 35%–100% of the alive mosquitoes (R) after permethrin exposure but present at low frequency in dead mosquitoes (1%–35%) (C) (R‐C comparison). Both sets of mosquitoes were also compared to the fully susceptible laboratory colony, FANG (S), with significant SNPs having frequency >35% but <35% in FANG (S) in R‐S and C‐S comparisons. This assumes that SNPs associated with resistance will be present at higher frequency in alive mosquitoes (>35%) but lower in dead (<35%). Additionally, a minimum threshold of five out of 10 individuals of the same category was required to include a SNP. The second approach assessed the significant association between each variant and permethrin resistance by estimating the unpaired t‐test unpaired of each variant between each comparison (R‐C, R‐S and C‐S) and a Manhattan plot of –Log_10_ of *p*‐value created. A SNP frequency cutoff of three or more samples was applied for this approach.

Finally, the polymorphism pattern of the *CYP9K1* gene was analysed across Africa using the sureselect data. *CYP9K1* polymorphisms were retrieved from the SNP Multisample report file generated through Strand NGS 3.4 for each population. Bioedit (Hall, 1999) was used to input various polymorphisms in the VectorBase reference sequence using ambiguity codes to indicate heterozygote positions. Haplotype reconstruction and polymorphism analyses were made using dnaspv5.10 (Librado & Rozas, 2009). mega x (Kumar et al., 2018) was used to construct the maximum likelihood phylogenetic tree for *CYP9K1*.

### 2.4 Heterologous expression of recombinant CYP9K1 and metabolic assays

#### 2.4.1 Amplification and cloning of full‐length cDNA of *An*. *funestus CYP9K1*

RNA was extracted using the PicoPure RNA isolation Kit (Arcturus, Applied Biosystems, USA) from three pools each of 10 permethrin‐resistant females from Tororo in Uganda. The RNA was used to synthesize cDNA using SuperScript III (Invitrogen) with oligo‐dT20 and RNAse H (New England Biolabs). Full length coding sequences of *CYP9K1* were amplified separately from cDNA of 10 mosquitoes using the Phusion HotStart II Polymerase (Thermo Fisher) (primers sequences: Table S1). The PCR mixes comprised of 5× Phusion HF Buffer (containing 1.5 mM MgCl_2_), 85.7 µM dNTP mixes, 0.34 µM each of forward and reverse primers, 0.015 U of Phusion HotStart II DNA polymerase (Fermentas) and 10.71 µl of dH_2_0, 1 µl cDNA to a total volume of 14 µl. Amplification was carried out using the following conditions: one cycle at 98°C for 1 min; 35 cycles each of 98°C for 20 s (denaturation), 60°C for 30 s (annealing), and extension at 72°C for 2 min; and one cycle at 72°C for 5 min (final elongation). PCR products were cleaned individually with QIAquick PCR Purification Kit (Qiagen) and cloned into pJET1.2/blunt cloning vector using the CloneJET PCR Cloning Kit (Fermentas). These were used to transform cloned *E*. *coli DH5α*, plasmids miniprepped with the QIAprep Spin Miniprep Kit (Qiagen) and sequenced on both strands using the pJET1.2F and R primers provided in the cloning kit.

#### 2.4.2 Cloning and heterologous expression of *An*. *funestus* CYP9K1 in *E*. *coli*

The pJET1.2 plasmid bearing the full‐length coding sequence of *CYP9K1* was used to prepare the P450 for expression by fusing it to a bacterial *ompA*+*2* leader sequence allowing translocation to the membrane following previously established protocols (Ibrahim et al., 2016; Pritchard et al., 1997). This fusion was achieved in a PCR reaction using the primers given in Table S1. Details of these PCRs are provided in previous publications (Ibrahim et al., 2016; Riveron, Ibrahim, et al., 2014). The PCR product was cleaned, digested with *Nde*I and *Xba*I restriction enzymes and ligated into the expression vector pCWori+already linearized with the same restriction enzymes to produce the expression plasmid, pB13::*ompA*+*2*‐*CYP9K1*. This plasmid was cotransformed together with *An*. *gambiae* cytochrome P450 reductase (in a pACYC‐AgCPR) into *E*. *coli JM109*. Membrane expression and preparation was performed as for (Pritchard et al., 2006). Recombinant *CYP9K1* was expressed at 21°C and 150 rpm, 48 h after induction with 1 mM IPTG and 0.5 mM δ‐ALA to the final concentrations. Membrane content of the P450 and P450 reductase activity were determined as previously established (Omura & Sato, 1964; Strobel & Dignam, 1978).

#### 2.4.3 In vitro metabolism assays with insecticides

Metabolism assays were conducted with permethrin (a type I pyrethroid insecticide), deltamethrin (a type II) and the organochlorine DDT. Assay protocols have been described previously (Ibrahim et al., 2018; Riveron, Ibrahim, et al., 2014). Then, 0.2 M Tris–HCl and NADPH‐regeneration components were added to the bottom of chilled 1.5 ml tubes. Membranes containing recombinant *CYP9K1* and *Ag*CPR were added to the side of the tube to which cytochrome b_5_ was already added in a ratio 1:4 to the concentration of the *CYP9K1* membrane. These were preincubated for 5 min at 30°C, with shaking at 1200 rpm and then 20 µM of test insecticide was added into the final volume of 0.2 ml (~2.5% v/v methanol), and the reaction started by vortexing at 1200 rpm and 30°C for 90 min. Reactions were quenched with 0.1 ml ice‐cold methanol and incubated for 5 min to precipitate protein. Tubes were centrifuged at 16,000 rpm and 4°C for 15 min, and 100 µl of supernatant and transferred into HPLC vials for analysis. All reactions were carried out in triplicate with experimental samples (+NADPH) and negative controls (−NADPH). Per sample volumes of 100 µl were loaded onto isocratic mobile phase (90:10 v/v methanol to water) with a flow rate of 1 ml/min, a wavelength of 226 nm and peaks separated with a 250 mm C18 column (Acclaim 120, Dionex) on an Agilent 1260 Infinity at 23°C. For DDT, a solubilizing agent sodium cholate (1 mM) was added as described in Mitchell et al. (2012) and absorption monitored at 232 nm. Enzyme activity was calculated as percentage depletion (difference in the amount of insecticide remaining in the +NADPH tubes compared with the –NADPH) and a *t*‐test used to assess significance.

## 3 RESULTS

### 3.1 Genome‐wide association study with pooled mosquitoes to identify allelic variants putatively associated with permethrin resistance

**FIGURE 1:**
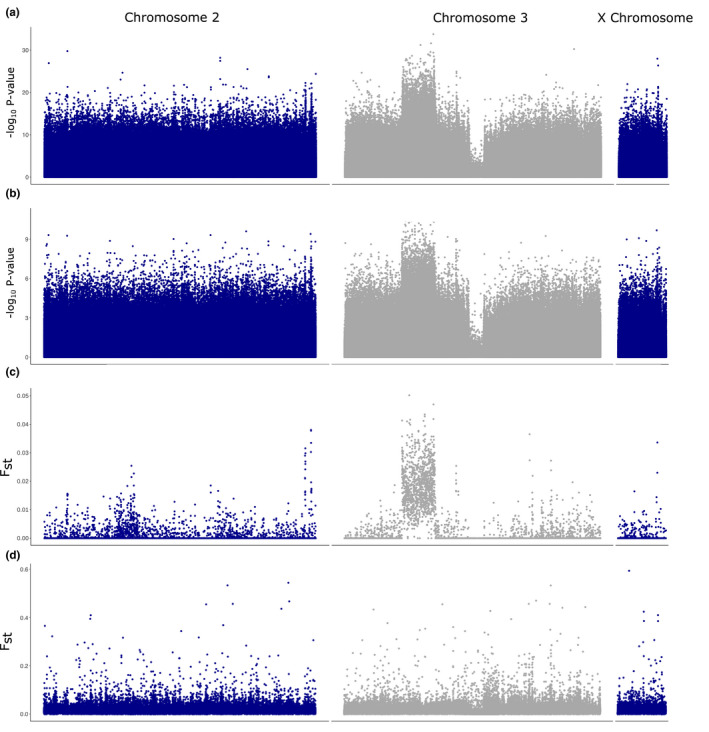
PoolSeq genome‐wide analysis between pools of permethrin resistant and susceptible *An*. *funestus* from Malawi and Cameroon. (a) Cochran‐Mantel‐Haenszel test –log_10_ *p*‐values per SNP calculated in Popoolation from all six possible pairwise comparisons 2 in Malawi, (b) for two pairs of replicates with independence, (c) *F*_ST_ values for 1000 bp windows calculated in poolfstat for Malawi replicates and (d) for Cameroon replicates

Sequence data obtained for each F_1_ pool were first quality controlled (trimming, pair‐end) and aligned to the *An*. *funestus* F3 FUMOZ reference genome (Table S2) (Ghurye et al., 2019). To identify variant sites with allele frequencies significantly associated with the phenotypes in Malawi (dead [D] after 60 min permethrin exposure [*n* = 2 pools] and alive [A] after 180 min permethrin exposure [*n* = 3 pools]), Cochran‐Mantel‐Haenszel tests of association and divergence (*F*_ST_) estimates were applied to all biallelic variant sites. These estimates were plotted as ‐log10 *p*‐values Manhattan plots for 1000 SNP sliding‐window global *F*_ST_s estimated in the R package poolfstat (Gautier et al., 2021) and Cochran Mantel Tests of association (Figure 1a–c) using popoolation2 (Kofler et al., 2011). GWAS results for Malawi were consistent between Cochran‐Mantel‐Haenszel (CMH) tests of association for all six possible comparisons (Figure 1a) or two independent comparisons (Figure 1b) and global *F*_ST_. Discussion of CMH tests results were therefore restricted to the all‐replicate six‐way analysis (Figure 1a), although please note the difference in *y*‐axis scale between Figure 1a,b. For both analyses, a 12 megabase‐long region of elevated *F*_ST_/‐log_10_ *p*‐value is observed between 21 and 33 Mb on chromosome 3 in Malawi. This extensive region is annotated with 765 genes many of which are of unknown function (242) but does include six cuticular genes and one cytochrome P450 (*CYP301A1*). The average *F*_ST_ in this region is 0.018 versus a background of *F*_ST_ of 0.0005 for chromosome 3. Although a substantial 32‐fold difference in *F*_ST_ averages, the absolute *F*_ST_ of this region was low. Furthermore, on inspection of the pairwise *F_st_* plots (Figure S1), this elevated region was observed in “Alive1” and “Alive3” versus dead replicates but not for the “Alive2” replicate. Two peaks on chromosome 2 around positions 95.6 and 97.7 are prominent in the *F*_ST_ results and can also be discerned in the CMH plot (Figure 1a). The first region of elevated *F*_ST_ from positions 95,515,427 to 95,668,792 is composed of 40 genes including *CYP9M1* (AFUN015938) and *CYP9M2* (AFUN016005). There were also four cellular retinaldehyde binding proteins, three CRAL‐TRIO domain‐containing proteins and the remaining 31 genes lacked annotation. The peak around 97.7 Mb did not overlap any gene but is downstream of the 3′ end of gene AFUN003294 which encodes an ETS family transcriptional repressor. The only other visually concordant region of *F*_ST_ and −log_10_ *p*‐values was observed towards the end of the X chromosome from positions 14.4 to 14.7 mb overlapping four genes including a homologue of “single‐minded” (AFUN005600) and an unannotated gene (AFUN020237) with homology to “stasimon”. Although the SNP with the highest CMH ‐log_10_ *p*‐value is outside of this region at position 14,172,028.

To test if similar results were observed in Cameroon, an *F*_ST_ only analysis was performed as only one Dead and one Alive replicate were sequenced. Background *F*_ST_values were low and similar for all three chromosomes at 0.015, 0.015 and 0.017 for chromosomes 2, 3 and the X chromosome, respectively (Figure 1d), although several fold larger than background chromosomal *F*_STs_ in the Malawi data. Outliers were few and did not overlap with those for Malawi.

Finally, an intercountry comparison was made by poolfstat global *F*_ST_ and Popoolation2 CMH test. In contrast to intracountry comparisons a well‐defined peak of differentiation was observed across the *rp1* locus for both analyses (Figure S2). In addition, the X chromosome was of elevated background *F*_ST_ versus autosomes with an average *F*_ST_ of 0.165 versus 0.062 and 0.056 for chromosomes 2 and 3 respectively. Overall, because of the lack of strong candidate resistance variants detected with this PoolSeq GWAS approach, it was not pursued in other countries, but a fine‐scale approach was employed instead.

### 3.2 Detection of variants associated with pyrethroid resistance using SureSelect targeted enrichment and deep sequencing

A total of 3,059,528 bp of the 1302 sequence capture regions was successfully sequenced in 70 individual mosquitoes (Tables S3–S5). Mapping and coverage metrics of the targeted sequencing relative to the reference genome were within expectation (Tables S4 and S5). The good quality of the target enrichment is also supported by the average base quality of the reads, the alignment score of the mapped reads and the match status of paired ended reads for each sample (Figure S3). Integrative Genomics Viewer (IGV) (Thorvaldsdottir et al., 2013) was used to visually inspect the alignment results showing that in general, sequence capture regions were well covered and lower level coverage was seen between these regions. A total of 137,137 polymorphic sites were detected across all three countries plus the fully susceptible FANG laboratory colony. Analysis performed between each country and FANG detected 75,980, 79,095 and 38,380 polymorphic sites respectively in Cameroon, Uganda and Malawi. Lower polymorphism between Malawi and the reference genome (FUMOZ, originally sampled in southern Mozambique) than Cameroon and Uganda reflect their shared southern African origin. Detection of SNPs significantly associated with permethrin resistance was performed first using the differential SNP frequency analysis implemented in Strand NGS (Strand Life Sciences, Bangalore, India).

#### 3.2.1 Cameroon

**FIGURE 2:**
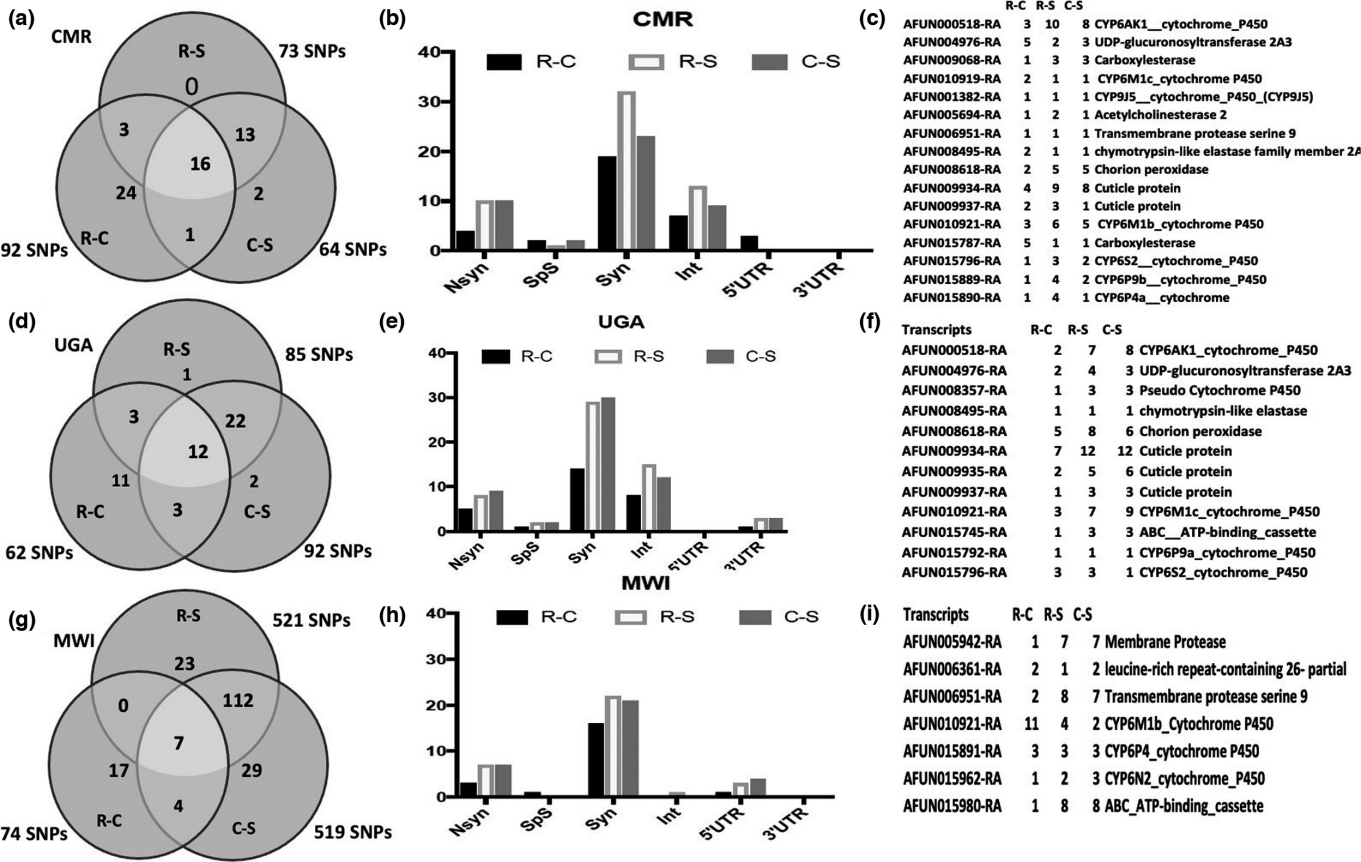
Variants significantly associated with permethrin resistance using SureSelect target enrichment sequencing of specific candidate resistance genomic regions. Using a frequency‐based filtering approach implemented in StrandNGS, (a) sets of SNPs significantly associated with resistance were detected in various comparisons between field Permethrin Alive (R) and dead (C) and the susceptible laboratory strain FANG (S) in Cameroon. (b) Distribution of the significant SNPs between nonsynonymous (Nsyn), splice sites (SpS), synonymous (Syn), intron (Int), 5’untranslated region (5’UTR) and 3’ untranslated regions (3’UTR) in Cameroon. (c) List of genes with variants significantly associated with permethrin resistance in Cameroon. (d), (e), (f) are equivalent of (a), (b) and (c) for Uganda, respectively, as are (g), (h) and (i) for Malawi

Using the frequency‐based filtering approach, 92 SNPs out of the 75,980 polymorphic sites were found to be significant between resistant and putatively susceptible field mosquitoes (R‐C), 73 between resistant and FANG strain (R‐S), and 64 between Cameroon susceptible and FANG (Figure 2a). Most of these SNPs were silent substitutions followed by intronic and nonsynonymous ones (Table S6; Figure 2b). We considered the best candidate SNPs to be those present in all three comparisons. These common SNPs belong to 16 genes (Figure 2c) including seven cytochrome P450s in the known major pyrethroid resistance QTLs notably *rp1* (*CYP6P4a*,*CYP6P9b*) on chromosome 3, *rp2* (*CYP6M1b*,*CYP6M1c*,*CYP6S2*) on chromosome 2, as well as *rp3* (*CYP9J11*) on chromosome 3. Further evidence of the association of polymorphisms at *rp1* with the resistance phenotype was the presence of the carboxylesterase gene (AFUN015787) located within this same genomic region. Two cuticle protein genes also presented abundant significant SNPs (AFUN009934 and AFUN009937) for all three comparisons. Two genes showed common amino acid changes for all three comparisons, the P450 *CYP6AK1* (AFUN000518) and the UDP‐glucuronosyl transferase (AFUN004976). For the resistant versus field‐susceptible (R‐C), four significantly different nonsynonymous sites were identified in the immune response gene *APL1C* followed by three in carboxylesterase (AFUN015787) (Table S6). Further immune response genes such as chymotrypsin‐like elastase and serine proteases contained significant nonsynonymous R‐C changes.

#### 3.2.2 Uganda

Fewer SNPs (62 of 79,095 polymorphic sites) were found to be significant between Ugandan permethrin resistant and field‐susceptible mosquitoes (R‐C), with 85 between resistant and the FANG (R‐S) and 92 between Ugandan susceptible and FANG (C‐S, Figure 2d). As for Cameroon most of these SNPs were silent substitutions followed by intronic and nonsynonymous SNPs (Figure 2e). SNPs present in all three comparisons belong to 12 genes (Figure 2f) including four P450s from the *rp1* QTL (*CYP6P9a* and the pseudo‐P450 AFUN008357) and *rp2* (*CYP6M1c* and *CYP6S2*). As for Cameroon, three cuticle protein genes had the most significant SNPs between the three comparisons. For nonsynonymous substitutions, two genes showed common amino acid changes in all three comparisons, the P450 *CYP6AK1* (AFUN000518) and a cuticle protein (AFUN009934). As for Cameroon, APL1C had the most nonsynonymous substitutions in the R‐C comparison (Table S6) and other immune response genes were present in this comparison.

#### 3.2.3 Malawi

Despite the lower overall number of polymorphisms, 74 significant SNPs were detected between Malawian permethrin resistant and susceptible field mosquitoes (R‐C) (Table S6), with 521 between Malawi resistant and the (R‐S) and 519 between Malawian susceptible and FANG (Table S7; Figure 2g). Due to the similarity of Malawi data to the reference sequence in contrast to Cameroon and Uganda, significant SNPs have been detected assuming a higher frequency in the field‐susceptible than the resistant. Intergenic SNPs common to all three comparisons belong to seven genes (Figure 2i) including three P450s from the *rp1* (*CYP6P4a*) and *rp2* (*CYP6M1b* and *CYP6N2*) QTLs. The gene with the most significant SNPs is the P450 *CYP6M1b*. The gene with most nonsynonymous substitutions in the R‐C comparison alone was the P450 *CYP6AK1* with three sites followed by the cuticle protein (AFUN009936) and cytochrome P450 *CYP4H19* (AFUN001746) with two such sites each (Table S6). As in Cameroon and Uganda, SNPs in immune response genes were also found in all comparisons.

A second approach consisted of detecting significant SNPs by *t*‐test in each country which provided the following results.

#### 3.2.4 Cameroon

**FIGURE 3:**
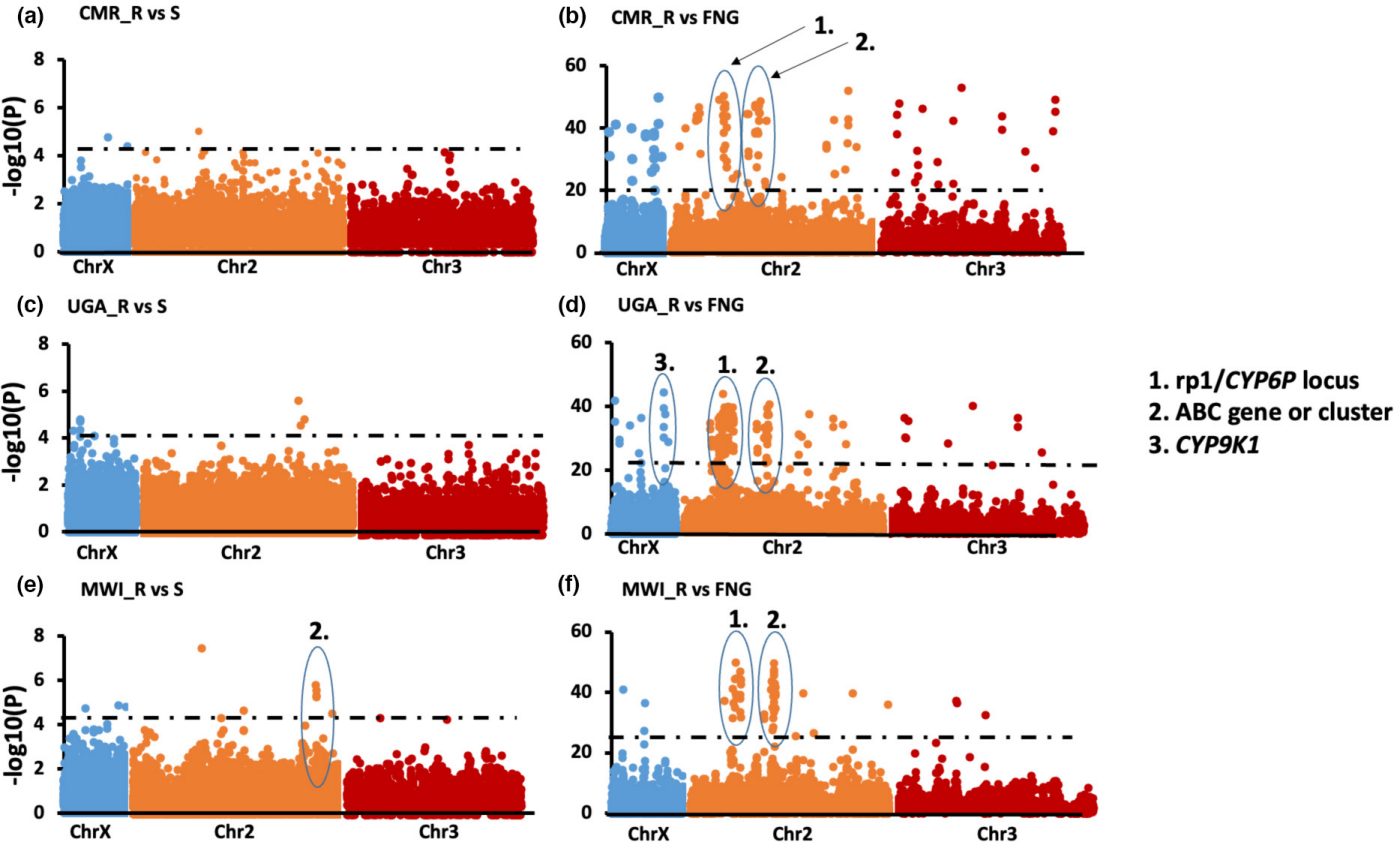
Variants significantly associated with permethrin resistance using an unpaired *t*‐test between the resistant mosquitoes (alive) and susceptible (Dead). (a) Significant variants between permethrin resistant and susceptible mosquitoes in Cameroon (unpaired *t*‐test); whereas (b) is between Cameroon resistant and FANG susceptible strain. (c) Is for Uganda Alive and Dead mosquitoes after permethrin exposure while (d) are the significant SNPs between the Uganda Alive and the susceptible laboratory strain (FANG) and (e and f) are for significant SNPs between Malawi Alive and Dead mosquitoes and versus FANG, respectively. SNPs located in the rp1 QTL resistance regions on chromosome 2 are consistently associated with pyrethroid resistance. Similarly, a cluster of ABC transporter genes including ABCG4. The black dotted line indicates multiple testing significance level *(p* = 5 × 10^−5^) for R‐C comparisons and (*p* = 5 × 10^−22^) for comparisons with FANG susceptible strain. Chr stands for chromosome and the legend indicates genes underlying highly‐significant *p*‐values

For SNPs present in three or more mosquitoes, the most highly significant SNP was in the cytochrome P450 *CYP304B1* on chromosome 2 (*p* = 1.7 × 10^−5^). Analysis of the 29 SNPs with Bonferroni‐corrected *p* < .001 revealed seven SNPs that were also detected with the frequency‐based filtering approach above (Table S8; Figure 3a) including a SNP located in chorion peroxidase (AFUN00618) and the cytochrome P450 *CYP6M1c* (AFUN010919) on the *rp2* QTL. Some of these 29 SNPs also belong to genes known to be significantly overexpressed in resistant mosquitoes such the P450 *CYP315A1* and the glutathione S‐transferase *GSTe3* (Weedall et al., 2019). Three nonsynonymous SNPs were detected belonging to the P450 *CYP304B1* (amino acid change: I504V), the chymotrypsin‐like protease (AFUN015111) (D476G) and the decarboxylase, AFUN007527 (V169L). A comparison of the 10 resistant Cameroon mosquitoes to FANG detected a lowest *p*‐value of 7.8 x 10^−48^ in a cuticular protein gene (AFUN004689). Regions with most SNPs between Cameroon and FANG were found in the *rp1* QTL, a Zinc finger protein (AFUN015873) and a cluster of ABC transporter genes around ABCG4 (Table S9; Figure 3b). This cluster of ABC transporter genes were also detected in the *t* test R‐C comparison.

#### 3.2.5 Uganda

When the most common SNPs only were analysed as for Cameroon, the most highly significant SNP was in the cytochrome P450 *CYP315A1* (AFUN005715) on the X chromosome *(p* = 2.9 × 10^−6^) (Table S10), which is also significantly overexpressed in resistant mosquitoes (Weedall et al., 2019). Six nonsynonymous SNPs were detected with some belonging to detoxification genes such as the P450 *CYP6AG1* (K262Q), or to immune response genes such as the transmembrane protease serine 13 (AFUN003078) (H61Y), serine protease 14 (AFUN000319) (N18H), chymotrypsin‐like elastase (AFUN015884) (T40K) and the C‐type lectin AFUN002085 (L63R). A comparison of the resistant mosquitoes from Uganda to FANG determined an intergenic substitution between the P450 gene *CYP6P9a* and a carboxylesterase gene (AFUN015793) in the *rp1* QTL region as most significant *p*‐value of 2.28 × 10^−50^ (Table S11; Figure 3d). Overall, most significant SNPs between Uganda and FANG were found around the pyrethroid resistant QTL *rp1* and a cluster of ABC transporter genes around ABCG4 (Figure 3d). Interestingly, we saw a peak of significant SNPs around the P450 *CYP9K1* on the X chromosome gene, a gene that has undergone a selective sweep and is highly expressed in Uganda (Weedall et al., 2020).

#### 3.2.6 Malawi

Among the 59 significant SNPs between resistant and susceptible (R‐C) Malawi mosquitoes the top significant was a synonymous substitution in the ABC transporter gene (ABCG4/AFUN007162) on chromosome 3 (*p*‐value of 3.0 × 10^−8^) (Table S12). Nonsynonymous SNPs were detected in the detoxification gene xanthine dehydrogenase (AFUN002567) (Q799E) and immune response Toll‐like receptor (AFUN002942) (V104M). A comparison of resistant Malawi mosquitoes to FANG with a lowest *p*‐value of 1.7 × 10^−45^ corresponding to a synonymous substitution in the P450 gene *CYP6P2* in the *rp1* QTL region, alongside a cluster of significant hits (Table S13; Figure 3f). Another cluster of significant hits is also detected around the ABCG4 gene which is also significant between the R‐C comparisons (Figure 3f).

### 3.3 Heterologous expression of *An. funestus* CYP9K1 in *E. coli*

#### 3.3.1 Expression pattern of recombinant CYP9K1

**FIGURE 4:**
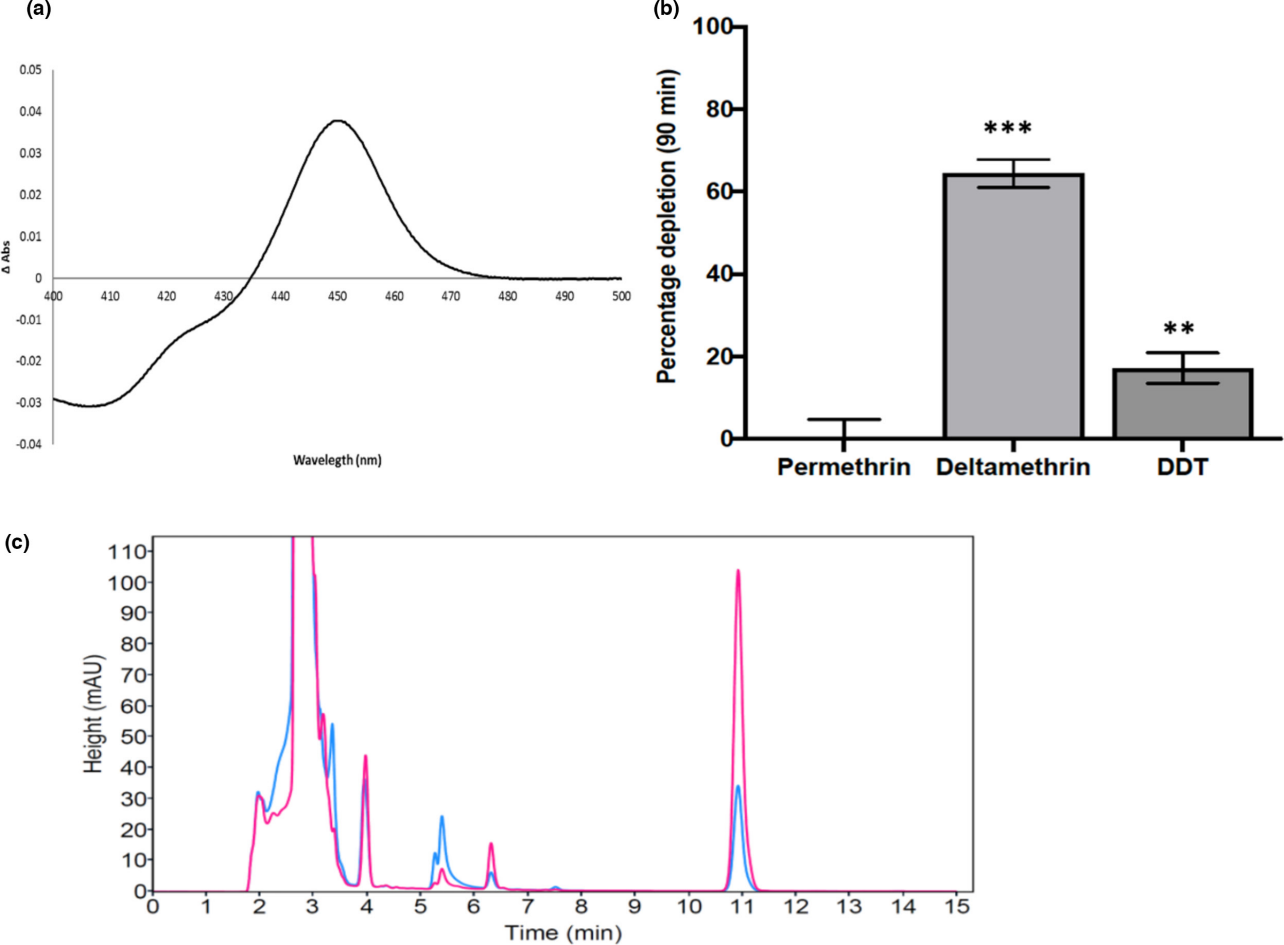
Metabolism of insecticides by recombinant *An*. *funestus* CYP9K1. (a) CO‐difference spectrum generated from *E*. *coli* membranes expressing CYP9K1. (b) Percentage depletion of various insecticides (20 µM) with recombinant CYP9K1; results are average of three replicates compared with negative control (–NADPH); *** Significantly different from –NADPH at *p* < .005. (c) Overlay of HPLC chromatogram of the CYP9K1 metabolism of deltamethrin, with –NADPH in pink and +NADPH in blue

A standard P450 carbon monoxide (CO) ‐difference spectrum was obtained when *CYP9K1* was coexpressed with cytochrome p450 reductase (CPR) in *E*. *coli*, as expected from a good‐quality functional enzyme with a predominant expression at 450 nm and low P420 content (Figure 4a). Recombinant *CYP9K1* expressed with a P450 concentration of ~1.2 nM at 48 h, and a P450 content of 0.93 nmol/mg protein. The membranous P450 reductase activity was calculated as 52.04 cytochrome *c* reduced/min/mg protein.

#### 3.3.2 *An. funestus* CYP9K1 metabolism of insecticides

Recombinant *CYP9K1* exhibited contrasting activity towards permethrin (type I) and deltamethrin (type II). While no metabolic activity was observed with permethrin (0.47% depletion), *CYP9K1* depleted 64% (64.37.5% ± 3.44, *p* < .01) of deltamethrin in 90 min (as determined by the disappearance of substrate [20 µM] after 90 min) compared to controls (with no NADPH) (Figure 4b,c). For DDT, a depletion of only 17% was observed, with no peak for either dicofol (kelthane) or DDE.

### 3.4 Analysis of *CYP9K1* polymorphism across Africa

#### 3.4.1 Comparative analysis of *CYP9K1* polymorphism in resistant, field and laboratory susceptible mosquitoes

**FIGURE 5:**
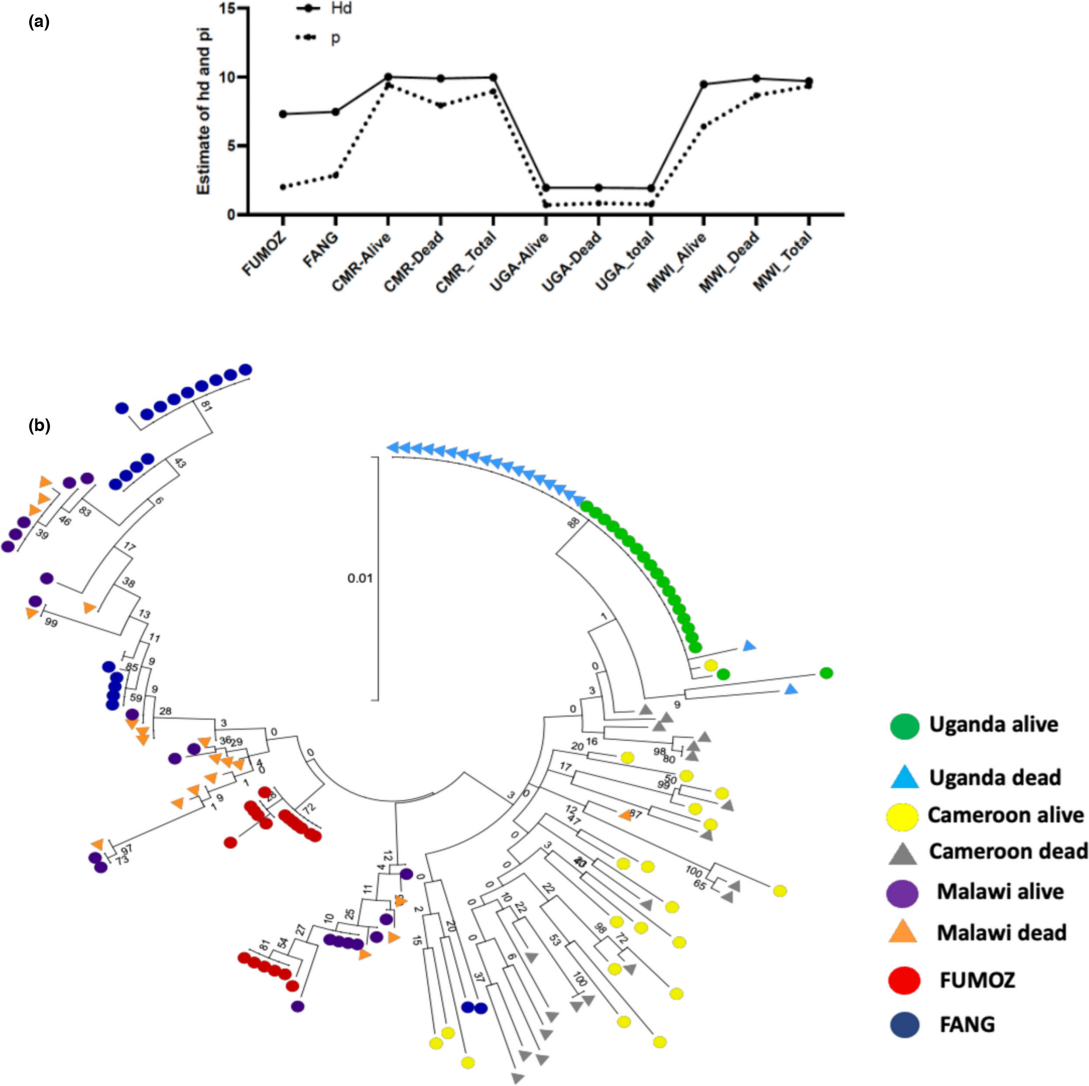
Polymorphisms patterns of *CYP9K1* in Africa. (a) Plot of genetic diversity parameters of *CYP9K1* across Africa showing the signature of a strong directional selection of *CYP9K1* in Uganda. Hd is for haplotype diversity while Pi is nucleotide diversity. (b) Phylogenetic tree for *CYP9K1* full‐length (2707 bp) between Fang, FUMOZ and resistant strains of Uganda, Cameroon, Malawi and FUMOZ using sureselect data

A 2707 bp genomic fragment spanning the full *CYP9K1* gene (5′UTR, 3′UTR, two exons and one intron) was analysed between 10 permethrin‐resistant and 10 susceptible mosquitoes from each of the three countries and from FANG. Analysis of these 70 mosquitoes revealed 137 substitutions and 72 haplotypes of the 2.7 kb gene‐body of *CYP9K1* across the continent. When mosquitoes were analysed by country, however, a stark contrast was observed between Uganda and other samples. This was evident for most parameters assessed, notably the lower number of substitution sites in Uganda (35 overall) versus Cameroon (123) and Malawi (42). A similar paucity of haplotypes was observed, with just five haplotypes in Uganda versus 38 and 29 in Cameroon and Malawi, respectively. Not surprisingly therefore, haplotype diversity in Uganda was also very low (0.19) in contrast to Cameroon (0.99) and Malawi (0.97) (Table S14). Similar patterns for Uganda were observed for other parameters including nucleotide diversity (π), this is well illustrated in the plot of haplotype diversity and nucleotide diversity (Figure 5a). Furthermore, Uganda samples exhibited low diversity when compared to FANG and FUMOZ. Both dead and alive mosquitoes exhibited this low diversity in Uganda (Figure 5a). A similar pattern of reduced polymorphism was seen when considering only the coding region (1614 bp) (Table S15) or the non‐coding (introns plus UTRs; 1093 bp) (Table S16). Analysis of the coding region detected a nonsynonymous polymorphism, substituting glycine for alanine at position 454, a mutation which is present in all individuals from Uganda. This G454A change was detected at lower frequencies in Malawi (14/40) and in Cameroon (9/40). An analysis using the Cytochrome P450 Engineering Database (CYPED) (Fischer et al., 2007) reveals that this G454A mutation is between the meander and cysteine pocket, which should impact on activity/catalysis, as amino acids in this region stabilizes the heme structural core and supposed to be involved in interaction with P450 reductase.

#### 3.4.2 Phylogenetic tree

A maximum likelihood tree of *CYP9K1* sequences supported the high genetic diversity of this gene across the continent with several haplotypes clustering, mostly by their geographical origin (Figure 5b). While mosquitoes from other countries cluster randomly, the majority of those from Uganda belong to a major predominant haplotype (36 out of 40 sequences).

#### 3.4.3 CYP9K1 haplotype network

Analysis of the Templeton, Crandall and Sing (TCS) haplotype tree further highlighted the high polymorphism of *CYP9K1* across Africa with many singleton haplotypes separated by many mutational steps (>30 steps) (Figure S4a–b). The predominant haplotype “H1” was nearly fixed in Uganda (32/40) when considering the full‐length or also only the coding region (36/40). The fact that this H1 haplotype is shared by both alive and dead mosquitoes suggest that it is close to fixation in this population. In other countries most haplotypes are found as singletons (35 out of 40 in Cameroon; 22 out of 40 in Malawi) supporting the high diversity of *CYP9K1* in those locations in contrast to Uganda. This pattern is similar when only analysing the coding region (Figure S5a–b) or the noncoding (Figure S6a–b).

## 4 DISCUSSION

As malaria prevention still relies heavily on insecticide‐based interventions, it is essential to improve our understanding of the mechanisms driving resistance in malaria vectors to prolong the effectiveness of these tools by implementing suitable resistance management strategies. The present study used a multi‐omics approach, and one of these approaches detected that the cytochrome P450 *CYP9K1* is a major driver of pyrethroid resistance in East African populations of the major malaria vector *An*. *funestus*.

### 4.1 Genome‐wide association study with the PoolSeq approach probably needs more replications

The replicated PoolSeq‐based genome‐wide association study did not detect significant variants associated with resistance. This is contrary to the usefulness of this method previously in detecting variants associated with natural pigmentation variation in *Drosophila* (Bastide et al., 2013). Among possible reasons for the lack of sensitivity of this is the poor phenotype segregation in our samples from Malawi. Resistance to insecticide was already relatively high in this population reducing the ability to differentiate between resistant and field‐susceptible mosquitoes. Additionally, increasing the number of replicates could have increased power of detection unfortunately the high resistance level made it difficult to generate sufficient susceptible individuals per location. This was the case for the *Drosophila* pigmentation experiment where more replicates of larger pools of flies were analysed (Bastide et al., 2013), not available to us here as stated above. Although no significant resistance‐associated variants were detected by PoolSeq GWAS, the elevated region of *F*_ST_ on chromosome 3 segregating in Malawi aligns precisely with the known 3Rb inversion of *An*. *funestus* (Dia et al., 2013; Green & Hunt, 1980; Sharakhov et al., 2001), a region which contains six cuticular protein genes belonging to the RR‐2 family. Unfortunately, it is not possible to estimate inversion frequency with confidence from poolseq data. RR‐2 family genes were previously associated with the reduced penetration resistance mechanism (Balabanidou et al., 2018). This could indicate that the reduce penetration resistance mechanism through cuticle thickening is playing a role in resistance to pyrethroid in Malawi. It is notable that no hit was detected on chromosome 2 spanning the resistance to pyrethroid 1 QTL (*rp1*), which was observed between Malawi and Cameroon. This is probably due to the fixation of selected alleles at these two P450 genes (Riveron et al., 2013), and highlights a drawback in our binary alive versus dead phenotypes as a proxy for resistant and susceptible genotypes. This is similar to the case of knockdown resistance allele L1014F which being fixed in many populations of *An*. *gambiae* does not correlate with phenotype when using field samples mainly due to the high selection in these samples (Antonio‐Nkondjio et al., 2015; Kwiatkowska et al., 2013). The validity of the poolfstat and Popoolation 2 approaches was nevertheless confirmed by the southern (Malawi) versus Central Africa (Cameroon) analysis which detected differentiation at the *rp1* locus. This locus contains the *CYP6P9a* and *CYP6P9b* cytochrome P450 genes, which confer pyrethroid‐resistance and are under strong directional selection in southern African populations of *An*. *funestus* (Mugenzi et al., 2019, 2020; Weedall et al., 2019). Although statistically attractive, the replicated PoolSeq offers us little extra over intercountry comparisons of pooled‐sequencing as demonstrated by detection of the *rp1* locus here and prior studies (Weedall et al., 2019, 2020). Perhaps, a PoolSeq approach using a crossing of resistant strains to FANG could provide a more productive platform to detect genetic variants associated with related resistance as implemented in *Aedes aegypti* (Cattel et al., 2020).

### 4.2 Deep targeted sequencing of genomic regions spanning detoxification genes detects genetic variants of interest

A fine‐scale approach combining targeted enrichment and deep sequencing successfully detected variants associated with pyrethroid resistance. This was most evident when comparing resistant mosquitoes to the fully susceptible laboratory FANG strain than when alive and dead mosquitoes from the same location were compared. This low power of detection when comparing samples from the same locality is probably due to high level of resistance inducing a poor segregation between samples. If the high number of significant variants detected between resistant and susceptible strain could be due to a difference in genetic background, the fact that key genomic regions previously associated with resistance were clearly and consistently detected such as *rp1*, revealed the ability of this approach to detect resistance mutations. Indeed, the *rp1* QTL region harbouring a cluster of P450s involved in resistance such as *CYP6P9a*/*b*,*CYP6P4a*/*b*,*CYP6P5* was one of the major loci detected. This could explain why this region was significantly associated with resistance in all regions since at least one gene from this region is over‐expressed in each region with *CYP6P5* in Cameroon and Uganda, *CYP6P9a*/*b* in Malawi (Mugenzi et al., 2019; Weedall et al., 2019). Furthermore, a consistent resistance locus in all three countries when compared to FANG was associated with the ABC transporter gene ABCG4 (AFUN016161‐RA) located in the vicinity of two other ABC genes (ABCC4 and ABCC6 as in *An*. *gambiae*). This highlights the potential important role played by ABC transporters in the resistance to insecticides in general as reported recently (Dermauw & Van Leeuwen, 2014; Pignatelli et al., 2018) and particularly in *An*. *funestus*. Results should be interpreted with caution however as comparing resistant mosquitoes of each region with FANG (R‐S) may reflect resistance or underlying differences in population structure. Despite this potentially confounding factor, we consider the significant signal at *CYP9K1* between Uganda resistant and FANG as a signature of resistance. This is because country‐specific PoolSeq results and RNAseq that show a high overexpression of this gene only in Uganda (Weedall et al., 2019), and our results provide further support for the likely key role that this P450 gene plays in the pyrethroid resistance in this country (Weedall et al., 2020). *CYP9K1* has also been implicated in pyrethroid resistant in other mosquito species such as *An*. *parensis* (Mulamba, Irving, et al., 2014) and *An*. *coluzzii* (Vontas et al., 2018). This correlation between RNAseq and targeted sequencing for *CYP9K1* shows that if the phenotypic segregation is wide enough then target enrichment and sequencing could be sufficiently robust to detect variants associated with resistance. Nevertheless, despite narrowing the genomic region associated with resistance to the gene level confirmation of the causative variant requires further fine‐scale sequencing of candidate gene and regulatory regions in tandem with functional genomic dissection of promoter activity. Without taking such an approach whole genome studies do not yield the variants needed to design simple molecular diagnostic for resistance tracking of metabolic resistance. An approach we have taken for other metabolic resistance‐conferring loci: *GSTe2* (Riveron, Yunta, et al., 2014), *CYP6P9a* (Weedall et al., 2019) and *CYP6P9b* (Mugenzi et al., 2019).

### 4.3 *An. funestus* CYP9K1 is a metaboliser of type II pyrethroids

The heterologous expression of *An*. *funestus* CYP9K1 *(AfCYP9K1)* in *E*. *coli* followed by metabolism assays revealed that CYP9K1 metabolises the type II pyrethroid, deltamethrin. Recombinant CYP9K1 had a depletion rate similar to those observed for other cytochrome P450s genes in *An*. *funestus* including CYP6P9b (Riveron et al., 2013), CYP6P9a and CYP6M7 (Riveron, Ibrahim, et al., 2014), CYP9J11 (CYP9J5) (Riveron et al., 2017) and CYP6AA1 (Ibrahim et al., 2018) or in other malaria vectors such as CYP6M2 in *An*. *gambiae* (Stevenson et al., 2011) or CYP6P3 (Muller et al., 2008). However, the observed *An*. *funestus* CYP9K1 depletion rate of deltamethrin was twice that for *An*. *coluzzii* CYP9K1 (64% vs. 32%), shown to be conferring pyrethroid resistance in the *An*. *coluzzii* population of Bioko Island (Vontas et al., 2018) after scale‐up of both LLINs and IRS (Vontas et al., 2018). Notably, *Af*CYP9K1 did not metabolise the type I pyrethroid permethrin, with no substrate depletion observed after 90 min suggesting that *Af*CYP9K1 metabolism is specific to type II pyrethroid. This is similar to previous observations where some P450s could only metabolise one type of pyrethroids. Notably, the CYP6P4 of the malaria vector *An*. *arabiensis* sampled from Chad was shown not to metabolise type II pyrethroid, deltamethrin, which correlated with susceptibility to this insecticide in this mosquito population (Ibrahim et al., 2016). However, we cannot rule out that *Af*CYP9K1 also contributes to type I resistance either through metabolism of secondary metabolites generated by other P450s such as CYP6P9a/b or CYP6P5 also shown to be over‐expressed in Uganda (Riveron et al., 2017; Weedall et al., 2019). *Af*CYP9K1 could also act through other mechanisms such as sequestration. For example, the *An*. *gambiae* CYP6Z2 (AGAP008218) known to metabolize carbaryl (Chiu et al., 2008), insects juvenile hormone analogue insecticide pyriproxyfen (Yunta et al., 2016) and mitochondrial complex I inhibitors, fenazaquin, pyridaben and tolfenpyrad (Lees et al., 2020), does not metabolize permethrin directly but plays a crucial role in the clearance of pyrethroid insecticides via further catabolism of pyrethroid derivatives (3‐phenoxybenzyl aldehyde and 3‐phenoxybenzyl alcohol) obtained by the action of carboxylesterases (Chandor‐Proust et al., 2013), explaining why CYP6Z2 is often found as one of the top overexpressed P450 in permethrin resistant populations of *An*. *gambiae*/*An*. *coluzzii*. Considering the very strong selection on this allele established here and previously (Weedall et al., 2020) further studies are needed to establish the extent, if any, of the interaction of CYP9K1 with type I pyrethroids. One possibility is trans‐regulation of CYP9K1 as reported for the lepidopteron pest, *Spodoptera exigua* for which trans‐acting transcriptional regulators (CncC/Maf) and a *cis*‐regulatory element (Knirps) are both interacting with the 5′ UTR of the P450 gene *CYP321A8*, leading to its upregulation of expression (Hu et al., 2021).

### 4.4 A directionally selected *CYP9K1* allele is driving resistance in Uganda

*CYP9K1* is under strong directional selection in Uganda as shown by the polymorphism pattern of this gene in Uganda, with both low numbers of substitutions (35 vs. 123 in Cameroon) and haplotypes (5 vs. 38 in Cameroon) identified. Strong selection on the *CYP9K1* allele in Uganda is probably driven by the scale up of pyrethroid‐based interventions, notably the mass distribution of bed nets. Scale up of bed nets has been strongly associated with the escalation of pyrethroid resistance in southern African *An*. *funestus* populations (Barnes, Weedall, et al., 2017; Riveron et al., 2019; Weedall et al., 2020).

Furthermore, a single haplotype is predominant for *CYP9K1* in Uganda in line with directional selection. Fixation of strongly directionally selected alleles was also observed in the *CYP6P9a*/*b* P450 genes in *An*. *funestus* from southern African populations (Riveron et al., 2013, 2019; Weedall et al., 2019). This is also the case for *CYP9K1* in *An*. *coluzzii* in Mali (Main et al., 2015) where an allele has been positively selected in populations post‐2006. Similar selective sweeps on P450s have been also reported in *Drosophila melanogaster*, where a single *CYP6G1* allele conferring DDT resistance containing a partial Accord transposable element in the 5’ UTR has spread worldwide (Daborn et al., 2002; Schlenke & Begun, 2004). Previous analysis has also shown that the high selection of *CYP9K1* occurs alongside a high level of overexpression related to duplication of the locus of this gene in Uganda (Weedall et al., 2020). Further supporting selection of an allele with enhanced metabolic efficiency in breaking down pyrethroids. This is supported by the fixation of the amino acid substitution of glycine for alanine at position 454 (G454A). This position is located close to the substrate binding pocket, and we hypothesise that increase the affinity and metabolism of this enzyme for deltamethrin. A similar scenario was seen for *An*. *funestus CYP6P9a*/*b* for which both in vivo and in vitro studies revealed that key amino acid changes (N384S) were able to increase the catalytic efficiency of these enzymes (Ibrahim et al., 2015). Further evidence comes from humans for which amino acid changes in *CYP2D6*,*CYP2C9*,*CYP2C19* and *CYP2A6* have been shown to affect drug metabolism a low drug metabolism conferred by some alleles while others confer a fast metabolism rate (Ingelman‐Sundberg et al., 2007). Similarly, other amino acid changes in the glutathione S‐transferase *GSTe2* enzyme in *An*. *funestus* (L119F) (Riveron, Yunta, et al., 2014) and in *An*. *gambiae* (I114T) (Mitchell et al., 2014) were also shown to drive pyrethroid/DDT resistance in these vectors.

By integrating the PoolSeq‐based GWAS and deep target sequencing of pyrethroid resistant and putatively susceptible mosquitoes with in vitro functional validation in *E*. *coli* of identified candidate genes. We have demonstrated that *CYP9K1* is driving pyrethroid resistance in Eastern African populations of *An*. *funestus*. This result improves our understanding of the molecular basis of metabolic resistance to pyrethroid in malaria vectors and will inform the design of diagnostic tool to detect and track this resistance across Africa.

## AUTHOR CONTRIBUTIONS

Charles S. Wondji conceived and designed the study, Jacob M. Riveron and Charles S. Wondji collected the mosquito field samples. Helen Irving, Jacob M. Riveron and Gareth D. Weedall prepared all samples for genomic sequencing. Gareth D. Weedall, Charles S. Wondji and Jack Hearn analysed pooled‐template genomic data. Charles S. Wondji designed the SureSelect baits and analyse the sequencing data. Sulaiman S. Ibrahim performed the *CYP9K1* metabolism assay and sequence characterisation of *CYP9K1*; Leon M. J. Mugenzi, Billy Tene‐Fossog and Carlos S. Djoko Tagne analysed the *CYP9K1* polymorphism; Jack Hearn and Charles S. Wondji wrote the manuscript. All authors read and approved the final manuscript.

## CONFLICT OF INTEREST

The authors declare that they have no competing interests.

### OPEN RESEARCH BADGES

This article has earned an Open Data Badge for making publicly available the digitally‐shareable data necessary to reproduce the reported results. Pooled template whole genome sequencing data are available under study accessions PRJEB24379 (Cameroon and Malawi PoolSeq), PRJEB24520 (Cameroon SureSelect), PRJEB47287 (Malawi and Uganda SureSelect) and PRJEB24506 (FANG SureSelect).

## Supporting information

Figure S1‐S6

Table S1‐S16

## Data Availability

All genomic data sets are available from the European Nucleotide Archive. Pooled template whole genome sequencing data are available under study accessions PRJEB24379 (Cameroon and Malawi PoolSeq), PRJEB24520 (Cameroon SureSelect), PRJEB47287 (Malawi and Uganda SureSelect [Release date 1 December 2021]) and PRJEB24506 (FANG SureSelect) (Hearn et al., 2021).
