# Supplementary material for: Multi‐omics analysis identifies a CYP9K1 haplotype conferring pyrethroid resistance in the malaria vector Anopheles funestus in East Africa
Source: Mol Ecol. 2022 May 24;31(13):3642–57. doi: 10.1111/mec.16497 (PMC9321817; doi:10.1111/mec.16497)
Supplement: Supplementary file 1 — Figure S1‐S6 [file MEC-31-3642-s002.docx]

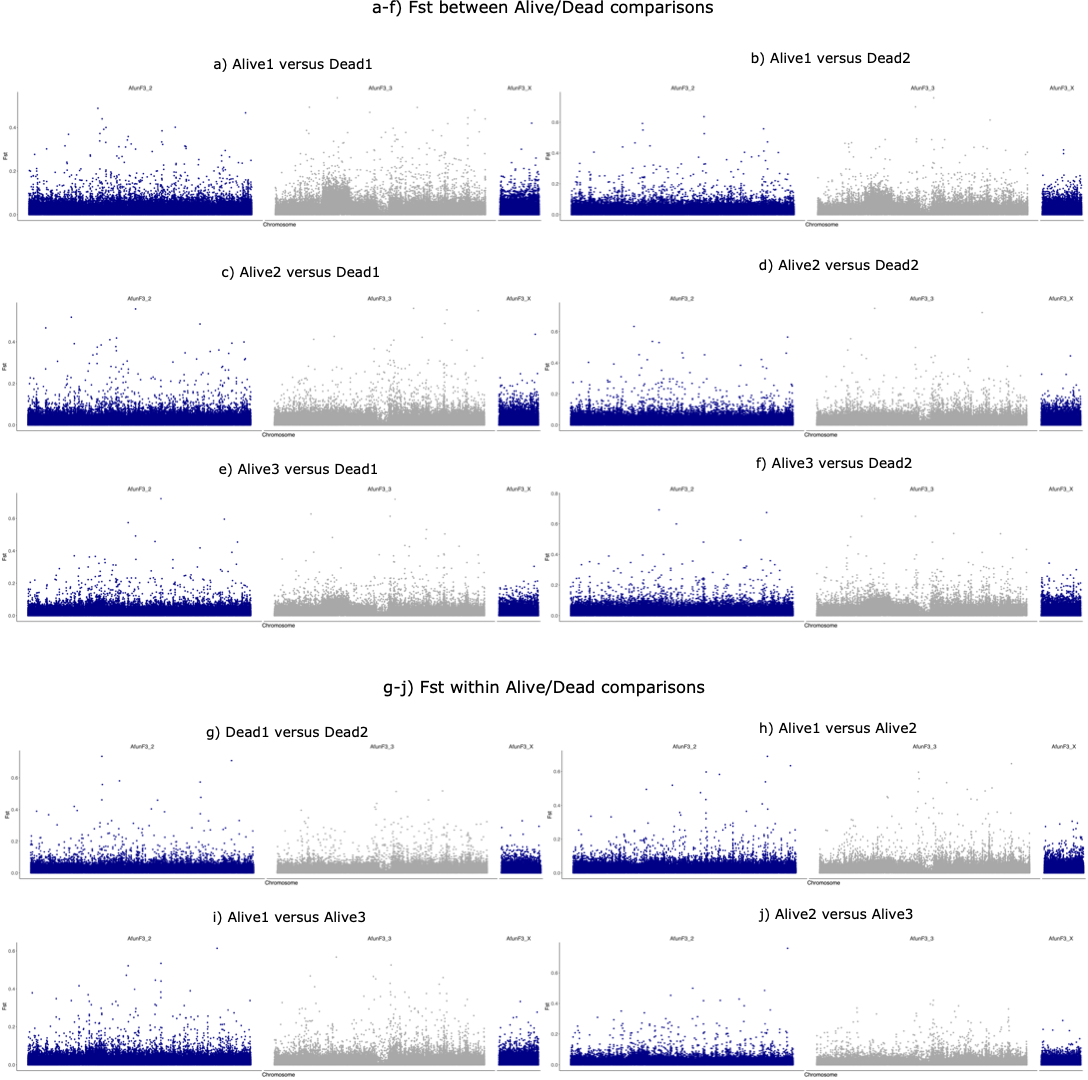


**Figure S1: Pairwise F_st_ between all Malawi replicates from 1000 bp non-overlapping sliding windows.** a-f) All combinations of resistant (Alive) versus susceptible (Dead) mosquitoes and g-j) resistant versus resistant and susceptible versus susceptible comparisons.


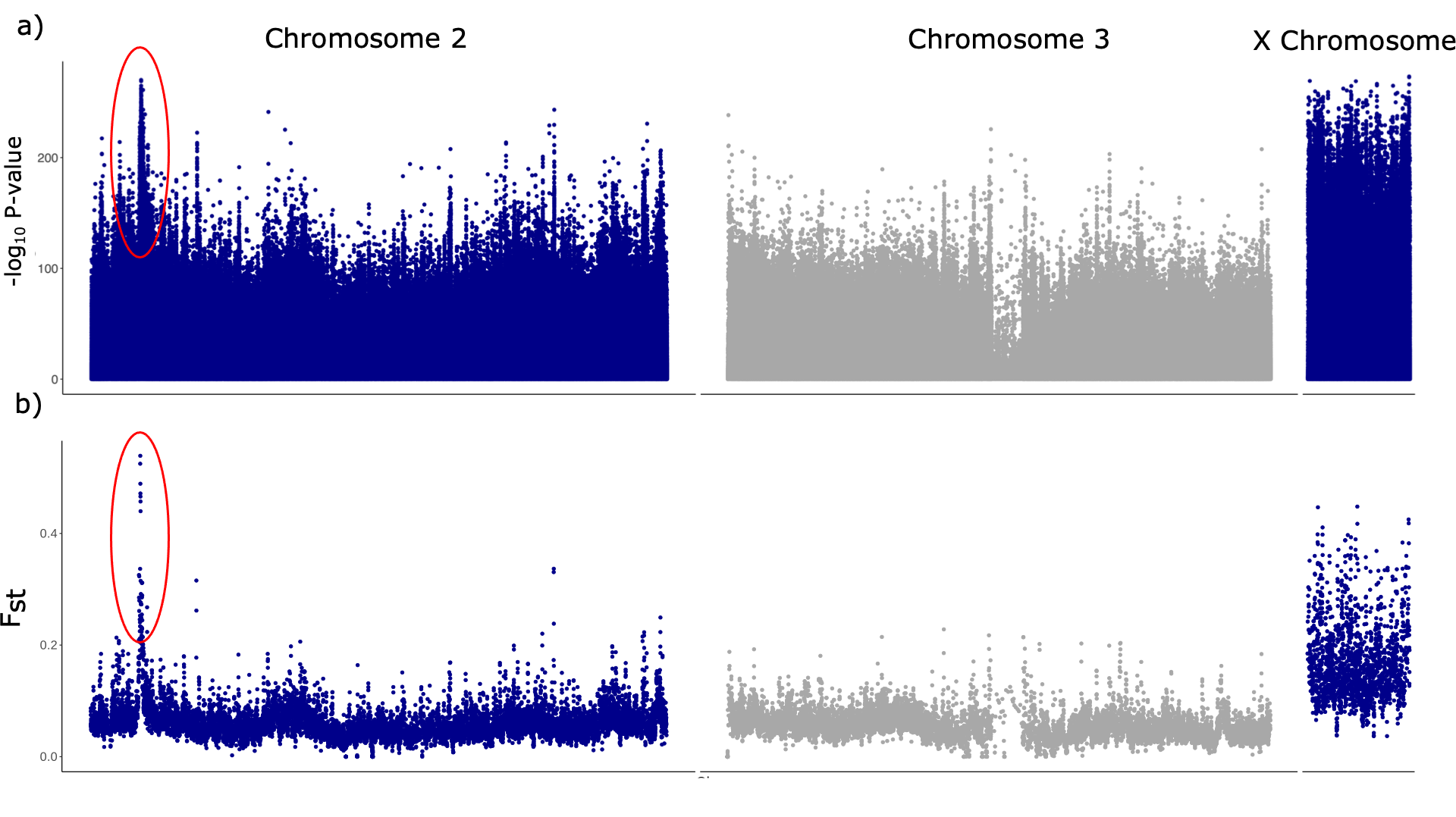


**Figure S2**. **PoolSeq GWAS of all Malawi and Cameroon replicates.** a) Cochran- Mantel-Haenszel test –log10 P-values per SNP calculated in Popoolation 2, b) Fst values for 1000 bp windows calculated in poolfstat. SNPs overlapping the *rp1* resistance locus containing the *CYP6P9a/b* cytochrome P450 genes are circled in red.


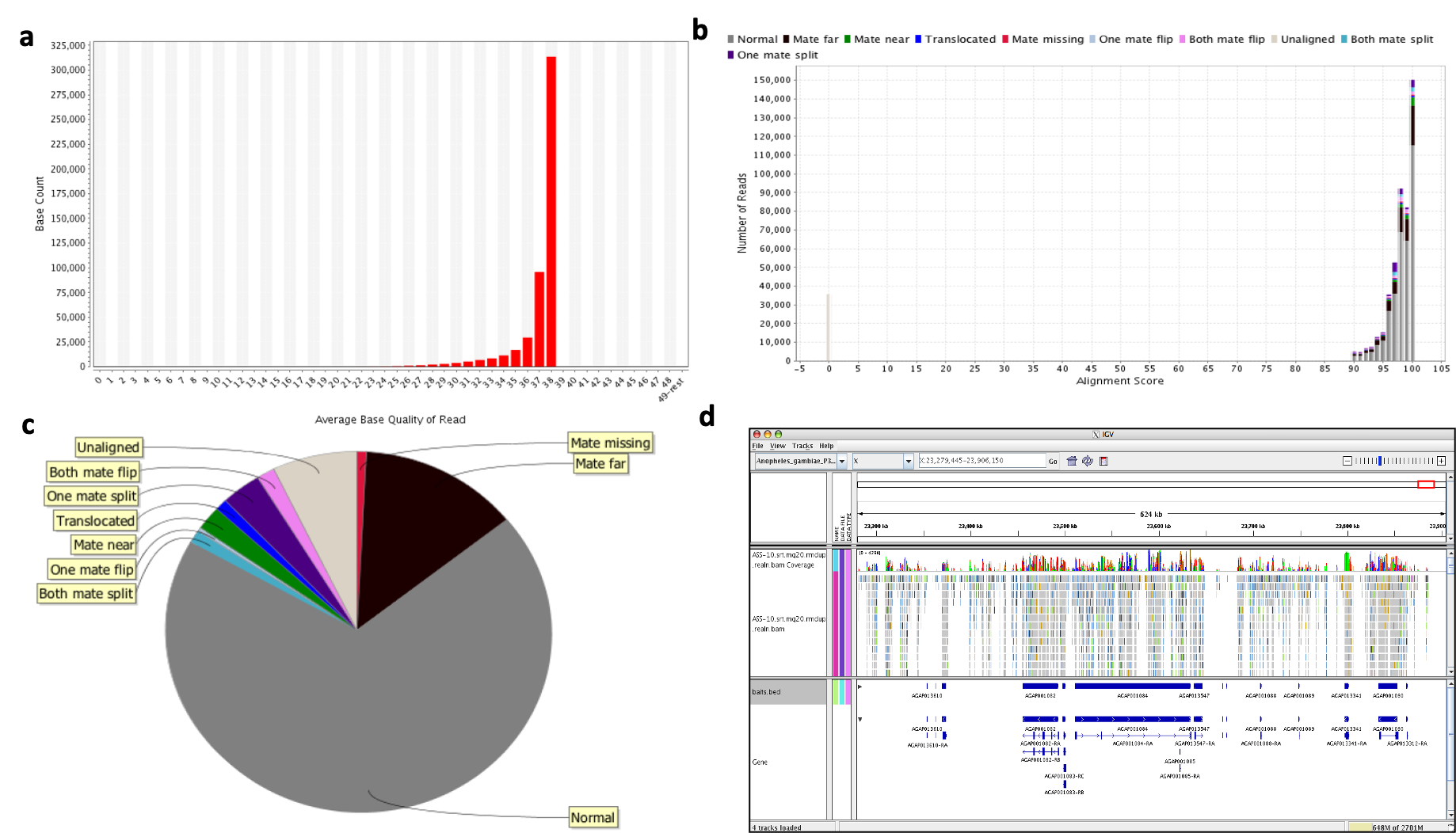


**Figure S3: Quality control of targeted sequencing:** (A) Average base quality of the reads for one mosquito from Malawi showing the distribution of the base quality score across bases of all reads. (B) alignment score of the mapped reads showing the distribution of reads based on their alignment scores. (C) Pie-chart displaying the match status of paired ended reads. This represents the proportion of reads with different read statuses for paired data. (D) The IGV screen showing an overview of the coverage of the some of the targeted genomic regions after SureSelect target enrichment and sequencing.


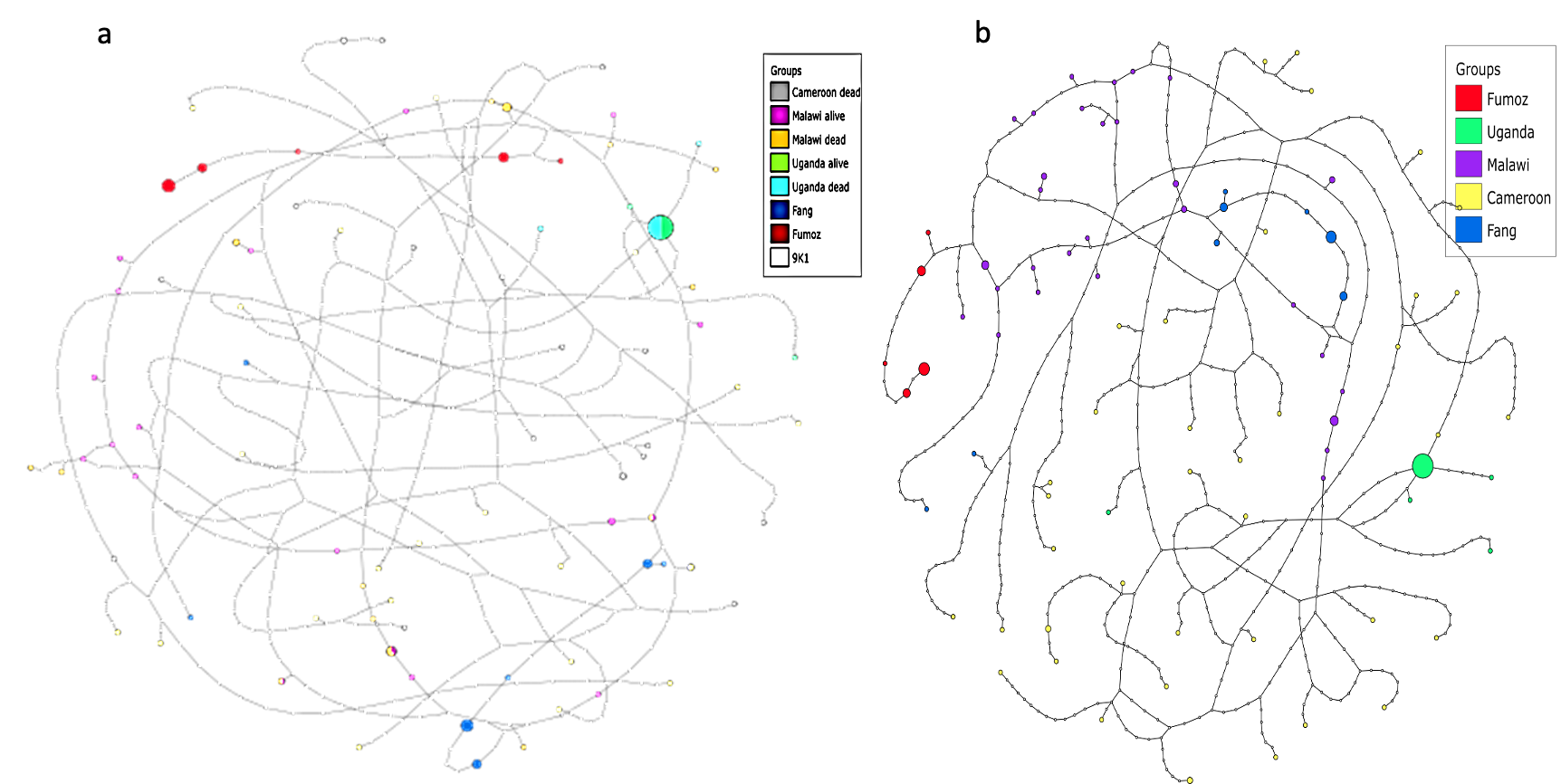


**Figure S4:** Haplotype network for *CYP9K1* full sequence (2707bp) using sure select data. a. Alive and dead per population. b. Pooled alive and dead per population.


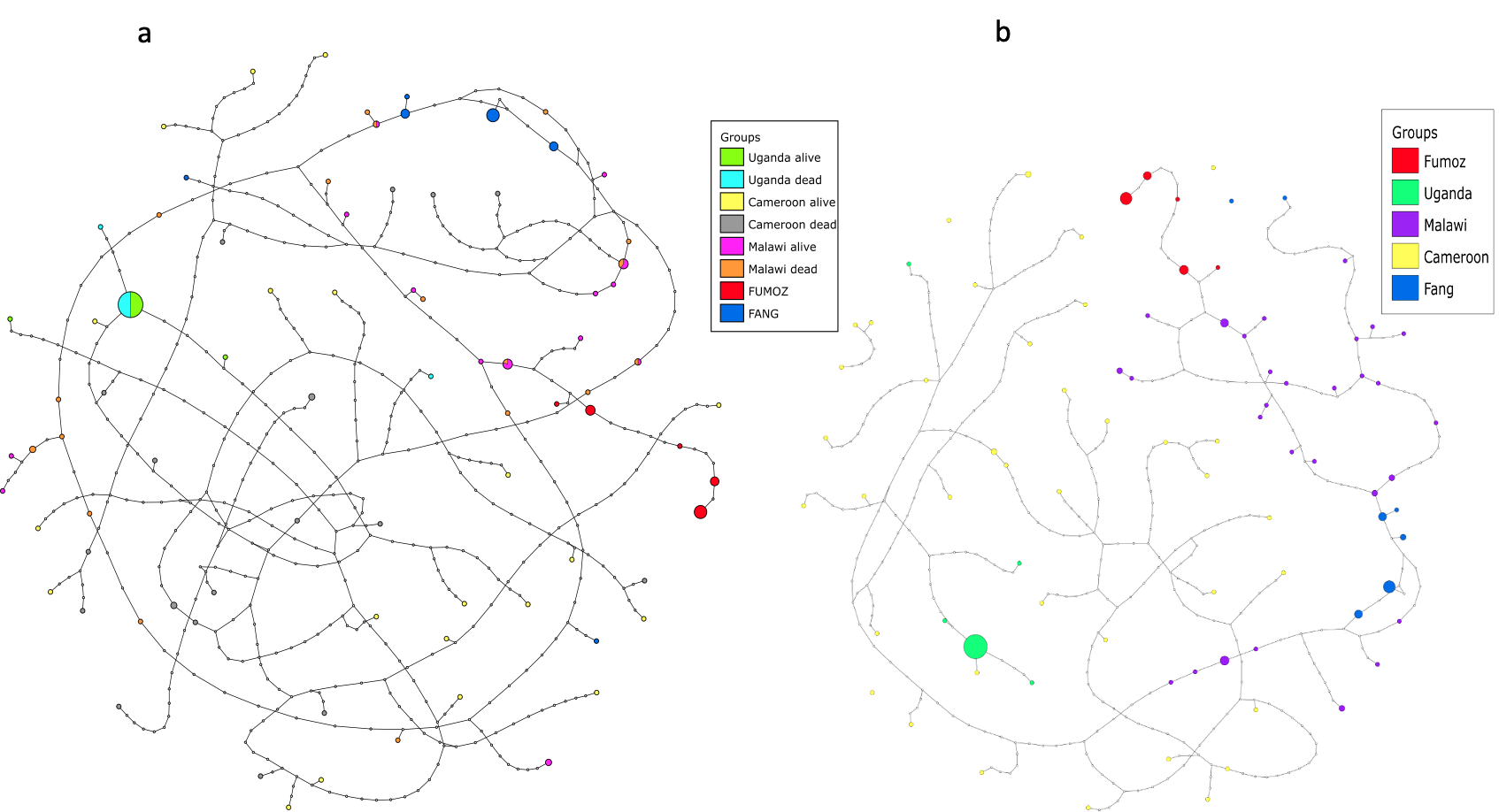


**Figure S5:** Haplotype networks for *CYP9K1* coding sequence (1614bp) using sure select data; a. Alive and dead per population. b. Pooled alive and dead per population.


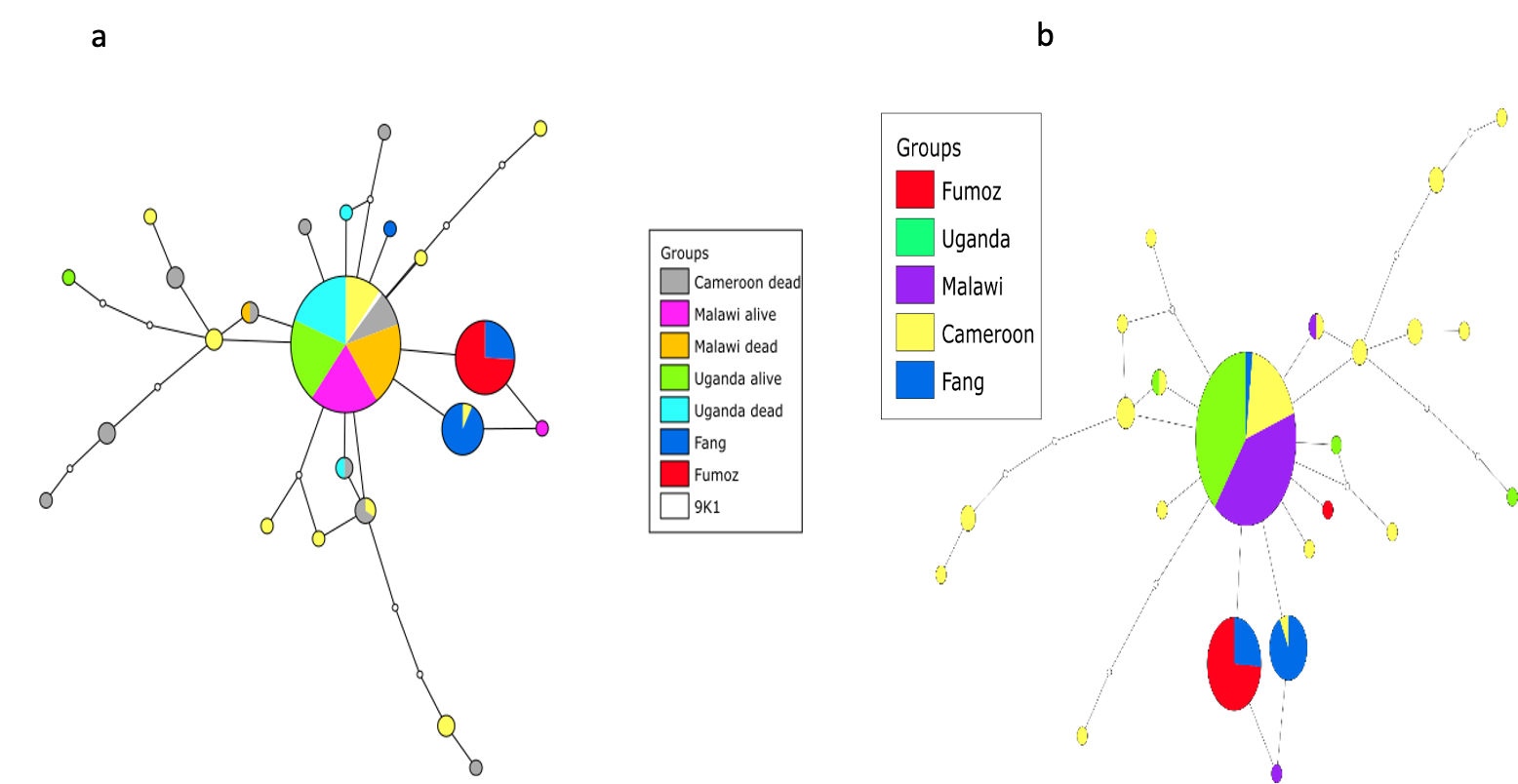


**Figure S6:** Haplotype networks for *CYP9K1* noncoding sequence (1093bp) using SureSelect data; a. Alive and dead per population. b. Pooled alive and dead per population.
