# Supplementary material for: Multi‐omics analysis identifies a CYP9K1 haplotype conferring pyrethroid resistance in the malaria vector Anopheles funestus in East Africa
Source: Mol Ecol. 2022 May 24;31(13):3642–57. doi: 10.1111/mec.16497 (PMC9321817; doi:10.1111/mec.16497)
Supplement: Supplementary file 2 — Table S1‐S16 [file MEC-31-3642-s001.docx]

**Table S1**: Primers used for the cloning of CYP9K1 for the heterologous expression in *E. coli.*

| **Primer Name** | **Forward** | **Reverse** |
| --- | --- | --- |
| AfunCYP9K1_Full | ATGCTGGGTACGCTCGTTGC | TCAGCAAGATTCTAGATCTAG |
| OMPA+2F | GGAATTCCATATGAAAAAGACAGCTATCGCG |  |
| OMPA+2CYP9K1_F | GCAACGAGCGTACCCAGCATCGGAGCGGCCTGCGCTACGGTAGCGAA |  |
| CYP9K1_R |  | TCTAGAGAATTCTCACGCAAACGGAACCGTATCCA |

Restriction sites are in different colours and underlined: Yellow is *EcoR*I, Purple is *Nde*I, Red is *Xba*I.

**Table S2.** Descriptive statistics of PoolSeq sequence read data and alignments of permethrin-resistant and susceptible mosquitoes from Malawi and Cameroon.

| **Sample name** | **MWI-PER-060-DEAD-A** | **MWI-PER-060-DEAD-B** | **MWI-PER-180-ALIVE-A** | **MWI-PER-180-ALIVE-B** | **MWI-PER-180-ALIVE-C** | **CMR-PER-020-DEAD** | **CMR-PER-060-ALIVE** |
| --- | --- | --- | --- | --- | --- | --- | --- |
| **Trimmed reads^1^** | 122,052,496 | 110,235,831 | 96,357,917 | 104,715,851 | 101,472,749 | 208,069,688 | 161,093,419 |
| **Aligned reads (%)** | 113,477,040 (92.97%) | 102,481,811 (92.97%) | 90,065,741 (93.47%) | 97,854,501 (93.45%) | 95,144,827 (93.76%) | 186,031,542 (89.41%) | 148,333,136 (92.08%) |
| **Properly paired (%)^2^** | 106,189,466 (88.20%) | 95,695,664 (88.08%) | 84,256,324 (88.60%) | 91,463,700 (88.53%) | 88,925,276 (88.86%) | 173,277,786 (84.55%) | 138,279,570 (87.41%) |
| **Singleton (%)** | 599,430 (0.50%) | 549,250 (0.51%) | 465,858 (0.49%) | 514,278 (0.50%) | 477,228 (0.48%) | 1,124,354 (0.55%) | 791,103 (0.50%) |

^1^ Forward (R1) and reverse (R2) read pairs after trimming

^2^ Properly paired means both read and its mate are mapped to opposing strands of the reference sequence, with 3’ ends innermost and 5’ ends within the allowed distance from each other (0-500 bp)

**Table S3**: Descriptive statistics of SureSelect sequence read data for FANG colony mosquitoes and permethrin-resistant and susceptible mosquitoes from Cameroon, Uganda and Malawi.

| **Sample name** | **Untrimmed reads** | **Trimmed reads** | **R1/R2 pairs ^1^** | **R0 reads (%) ^2^** |
| --- | --- | --- | --- | --- |
| Sample_9-Fang-03 | 1,028,454 | 1,024,825 | 510,613 | 3,599 (0.35%) |
| Sample_10-Fang-05 | 1,114,978 | 1,110,233 | 552,757 | 4,719 (0.43%) |
| Sample_11-Fang-08 | 994,192 | 989,572 | 492,495 | 4,582 (0.46%) |
| Sample_12-Fang-11 | 1,115,700 | 1,111,463 | 553,629 | 4,205 (0.38%) |
| Sample_13-Fang-19 | 1,083,448 | 1,079,139 | 537,431 | 4,277 (0.40%) |
| Sample_14-Fang-22 | 1,141,902 | 1,137,236 | 566,325 | 4,586 (0.40%) |
| Sample_15-Fang-24 | 972,222 | 967,399 | 481,311 | 4,777 (0.49%) |
| Sample_16-Fang-26 | 1,134,266 | 1,128,799 | 561,691 | 5,417 (0.48%) |
| Sample_17-Fang-28 | 954,604 | 950,674 | 473,404 | 3,866 (0.41%) |
| Sample_18-Fang-31 | 1,006,792 | 1,002,749 | 499,365 | 4,019 (0.40%) |
| Sample_1-CMR-PER-AL-10 | 1,497,866 | 1,482,964 | 734,095 | 14,774 (1.00%) |
| Sample_2-CMR-PER-AL-13 | 1,344,226 | 1,331,154 | 659,098 | 12,958 (0.97%) |
| Sample_3-CMR-PER-AL-24 | 1,539,562 | 1,518,385 | 748,679 | 21,027 (1.38%) |
| Sample_4-CMR-PER-AL-42 | 1,428,794 | 1,411,934 | 697,600 | 16,734 (1.19%) |
| Sample_5-CMR-PER-AL-45 | 1,521,998 | 1,506,775 | 745,831 | 15,113 (1.00%) |
| Sample_6-CMR-PER-AL-46 | 1,413,534 | 1,395,338 | 688,628 | 18,082 (1.30%) |
| Sample_7-CMR-PER-AL-47 | 1,350,674 | 1,337,203 | 661,923 | 13,357 (1.00%) |
| Sample_8-CMR-PER-AL-52 | 1,548,800 | 1,529,842 | 755,511 | 18,820 (1.23%) |
| Sample_9-CMR-PER-AL-53 | 1,574,588 | 1,557,785 | 770,551 | 16,683 (1.07%) |
| Sample_10-CMR-PER-AL-54 | 1,698,838 | 1,673,521 | 824,500 | 24,521 (1.47%) |
| Sample_11-CMR-PER-DE-15 | 1,510,686 | 1,490,650 | 735,365 | 19,920 (1.34%) |
| Sample_12-CMR-PER-DE-16 | 1,184,730 | 1,170,707 | 578,401 | 13,905 (1.19%) |
| Sample_13-CMR-PER-DE-24 | 1,495,458 | 1,478,602 | 730,941 | 16,720 (1.13%) |
| Sample_14-CMR-PER-DE-26 | 1,434,488 | 1,414,092 | 696,925 | 20,242 (1.43%) |
| Sample_15-CMR-PER-DE-27 | 1,644,246 | 1,622,403 | 800,357 | 21,689 (1.34%) |
| Sample_16-CMR-PER-DE-31 | 1,108,940 | 1,077,688 | 523,294 | 31,100 (2.89%) |
| Sample_17-CMR-PER-DE-33 | 1,516,380 | 1,497,969 | 740,429 | 17,111 (1.14%) |
| Sample_18-CMR-PER-DE-34 | 1,557,076 | 1,527,720 | 749,270 | 29,180 (1.91%) |
| Sample_19-CMR-PER-DE-35 | 1,597,194 | 1,562,608 | 764,118 | 34,372 (2.20%) |
| Sample_20-CMR-PER-DE-40 | 1,588,264 | 1,568,527 | 774,469 | 19,589 (1.25%) |
| Sample_1-UG-120-AL-14 | 1,260,862 | 1,256,465 | 626,065 | 4,335 (0.35%) |
| Sample_2-UG-120-AL-18 | 1,323,658 | 1,318,233 | 656,434 | 5,365 (0.41%) |
| Sample_3-UG-120-AL-19 | 1,301,976 | 1,296,717 | 645,752 | 5,213 (0.40%) |
| Sample_4-UG-120-AL-20 | 1,263,302 | 1,257,948 | 626,330 | 5,288 (0.42%) |
| Sample_5-UG-120-AL-25 | 1,271,058 | 1,266,716 | 631,268 | 4,180 (0.33%) |
| Sample_6-UG-120-AL-26 | 1,317,308 | 1,310,559 | 652,137 | 6,285 (0.48%) |
| Sample_7-UG-120-AL-27 | 1,283,886 | 1,278,943 | 637,169 | 4,605 (0.36%) |
| Sample_8-UG-120-AL-28 | 1,306,298 | 1,302,548 | 649,426 | 3,696 (0.28%) |
| Sample_9-UG-120-AL-29 | 1,400,044 | 1,394,249 | 694,268 | 5,713 (0.41%) |
| Sample_10-UG-120-AL-30 | 1,150,148 | 1,145,348 | 570,304 | 4,740 (0.41%) |
| Sample_11-UG-45-DE-13 | 1,346,264 | 1,340,659 | 667,559 | 5,541 (0.41%) |
| Sample_12-UG-45-DE-14 | 1,389,134 | 1,381,958 | 687,426 | 7,106 (0.51%) |
| Sample_13-UG-45-DE-15 | 1,289,678 | 1,284,193 | 639,479 | 5,235 (0.41%) |
| Sample_14-UG-45-DE-16 | 1,251,840 | 1,245,947 | 620,061 | 5,825 (0.47%) |
| Sample_15-UG-45-DE-17 | 1,511,496 | 1,504,410 | 748,698 | 7,014 (0.47%) |
| Sample_16-UG-45-DE-18 | 1,265,474 | 1,259,715 | 627,016 | 5,683 (0.45%) |
| Sample_17-UG-60-DE-19 | 1,260,866 | 1,255,900 | 625,502 | 4,896 (0.39%) |
| Sample_18-UG-60-DE-20 | 989,348 | 984,688 | 490,046 | 4,596 (0.47%) |
| Sample_19-UG-60-DE-25 | 1,399,058 | 1,393,304 | 693,829 | 5,646 (0.41%) |
| Sample_20-UG-60-DE-30 | 1,463,342 | 1,457,897 | 726,271 | 5,355 (0.37%) |
| Sample_1-MAL_PERM_60_DE | 1,573,144 | 1,567,555 | 781,025 | 5,505 (0.35%) |
| Sample_2-MAL_PERM_60_DE | 1,608,850 | 1,602,193 | 797,798 | 6,597 (0.41%) |
| Sample_3-MAL_PERM_60_DE | 1,612,528 | 1,607,271 | 801,030 | 5,211 (0.32%) |
| Sample_4-MAL_PERM_60_DE | 1,202,974 | 1,198,847 | 597,376 | 4,095 (0.34%) |
| Sample_5-MAL_PERM_60_DE | 1,726,920 | 1,720,793 | 857,369 | 6,055 (0.35%) |
| Sample_6-MAL_PERM_60_DE | 1,598,412 | 1,591,718 | 792,546 | 6,626 (0.42%) |
| Sample_7-MAL_PERM_60_DE | 1,497,204 | 1,491,881 | 743,312 | 5,257 (0.35%) |
| Sample_8-MAL_PERM_60_DE | 1,324,632 | 1,316,582 | 654,320 | 7,942 (0.60%) |
| Sample_9-MAL_PERM_60_DE | 1,464,702 | 1,459,073 | 726,755 | 5,563 (0.38%) |
| Sample_10-MAL_PERM_60_DE | 1,747,970 | 1,740,208 | 866,265 | 7,678 (0.44%) |
| Sample_11-MAL_PERM_180_AL | 1,840,610 | 1,832,479 | 912,219 | 8,041 (0.44%) |
| Sample_12-MAL_PERM_180_AL | 1,764,674 | 1,757,863 | 875,576 | 6,711 (0.38%) |
| Sample_13-MAL_PERM_180_AL | 1,560,370 | 1,554,141 | 773,989 | 6,163 (0.40%) |
| Sample_14-MAL_PERM_180_AL | 1,560,018 | 1,555,076 | 775,090 | 4,896 (0.31%) |
| Sample_15-MAL_PERM_180_AL | 1,675,688 | 1,670,342 | 832,541 | 5,260 (0.31%) |
| Sample_16-MAL_PERM_180_AL | 1,539,418 | 1,534,723 | 765,042 | 4,639 (0.30%) |
| Sample_17-MAL_PERM_180_AL | 1,533,600 | 1,528,381 | 761,615 | 5,151 (0.34%) |
| Sample_18-MAL_PERM_180_AL | 1,530,638 | 1,524,101 | 758,828 | 6,445 (0.42%) |
| Sample_19-MAL_PERM_180_AL | 1,612,018 | 1,606,432 | 800,462 | 5,508 (0.34%) |
| Sample_20-MAL_PERM_180_AL | 1,452,988 | 1,447,223 | 720,768 | 5,687 (0.39%) |

^1^ Forward (R1) and reverse (R2) read pairs after trimming

^2^ Reads unpaired after trimming (% of total trimmed reads)

**Table S4:** Mapping metrics of the targeted sequencing relative to the reference genome.

|  | **Total reads** | **Uniquely mapped reads** | **Avg read length(Reads in targeted regions only)** | **Reads in targeted regions** | **Reads in targeted regions (%)** | **Reads in targeted regions +/100bp(#)** | **Total bases of reads (includes bases of unmatched reads)** | **Uniquely Aligned Bases (%)** | **Bases of reads within targeted regions** | **Bases of reads within targeted regions (%)** |
| --- | --- | --- | --- | --- | --- | --- | --- | --- | --- | --- |
| 1-CMRPER-AL10 | 1,466,035 | 674,878 | 144.85 | 669,071 | 99.14 | 674,878 | 212,058,508 | 45.88 | 95,525,462 | 98.18 |
| 2-CMRPER-AL-13 | 1,316,376 | 612,722 | 144.52 | 607,474 | 99.14 | 612,722 | 190,053,176 | 46.38 | 86,534,865 | 98.18 |
| 3-CMRPER-AL24 | 1,494,476 | 707,001 | 144.84 | 701,227 | 99.18 | 707,001 | 216,033,880 | 47.19 | 100,195,452 | 98.29 |
| 4-CMRPER-AL-42 | 1,392,900 | 700,691 | 143.74 | 696,052 | 99.34 | 700,691 | 199,903,027 | 50.2 | 98,914,305 | 98.57 |
| 5-CMRPER-AL-45 | 1,489,659 | 735,151 | 144.78 | 729,286 | 99.2 | 735,151 | 215,344,383 | 49.21 | 104,156,110 | 98.29 |
| 6-CMRPER-AL-46 | 1,374,737 | 677,169 | 144.65 | 671,963 | 99.23 | 677,169 | 198,485,659 | 49.14 | 95,980,212 | 98.4 |
| 7-CMRPER-AL-47 | 1,321,974 | 601,591 | 145.15 | 596,389 | 99.14 | 601,591 | 191,671,109 | 45.34 | 85,298,688 | 98.15 |
| 8-CMR-PER-AL-52 | 1,508,544 | 740,094 | 144.6 | 734,743 | 99.28 | 740,094 | 217,794,186 | 48.94 | 104,886,561 | 98.4 |
| 9-CMR-PER-AL-53 | 1,538,756 | 735,307 | 145.35 | 729,130 | 99.16 | 735,307 | 223,121,542 | 47.68 | 104,445,463 | 98.18 |
| 10-CMR-AL-54 | 1,645,682 | 780,310 | 144.98 | 773,416 | 99.12 | 780,310 | 237,818,738 | 47.34 | 110,489,380 | 98.14 |
| 11-CMR-DE-15 | 1,468,122 | 699,173 | 145.36 | 693,317 | 99.16 | 699,173 | 212,870,775 | 47.52 | 99,338,543 | 98.2 |
| 12-CMR-PER-DE-16 | 1,154,803 | 541,328 | 145.16 | 536,365 | 99.08 | 541,328 | 167,225,798 | 46.75 | 76,649,359 | 98.04 |
| 13-CMR-PER-DE-24 | 1,459,624 | 719,468 | 145.22 | 713,124 | 99.12 | 719,468 | 211,435,147 | 49.17 | 102,037,646 | 98.14 |
| 14-CMR-PER-DE-26 | 1,391,034 | 648,806 | 145.05 | 643,422 | 99.17 | 648,806 | 201,139,392 | 46.58 | 91,981,226 | 98.18 |
| 15-CMR-PER-DE-27 | 1,597,838 | 743,207 | 144.92 | 736,514 | 99.1 | 743,207 | 230,998,866 | 46.39 | 105,117,347 | 98.08 |
| 16-CMRPER-DE-31 | 1,042,370 | 492,850 | 143.23 | 489,451 | 99.31 | 492,850 | 148,380,850 | 47.39 | 69,345,403 | 98.61 |
| 17-CMRPER-DE-33 | 1,478,389 | 678,529 | 145.42 | 672,831 | 99.16 | 678,529 | 214,364,726 | 45.82 | 96,390,537 | 98.14 |
| 18-CMRPER-DE-34 | 1,494,456 | 697,192 | 144.74 | 691,514 | 99.19 | 697,192 | 215,475,774 | 46.62 | 98,692,646 | 98.24 |
| 19-CMRPER-DE-35 | 1,523,435 | 726,281 | 144.49 | 720,368 | 99.19 | 726,281 | 218,897,170 | 47.73 | 102,665,765 | 98.27 |
| 20-CMRPER-DE-40 | 1,546,054 | 721,402 | 145.34 | 714,945 | 99.1 | 721,402 | 224,025,330 | 46.57 | 102,333,214 | 98.09 |
| 10-Fang-05 | 1,104,988 | 525,655 | 146.04 | 521,232 | 99.16 | 525,655 | 161,413,660 | 47.34 | 75,067,812 | 98.24 |
| 11-Fang-08 | 984,517 | 472,904 | 145.65 | 469,144 | 99.2 | 472,904 | 143,508,602 | 47.79 | 67,465,084 | 98.38 |
| 12-Fang-11 | 1,106,756 | 523,438 | 145.7 | 519,049 | 99.16 | 523,438 | 161,345,675 | 47.05 | 74,589,201 | 98.26 |
| 13-Fang-19 | 1,074,458 | 518,820 | 145.55 | 514,474 | 99.16 | 518,820 | 156,487,603 | 48.03 | 73,866,012 | 98.27 |
| 14-Fang-22 | 1,132,220 | 536,603 | 146.02 | 532,138 | 99.17 | 536,603 | 165,386,917 | 47.16 | 76,652,638 | 98.28 |
| 15-Fang-24 | 962,122 | 455,645 | 145.66 | 451,791 | 99.15 | 455,645 | 140,184,338 | 47.13 | 64,895,384 | 98.23 |
| 16-Fang-26 | 1,122,846 | 528,929 | 145.69 | 524,532 | 99.17 | 528,929 | 163,614,721 | 46.88 | 75,387,315 | 98.28 |
| 18-Fang-31 | 998,347 | 481,446 | 145.93 | 477,444 | 99.17 | 481,446 | 145,756,053 | 47.98 | 68,716,208 | 98.25 |
| 9-Fang-03 | 1,020,840 | 484,533 | 145.94 | 480,532 | 99.17 | 484,533 | 149,018,273 | 47.24 | 69,164,990 | 98.26 |
| 17-Fang28 | 946,377 | 455,258 | 145.82 | 451,415 | 99.16 | 455,258 | 138,015,671 | 47.87 | 64,896,689 | 98.22 |
| 11-MALPER-180-AL | 495,700 | 242,866 | 144.68 | 240,793 | 99.15 | 242,866 | 71,765,820 | 48.73 | 34,364,274 | 98.26 |
| 12-MALPER-180-AL | 496,456 | 241,774 | 144.61 | 239,566 | 99.09 | 241,774 | 71,844,378 | 48.42 | 34,163,804 | 98.2 |
| 13-MALPER-180-AL | 496,292 | 238,602 | 144.75 | 236,372 | 99.07 | 238,602 | 71,925,924 | 47.77 | 33,724,155 | 98.14 |
| 14-MALPER-180-AL | 497,046 | 238,500 | 145.19 | 236,358 | 99.1 | 238,500 | 72,220,462 | 47.71 | 33,821,997 | 98.16 |
| 15-MALPER-180-AL | 497,028 | 239,635 | 144.57 | 237,503 | 99.11 | 239,635 | 71,912,716 | 47.94 | 33,863,627 | 98.23 |
| 16-MALPER-180-AL | 497,096 | 240,582 | 144.68 | 238,512 | 99.14 | 240,582 | 71,972,652 | 48.13 | 34,017,217 | 98.2 |
| 17-MALPER-180-AL | 496,832 | 244,162 | 145.1 | 242,029 | 99.13 | 244,162 | 72,122,692 | 48.88 | 34,632,504 | 98.23 |
| 18-MALPER-180-AL | 496,131 | 241,764 | 145.17 | 239,748 | 99.17 | 241,764 | 72,065,596 | 48.48 | 34,328,510 | 98.26 |
| 19-MALPER-A180-AL | 496,749 | 242,858 | 144.49 | 240,722 | 99.12 | 242,858 | 71,804,783 | 48.63 | 34,313,105 | 98.26 |
| 20-MALPER-180-AL | 496,171 | 241,376 | 144.84 | 239,212 | 99.1 | 241,376 | 71,929,814 | 48.37 | 34,168,785 | 98.22 |
| 1-MALPER-60-DE | 496,650 | 235,350 | 144.3 | 233,340 | 99.15 | 235,350 | 71,789,195 | 47.08 | 33,236,674 | 98.33 |
| 2-MALPER-60-DE | 496,149 | 237,218 | 144.23 | 235,140 | 99.12 | 237,218 | 71,653,236 | 47.52 | 33,457,294 | 98.25 |
| 3-MALPER-60-DE | 496,930 | 233,267 | 144.77 | 231,369 | 99.19 | 233,267 | 72,039,423 | 46.67 | 33,054,397 | 98.32 |
| 4-MALPER-60-DE | 496,730 | 238,435 | 144.28 | 236,456 | 99.17 | 238,435 | 71,789,676 | 47.7 | 33,667,418 | 98.32 |
| 5-MALPER-60-DE | 496,623 | 240,755 | 144.57 | 238,683 | 99.14 | 240,755 | 71,867,697 | 48.21 | 34,049,999 | 98.29 |
| 6-MALPER-60-DE | 496,043 | 235,561 | 145.33 | 233,760 | 99.24 | 235,561 | 72,226,542 | 47.2 | 33,540,124 | 98.39 |
| 7-MALPER-60-DE | 496,718 | 230,470 | 145.41 | 228,452 | 99.12 | 230,470 | 72,304,472 | 46.12 | 32,744,243 | 98.19 |
| 8-MALPER-60-DE | 494,712 | 238,102 | 144.16 | 236,118 | 99.17 | 238,102 | 71,398,747 | 47.85 | 33,575,249 | 98.27 |
| 9-MALPER-60-DE | 496,441 | 235,960 | 145.23 | 233,906 | 99.13 | 235,960 | 72,172,467 | 47.26 | 33,499,906 | 98.22 |
| 10-MALPER-60-DE | 495,858 | 240,076 | 145.12 | 237,992 | 99.13 | 240,076 | 72,004,816 | 48.15 | 34,066,436 | 98.25 |
| 1-UG-120-AL-14 | 1,251,548 | 609,695 | 146.23 | 604,449 | 99.14 | 609,695 | 182,980,590 | 48.5 | 87,091,420 | 98.14 |
| 2-UG-120-AL-18 | 1,312,340 | 628,843 | 146.5 | 623,651 | 99.17 | 628,843 | 192,223,402 | 47.71 | 90,034,423 | 98.18 |
| 3-UG-120-AL-19 | 1,290,850 | 611,379 | 146.37 | 606,063 | 99.13 | 611,379 | 188,877,466 | 47.15 | 87,394,082 | 98.13 |
| 4-UG-120-AL-20 | 1,252,062 | 602,275 | 146.05 | 597,254 | 99.17 | 602,275 | 182,886,725 | 47.88 | 85,989,252 | 98.21 |
| 5-UG-120-AL-25 | 1,262,047 | 593,208 | 146.55 | 587,998 | 99.12 | 593,208 | 184,826,709 | 46.81 | 84,860,909 | 98.09 |
| 6-UG-120-AL-26 | 1,303,435 | 615,873 | 145.58 | 610,411 | 99.11 | 615,873 | 189,606,544 | 47.05 | 87,558,686 | 98.14 |
| 7-UG-120-AL-27 | 1,273,771 | 600,111 | 146.22 | 595,057 | 99.16 | 600,111 | 186,175,427 | 46.91 | 85,713,412 | 98.14 |
| 8-UG-120-AL28 | 1,298,390 | 617,641 | 146.29 | 612,371 | 99.15 | 617,641 | 189,868,340 | 47.37 | 88,246,434 | 98.13 |
| 9-UG-120-AL29 | 1,387,817 | 662,110 | 146.16 | 656,333 | 99.13 | 662,110 | 202,728,344 | 47.5 | 94,543,810 | 98.17 |
| 10-UG-120AL-30 | 1,140,080 | 543,231 | 146.32 | 538,587 | 99.15 | 543,231 | 166,759,288 | 47.44 | 77,647,853 | 98.15 |
| 11-UG-45-DE-13 | 1,334,565 | 630,296 | 146.87 | 624,727 | 99.12 | 630,296 | 195,972,052 | 47.01 | 90,387,145 | 98.12 |
| 12-UG-45-DE-14 | 1,374,033 | 671,092 | 146.29 | 665,997 | 99.24 | 671,092 | 200,981,695 | 48.64 | 96,154,110 | 98.35 |
| 13-UG-45-DE-14 | 1,278,349 | 601,707 | 146.38 | 596,670 | 99.16 | 601,707 | 187,035,362 | 46.87 | 86,000,247 | 98.09 |
| 14-UG-45-DE-16 | 1,239,450 | 577,083 | 146.59 | 572,288 | 99.17 | 577,083 | 181,592,825 | 46.37 | 82,629,677 | 98.13 |
| 15-UG-45-DE-17 | 1,496,537 | 699,967 | 145.87 | 693,945 | 99.14 | 699,967 | 218,290,038 | 46.55 | 99,721,315 | 98.13 |
| 16-UG-45-DE-18 | 1,253,277 | 590,200 | 146.08 | 585,148 | 99.14 | 590,200 | 183,014,427 | 46.89 | 84,220,469 | 98.14 |
| 17-UG-60-DE-19 | 1,250,404 | 586,036 | 146.03 | 581,185 | 99.17 | 586,036 | 182,606,039 | 46.65 | 83,658,775 | 98.21 |
| 18-UG-60-DE-20 | 979,583 | 463,348 | 146.51 | 459,391 | 99.15 | 463,348 | 143,472,798 | 47.09 | 66,328,683 | 98.17 |
| 19-UG-60-DE-25 | 1,386,951 | 647,093 | 146.24 | 641,355 | 99.11 | 647,093 | 202,760,807 | 46.44 | 92,354,632 | 98.08 |
| 20-UG-60-DE-30 | 1,451,945 | 696,634 | 146.21 | 690,561 | 99.13 | 696,634 | 212,174,838 | 47.77 | 99,488,067 | 98.15 |

**Table S5:** Coverage metrics of the targeted sequencing relative to the reference genome

|  | **Total bases of reads (includes bases of unmatched reads)** | **Uniquely Aligned Bases (%)** | **Bases of reads within targeted regions** | **Bases of reads within targeted regions (%)** | **Genome targeted (%)** | **Enrichment in targeted regions (fold)** | **Targeted regions covered by at least 1 read** | **Targeted regions covered by at least 20 read** | **Targeted regions covered by at least 40 read** | **Duplicates** | **Duplicates (%)** |
| --- | --- | --- | --- | --- | --- | --- | --- | --- | --- | --- | --- |
| 1-CMRPER-AL10 | 212,058,508 | 45.88 | 95,525,462 | 98.18 | 2.03 | 48.9 | 672 | 503 | 489 | 83,081 | 23.04 |
| 2-CMRPER-AL-13 | 190,053,176 | 46.38 | 86,534,865 | 98.18 | 2.03 | 48.91 | 670 | 499 | 484 | 71,452 | 21.91 |
| 3-CMRPER-AL24 | 216,033,880 | 47.19 | 100,195,452 | 98.29 | 2.03 | 48.93 | 665 | 507 | 486 | 96,931 | 25.57 |
| 4-CMRPER-AL-42 | 199,903,027 | 50.2 | 98,914,305 | 98.57 | 2.03 | 49 | 634 | 503 | 484 | 108,515 | 29.36 |
| 5-CMRPER-AL-45 | 215,344,383 | 49.21 | 104,156,110 | 98.29 | 2.03 | 48.94 | 668 | 505 | 491 | 102,505 | 26.22 |
| 6-CMRPER-AL-46 | 198,485,659 | 49.14 | 95,980,212 | 98.4 | 2.03 | 48.95 | 651 | 505 | 483 | 99,911 | 27.5 |
| 7-CMRPER-AL-47 | 191,671,109 | 45.34 | 85,298,688 | 98.15 | 2.03 | 48.9 | 682 | 503 | 485 | 67,811 | 21.09 |
| 8-CMR-PER-AL-52 | 217,794,186 | 48.94 | 104,886,561 | 98.4 | 2.03 | 48.97 | 657 | 506 | 490 | 108,367 | 27.53 |
| 9-CMR-PER-AL-53 | 223,121,542 | 47.68 | 104,445,463 | 98.18 | 2.03 | 48.91 | 666 | 502 | 482 | 76,926 | 19.74 |
| 10-CMR-AL-54 | 237,818,738 | 47.34 | 110,489,380 | 98.14 | 2.03 | 48.89 | 707 | 505 | 485 | 91,223 | 21.83 |
| 11-CMR-DE-15 | 212,870,775 | 47.52 | 99,338,543 | 98.2 | 2.03 | 48.92 | 651 | 497 | 480 | 82,191 | 21.95 |
| 12-CMR-PER-DE-16 | 167,225,798 | 46.75 | 76,649,359 | 98.04 | 2.03 | 48.88 | 645 | 495 | 474 | 55,396 | 19.14 |
| 13-CMR-PER-DE-24 | 211,435,147 | 49.17 | 102,037,646 | 98.14 | 2.03 | 48.89 | 653 | 502 | 480 | 88,395 | 23.08 |
| 14-CMR-PER-DE-26 | 201,139,392 | 46.58 | 91,981,226 | 98.18 | 2.03 | 48.92 | 669 | 500 | 477 | 70,002 | 20.16 |
| 15-CMR-PER-DE-27 | 230,998,866 | 46.39 | 105,117,347 | 98.08 | 2.03 | 48.89 | 688 | 500 | 484 | 80,951 | 20.41 |
| 16-CMRPER-DE-31 | 148,380,850 | 47.39 | 69,345,403 | 98.61 | 2.03 | 48.99 | 615 | 492 | 468 | 90,462 | 33.1 |
| 17-CMRPER-DE-33 | 214,364,726 | 45.82 | 96,390,537 | 98.14 | 2.03 | 48.92 | 725 | 493 | 474 | 63,228 | 17.5 |
| 18-CMRPER-DE-34 | 215,475,774 | 46.62 | 98,692,646 | 98.24 | 2.03 | 48.93 | 652 | 498 | 480 | 84,881 | 22.61 |
| 19-CMRPER-DE-35 | 218,897,170 | 47.73 | 102,665,765 | 98.27 | 2.03 | 48.93 | 658 | 495 | 481 | 88,498 | 22.63 |
| 20-CMRPER-DE-40 | 224,025,330 | 46.57 | 102,333,214 | 98.09 | 2.03 | 48.89 | 655 | 494 | 477 | 80,820 | 20.82 |
| 10-Fang-05 | 161,413,660 | 47.34 | 75,067,812 | 98.24 | 2.03 | 48.91 | 679 | 505 | 481 | 67,030 | 23.86 |
| 11-Fang-08 | 143,508,602 | 47.79 | 67,465,084 | 98.38 | 2.03 | 48.94 | 652 | 498 | 472 | 64,577 | 25.61 |
| 12-Fang-11 | 161,345,675 | 47.05 | 74,589,201 | 98.26 | 2.03 | 48.92 | 636 | 501 | 479 | 67,965 | 24.39 |
| 13-Fang-19 | 156,487,603 | 48.03 | 73,866,012 | 98.27 | 2.03 | 48.92 | 644 | 503 | 479 | 70,476 | 25.61 |
| 14-Fang-22 | 165,386,917 | 47.16 | 76,652,638 | 98.28 | 2.03 | 48.92 | 658 | 501 | 479 | 72,055 | 25.22 |
| 15-Fang-24 | 140,184,338 | 47.13 | 64,895,384 | 98.23 | 2.03 | 48.91 | 674 | 498 | 471 | 55,227 | 22.76 |
| 16-Fang-26 | 163,614,721 | 46.88 | 75,387,315 | 98.28 | 2.03 | 48.92 | 662 | 503 | 480 | 65,910 | 23.48 |
| 18-Fang-31 | 145,756,053 | 47.98 | 68,716,208 | 98.25 | 2.03 | 48.92 | 643 | 490 | 472 | 56,939 | 22.2 |
| 9-Fang-03 | 149,018,273 | 47.24 | 69,164,990 | 98.26 | 2.03 | 48.92 | 660 | 498 | 476 | 58,407 | 22.69 |
| 17-Fang28 | 138,015,671 | 47.87 | 64,896,689 | 98.22 | 2.03 | 48.91 | 628 | 487 | 466 | 51,327 | 21.2 |
| 11-MALPER-180-AL | 71,765,820 | 48.73 | 34,364,274 | 98.26 | 2.03 | 48.91 | 581 | 471 | 447 | 27,842 | 21.59 |
| 12-MALPER-180-AL | 71,844,378 | 48.42 | 34,163,804 | 98.2 | 2.03 | 48.88 | 570 | 471 | 450 | 27,665 | 21.57 |
| 13-MALPER-180-AL | 71,925,924 | 47.77 | 33,724,155 | 98.14 | 2.03 | 48.87 | 591 | 471 | 445 | 25,728 | 20.27 |
| 14-MALPER-180-AL | 72,220,462 | 47.71 | 33,821,997 | 98.16 | 2.03 | 48.89 | 588 | 469 | 448 | 22,262 | 17.66 |
| 15-MALPER-180-AL | 71,912,716 | 47.94 | 33,863,627 | 98.23 | 2.03 | 48.89 | 584 | 468 | 449 | 25,251 | 19.94 |
| 16-MALPER-180-AL | 71,972,652 | 48.13 | 34,017,217 | 98.2 | 2.03 | 48.9 | 583 | 472 | 451 | 22,740 | 17.95 |
| 17-MALPER-180-AL | 72,122,692 | 48.88 | 34,632,504 | 98.23 | 2.03 | 48.9 | 589 | 470 | 451 | 24,532 | 19 |
| 18-MALPER-180-AL | 72,065,596 | 48.48 | 34,328,510 | 98.26 | 2.03 | 48.92 | 578 | 470 | 448 | 23,738 | 18.51 |
| 19-MALPER-A180-AL | 71,804,783 | 48.63 | 34,313,105 | 98.26 | 2.03 | 48.9 | 575 | 467 | 450 | 25,905 | 20.23 |
| 20-MALPER-180-AL | 71,929,814 | 48.37 | 34,168,785 | 98.22 | 2.03 | 48.89 | 581 | 468 | 444 | 26,006 | 20.32 |
| 1-MALPER-60-DE | 71,789,195 | 47.08 | 33,236,674 | 98.33 | 2.03 | 48.91 | 571 | 471 | 456 | 27,134 | 21.83 |
| 2-MALPER-60-DE | 71,653,236 | 47.52 | 33,457,294 | 98.25 | 2.03 | 48.9 | 604 | 474 | 451 | 27,851 | 22.35 |
| 3-MALPER-60-DE | 72,039,423 | 46.67 | 33,054,397 | 98.32 | 2.03 | 48.93 | 581 | 472 | 447 | 28,076 | 22.91 |
| 4-MALPER-60-DE | 71,789,676 | 47.7 | 33,667,418 | 98.32 | 2.03 | 48.92 | 581 | 474 | 452 | 31,295 | 24.97 |
| 5-MALPER-60-DE | 71,867,697 | 48.21 | 34,049,999 | 98.29 | 2.03 | 48.9 | 588 | 478 | 450 | 29,175 | 23 |
| 6-MALPER-60-DE | 72,226,542 | 47.2 | 33,540,124 | 98.39 | 2.03 | 48.95 | 579 | 468 | 451 | 30,772 | 24.34 |
| 7-MALPER-60-DE | 72,304,472 | 46.12 | 32,744,243 | 98.19 | 2.03 | 48.9 | 590 | 471 | 446 | 24,137 | 19.66 |
| 8-MALPER-60-DE | 71,398,747 | 47.85 | 33,575,249 | 98.27 | 2.03 | 48.92 | 592 | 472 | 451 | 26,534 | 21.02 |
| 9-MALPER-60-DE | 72,172,467 | 47.26 | 33,499,906 | 98.22 | 2.03 | 48.9 | 577 | 471 | 449 | 23,108 | 18.51 |
| 10-MALPER-60-DE | 72,004,816 | 48.15 | 34,066,436 | 98.25 | 2.03 | 48.9 | 584 | 473 | 442 | 26,987 | 21.17 |
| 1-UG-120-AL-14 | 182,980,590 | 48.5 | 87,091,420 | 98.14 | 2.03 | 48.9 | 640 | 490 | 471 | 68,423 | 21.12 |
| 2-UG-120-AL-18 | 192,223,402 | 47.71 | 90,034,423 | 98.18 | 2.03 | 48.92 | 665 | 488 | 478 | 70,399 | 20.96 |
| 3-UG-120-AL-19 | 188,877,466 | 47.15 | 87,394,082 | 98.13 | 2.03 | 48.9 | 684 | 494 | 473 | 64,385 | 19.73 |
| 4-UG-120-AL-20 | 182,886,725 | 47.88 | 85,989,252 | 98.21 | 2.03 | 48.92 | 661 | 497 | 474 | 67,882 | 21.1 |
| 5-UG-120-AL-25 | 184,826,709 | 46.81 | 84,860,909 | 98.09 | 2.03 | 48.9 | 676 | 487 | 473 | 54,927 | 17.42 |
| 6-UG-120-AL-26 | 189,606,544 | 47.05 | 87,558,686 | 98.14 | 2.03 | 48.89 | 667 | 490 | 469 | 62,183 | 19.06 |
| 7-UG-120-AL-27 | 186,175,427 | 46.91 | 85,713,412 | 98.14 | 2.03 | 48.91 | 658 | 488 | 470 | 57,243 | 17.96 |
| 8-UG-120-AL28 | 189,868,340 | 47.37 | 88,246,434 | 98.13 | 2.03 | 48.91 | 659 | 490 | 471 | 58,481 | 17.87 |
| 9-UG-120-AL29 | 202,728,344 | 47.5 | 94,543,810 | 98.17 | 2.03 | 48.9 | 652 | 491 | 471 | 64,805 | 18.5 |
| 10-UG-120AL-30 | 166,759,288 | 47.44 | 77,647,853 | 98.15 | 2.03 | 48.91 | 673 | 487 | 470 | 53,083 | 18.35 |
| 11-UG-45-DE-13 | 195,972,052 | 47.01 | 90,387,145 | 98.12 | 2.03 | 48.89 | 688 | 493 | 477 | 65,936 | 19.53 |
| 12-UG-45-DE-14 | 200,981,695 | 48.64 | 96,154,110 | 98.35 | 2.03 | 48.95 | 638 | 493 | 473 | 85,496 | 23.86 |
| 13-UG-45-DE-14 | 187,035,362 | 46.87 | 86,000,247 | 98.09 | 2.03 | 48.92 | 675 | 487 | 471 | 51,802 | 16.24 |
| 14-UG-45-DE-16 | 181,592,825 | 46.37 | 82,629,677 | 98.13 | 2.03 | 48.92 | 655 | 486 | 471 | 50,824 | 16.57 |
| 15-UG-45-DE-17 | 218,290,038 | 46.55 | 99,721,315 | 98.13 | 2.03 | 48.9 | 682 | 492 | 473 | 69,273 | 18.66 |
| 16-UG-45-DE-18 | 183,014,427 | 46.89 | 84,220,469 | 98.14 | 2.03 | 48.91 | 638 | 486 | 468 | 52,668 | 16.94 |
| 17-UG-60-DE-19 | 182,606,039 | 46.65 | 83,658,775 | 98.21 | 2.03 | 48.92 | 633 | 483 | 469 | 64,623 | 20.71 |
| 18-UG-60-DE-20 | 143,472,798 | 47.09 | 66,328,683 | 98.17 | 2.03 | 48.91 | 643 | 483 | 466 | 45,144 | 18.28 |
| 19-UG-60-DE-25 | 202,760,807 | 46.44 | 92,354,632 | 98.08 | 2.03 | 48.89 | 658 | 490 | 473 | 63,239 | 18.42 |
| 20-UG-60-DE-30 | 212,174,838 | 47.77 | 99,488,067 | 98.15 | 2.03 | 48.9 | 640 | 490 | 475 | 71,477 | 19.41 |

**Table S6:** Summary of significant SNPs detected between the permethrin resistant (R) and field dead mosquitoes (C) (R-C) in Cameroon, Malawi and Uganda.

| **Transcripts** | **Nsyn** | | **SpS** | | **Syn** | | | **Int** | | **5'UTR** | | **3'UTR** | | **Description** | |  |  |
| --- | --- | --- | --- | --- | --- | --- | --- | --- | --- | --- | --- | --- | --- | --- | --- | --- | --- |
|  |  | |  | |  | | |  | |  | |  | | **Cameroon** | |  |  |
| AFUN000288 | 4 | | 0 | | 2 | | | 0 | | 0 | | 0 | | APL1C | |  |  |
| AFUN015793 | 3 | | 0 | | 5 | | | 0 | | 0 | | 0 | | Carboxylesterase | |  |  |
| AFUN001383 | 2 | | 0 | | 1 | | | 0 | | 0 | | 0 | | CYP9J11__cytochrome_P450 | |  |  |
| AFUN009936 | 2 | | 0 | | 2 | | | 0 | | 0 | | 0 | | Cuticle protein | |  |  |
| AFUN011531 | 2 | | 0 | | 0 | | | 0 | | 0 | | 0 | | Carboxylesterase | |  |  |
| AFUN000518 | 1 | | 1 | | 1 | | | 0 | | 0 | | 0 | | CYP6AK1__cytochrome_P450 | |  |  |
| AFUN001223 | 1 | | 0 | | 0 | | | 0 | | 0 | | 0 | | ATP-binding cassette sub-family A member 1-like | |  |  |
| AFUN004976 | 1 | | 1 | | 3 | | | 0 | | 0 | | 0 | | UDP-glucuronosyltransferase 2A3 | |  |  |
| AFUN006361 | 1 | | 0 | | 2 | | | 0 | | 0 | | 0 | | Leucine-rich repeat transmembrane neuronal 2 | |  |  |
| AFUN007491 | 1 | | 0 | | 0 | | | 0 | | 0 | | 0 | | Peritrophin-1 | |  |  |
| AFUN008426 | 1 | | 0 | | 4 | | | 0 | | 0 | | 0 | | GSTU2__glutathione_S-transferase | |  |  |
| AFUN009068 | 1 | | 0 | | 0 | | | 0 | | 0 | | 0 | | Carboxylesterase | |  |  |
| AFUN010230 | 1 | | 0 | | 0 | | | 0 | | 0 | | 0 | | Transmembrane protease serine 9 | |  |  |
| AFUN010919 | 1 | | 0 | | 1 | | | 0 | | 0 | | 0 | | CYP6M1c__cytochrome_P450 | |  |  |
| AFUN000371 | 0 | | 0 | | 0 | | | 1 | | 0 | | 0 | | Carboxylesterase | |  |  |
| AFUN000373 | 0 | | 0 | | 2 | | | 0 | | 0 | | 0 | | Carboxylesterase | |  |  |
| AFUN001382 | 0 | | 0 | | 1 | | | 0 | | 0 | | 0 | | CYP9J11__cytochrome_P450 | |  |  |
| AFUN002021 | 0 | | 0 | | 1 | | | 0 | | 0 | | 0 | | alpha-amylase | |  |  |
| AFUN002116 | 0 | | 0 | | 1 | | | 0 | | 0 | | 0 | | toll-like receptor 13 | |  |  |
| AFUN002428 | 0 | | 0 | | 4 | | | 0 | | 0 | | 0 | | aminopeptidase | |  |  |
| AFUN005498 | 0 | | 0 | | 3 | | | 0 | | 0 | | 0 | | Ecdysteroid UDP-glucosyltransferase Flags | |  |  |
| AFUN005694 | 0 | | 0 | | 0 | | | 1 | | 0 | | 0 | | Acetylcholinesterase | |  |  |
| AFUN006951 | 0 | | 0 | | 1 | | | 0 | | 0 | | 0 | | Transmembrane protease serine 9 | |  |  |
| AFUN007162 | 0 | | 0 | | 0 | | | 1 | | 0 | | 0 | | ABC__ATP-binding_cassette | |  |  |
| AFUN007217 | 0 | | 1 | | 2 | | | 0 | | 0 | | 0 | | Heat shock 75 mitochondrial | |  |  |
| AFUN007480 | 0 | | 0 | | 0 | | | 0 | | 0 | | 2 | | Serine protease SP24D | |  |  |
| AFUN007490 | 0 | | 0 | | 0 | | | 1 | | 0 | | 0 | | NA | |  |  |
| AFUN008096 | 0 | | 0 | | 0 | | | 1 | | 0 | | 0 | | toll-like receptor 13 | |  |  |
| AFUN008357 | 0 | | 1 | | 1 | | | 0 | | 0 | | 0 | | Pseudo Cytochrome P450 | |  |  |
| AFUN008495 | 0 | | 0 | | 2 | | | 0 | | 0 | | 0 | | chymotrypsin-like elastase family member 2A | |  |  |
| AFUN008618 | 0 | | 0 | | 0 | | | 2 | | 0 | | 0 | | Peroxidase | |  |  |
| AFUN009558 | 0 | | 0 | | 1 | | | 0 | | 0 | | 0 | | Toll-like receptor | |  |  |
| AFUN009934 | 0 | | 0 | | 1 | | | 3 | | 0 | | 0 | | Cuticle protein | |  |  |
| AFUN009937 | 0 | | 0 | | 2 | | | 0 | | 0 | | 0 | | Cuticle protein | |  |  |
| AFUN010921 | 0 | | 0 | | 3 | | | 0 | | 0 | | 0 | | CYP6M1b__cytochrome_P450 | |  |  |
| AFUN011481 | 0 | | 0 | | 1 | | | 0 | | 0 | | 0 | | CYP4G17__cytochrome_P450 | |  |  |
| AFUN015723 | 0 | | 0 | | 0 | | | 1 | | 0 | | 0 | | CYP6AH1__cytochrome_P450 | |  |  |
| AFUN015785 | 0 | | 0 | | 1 | | | 0 | | 0 | | 0 | | CYP6AA2__cytochrome_P450 | |  |  |
| AFUN015787 | 0 | | 0 | | 3 | | | 0 | | 2 | | 0 | | Carboxylesterase | |  |  |
| AFUN015792 | 0 | | 0 | | 1 | | | 0 | | 0 | | 4 | | CYP6P9a__cytochrome_P450 | |  |  |
| AFUN015796 | 0 | | 0 | | 1 | | | 0 | | 0 | | 0 | | CYP6S2__cytochrome_P450 | |  |  |
| AFUN015866 | 0 | | 0 | | 1 | | | 0 | | 0 | | 0 | | CYP9J3__cytochrome_P450 | |  |  |
| AFUN015889 | 0 | | 0 | | 0 | | | 1 | | 0 | | 0 | | CYP6P9b__cytochrome_P450 | |  |  |
| AFUN015890 | 0 | | 0 | | 0 | | | 0 | | 1 | | 0 | | CYP6P4a__cytochrome_P450 | |  |  |
| **Malawi** | | | | | | | | | | | | | | | |  |  |
| AFUN000518 | | 3 | | 1 | | 1 | 1 | | 0 | | 0 | | CYP6AK1__cytochrome_P450 | |  |  |  |
| AFUN009936 | | 2 | | 0 | | 2 | 0 | | 0 | | 0 | | Cuticle protein | |  |  |  |
| AFUN010919 | | 2 | | 0 | | 4 | 0 | | 0 | | 0 | | CYP6M1c__cytochrome_P450 | |  |  |  |
| AFUN003595 | | 1 | | 0 | | 0 | 0 | | 0 | | 0 | | Xanthine dehydrogenase | |  |  |  |
| AFUN006951 | | 1 | | 0 | | 1 | 0 | | 0 | | 0 | | Transmembrane protease serine 9 | |  |  |  |
| AFUN009690 | | 1 | | 0 | | 0 | 0 | | 0 | | 0 | | chymotrypsin-like elastase | |  |  |  |
| AFUN010921 | | 1 | | 0 | | 10 | 0 | | 0 | | 0 | | CYP6M1b__cytochrome_P450 | |  |  |  |
| AFUN015774 | | 1 | | 0 | | 3 | 0 | | 0 | | 0 | | CYP12F4__cytochrome_P450 | |  |  |  |
| AFUN015796 | | 1 | | 0 | | 3 | 0 | | 0 | | 0 | | CYP6S2__cytochrome_P450 | |  |  |  |
| AFUN015891 | | 1 | | 0 | | 2 | 0 | | 0 | | 0 | | CYP6N1__cytochrome_P450 | |  |  |  |
| AFUN001382 | | 0 | | 0 | | 1 | 0 | | 0 | | 0 | | CYP9J5__cytochrome_P450 | |  |  |  |
| AFUN001383 | | 0 | | 0 | | 3 | 0 | | 0 | | 0 | | CYP9J5__cytochrome_P450 | |  |  |  |
| AFUN001746 | | 0 | | 0 | | 1 | 0 | | 0 | | 0 | | CYP4H19__cytochrome_P450 | |  |  |  |
| AFUN002428 | | 0 | | 0 | | 4 | 0 | | 0 | | 0 | | Microsomal aminopeptidase | |  |  |  |
| AFUN002569 | | 0 | | 0 | | 1 | 0 | | 0 | | 0 | | AO__aldehyde_oxidase | |  |  |  |
| AFUN004324 | | 0 | | 0 | | 1 | 0 | | 0 | | 0 | | 150 kDa TGF-beta-1-binding | |  |  |  |
| AFUN005118 | | 0 | | 0 | | 1 | 0 | | 0 | | 0 | | Opsin-1 | |  |  |  |
| AFUN005694 | | 0 | | 0 | | 0 | 1 | | 0 | | 0 | | Acetylcholinesterase 2 | |  |  |  |
| AFUN005942 | | 0 | | 0 | | 0 | 0 | | 1 | | 0 | | Mitochondrial inner membrane protease | |  |  |  |
| AFUN006361 | | 0 | | 0 | | 2 | 0 | | 0 | | 0 | | Leucine-rich repeat transmembrane neuronal | |  |  |  |
| AFUN007410 | | 0 | | 0 | | 3 | 0 | | 0 | | 0 | | Trypsin-1 | |  |  |  |
| AFUN008357 | | 0 | | 1 | | 0 | 1 | | 0 | | 0 | | Pseudo cytochrome P450 | |  |  |  |
| AFUN008495 | | 0 | | 0 | | 1 | 0 | | 0 | | 0 | | chymotrypsin-like elastase | |  |  |  |
| AFUN008618 | | 0 | | 0 | | 0 | 10 | | 0 | | 0 | | Chorion peroxidase | |  |  |  |
| AFUN009934 | | 0 | | 0 | | 0 | 3 | | 0 | | 0 | | Cuticle protein | |  |  |  |
| AFUN009937 | | 0 | | 0 | | 1 | 0 | | 0 | | 0 | | Cuticle protein | |  |  |  |
| AFUN015962 | | 0 | | 0 | | 1 | 0 | | 0 | | 0 | | CYP6N2__cytochrome_P450 | |  |  |  |
| AFUN015980 | | 0 | | 1 | | 0 | 0 | | 0 | | 0 | | ABC__ATP-binding_cassette | |  |  |  |
| **Uganda** | | | | | | | | | | | | | | | |  |  |
| AFUN000597 | 3 | | 0 | | 0 | | | 1 | | 0 | | 0 | | Leucine-rich repeat-containing 15, APL1C | |  |  |
| AFUN001746 | 2 | | 0 | | 2 | | | 0 | | 0 | | 0 | | CYP4H19_Cytochrome P450 | |  |  |
| AFUN009934 | 2 | | 0 | | 4 | | | 1 | | 0 | | 0 | | Cuticle protein | |  |  |
| AFUN000518 | 1 | | 1 | | 0 | | | 0 | | 0 | | 0 | | CYP6AK1__cytochrome_P450 | |  |  |
| AFUN004976 | 1 | | 0 | | 1 | | | 0 | | 0 | | 0 | | UDP-glucuronosyltransferase 2A3 | |  |  |
| AFUN005498 | 1 | | 0 | | 3 | | | 0 | | 0 | | 0 | | Ecdysteroid UDP-glucosyltransferase Flags | |  |  |
| AFUN008495 | 1 | | 0 | | 0 | | | 0 | | 0 | | 0 | | chymotrypsin-like elastase family member 2A | |  |  |
| AFUN000288 | 0 | | 0 | | 1 | | | 0 | | 0 | | 0 | | Insulin-like growth factor-binding complex acid labile, APL1C | |  |  |
| AFUN000866 | 0 | | 0 | | 0 | | | 2 | | 0 | | 0 | | serpin 4 inhibitory serine protease partial | |  |  |
| AFUN002021 | 0 | | 0 | | 1 | | | 0 | | 0 | | 0 | | alpha-amylase | |  |  |
| AFUN002428 | 0 | | 0 | | 2 | | | 0 | | 0 | | 0 | | aminopeptidase | |  |  |
| AFUN004246 | 0 | | 0 | | 0 | | | 0 | | 0 | | 2 | | Acetyl-coenzyme A synthetase | |  |  |
| AFUN005259 | 0 | | 0 | | 0 | | | 2 | | 0 | | 0 | | ABC__ATP-binding_cassette | |  |  |
| AFUN006361 | 0 | | 0 | | 3 | | | 0 | | 0 | | 0 | | Leucine-rich repeat transmembrane neuronal 2 | |  |  |
| AFUN008357 | 0 | | 0 | | 1 | | | 0 | | 0 | | 0 | | Pseudo Cytochrome P450 | |  |  |
| AFUN008618 | 0 | | 0 | | 0 | | | 5 | | 0 | | 0 | | Chorion peroxidase | |  |  |
| AFUN008975 | 0 | | 0 | | 4 | | | 0 | | 0 | | 0 | | ABC__ATP-binding_cassette | |  |  |
| AFUN009786 | 0 | | 0 | | 0 | | | 2 | | 0 | | 0 | | solute carrier family facilitated glucose transporter | |  |  |
| AFUN009935 | 0 | | 0 | | 0 | | | 2 | | 0 | | 0 | | Cuticle protein | |  |  |
| AFUN009936 | 0 | | 0 | | 1 | | | 0 | | 0 | | 0 | | Cuticle protein | |  |  |
| AFUN009937 | 0 | | 0 | | 1 | | | 0 | | 0 | | 0 | | Cuticle protein | |  |  |
| AFUN010230 | 0 | | 0 | | 2 | | | 0 | | 0 | | 0 | | Transmembrane protease serine 9 | |  |  |
| AFUN010918 | 0 | | 0 | | 5 | | | 0 | | 0 | | 0 | | CYP6N1__cytochrome_P450 | |  |  |
| AFUN010921 | 0 | | 0 | | 3 | | | 0 | | 0 | | 0 | | CYP6M1c__cytochrome_P450 | |  |  |
| AFUN014090 | 0 | | 0 | | 0 | | | 0 | | 3 | | 0 | | Leucine-rich repeat-containing 15 | |  |  |
| AFUN015745 | 0 | | 0 | | 0 | | | 0 | | 0 | | 1 | | ABC__ATP-binding_cassette | |  |  |
| AFUN015792 | 0 | | 0 | | 1 | | | 0 | | 0 | | 0 | | CYP6P9a__cytochrome_P450 | |  |  |
| AFUN015795 | 0 | | 0 | | 1 | | | 0 | | 0 | | 0 | | CYP6M7__cytochrome_P450 | |  |  |
| AFUN015796 | 0 | | 0 | | 3 | | | 0 | | 0 | | 0 | | CYP6S2__cytochrome_P450 | |  |  |

NSyn, Non synonymous substitution; SpS, Splice Site; Syn, Synonymous site; Int: Intron

**Table S7: Significant SNPs between Malawi and FANG.**

| **Identifier** | **Nsyn** | **SpS** | **Syn** | **Int** | **5'UTR** | **3'UTR** | **Description** |
| --- | --- | --- | --- | --- | --- | --- | --- |
| AFUN005377 | 8 | 0 | 19 | 0 | 0 | 0 | ABC__ATP-binding_cassette |
| AFUN003368 | 6 | 1 | 7 | 47 | 0 | 0 | Glycogenin-1 |
| AFUN014380 | 6 | 0 | 2 | 0 | 0 | 4 | AO__aldehyde_oxidase |
| AFUN015775 | 4 | 1 | 3 | 0 | 4 | 3 | CYP12F2__cytochrome_P450_(CYP12F2) |
| AFUN015801 | 4 | 0 | 6 | 0 | 0 | 0 | CYP6P2__cytochrome_P450_(CYP6P2) |
| AFUN005041 | 4 | 0 | 4 | 0 | 0 | 0 | ATP-binding cassette sub-family G member 2-like |
| AFUN015759 | 4 | 0 | 2 | 0 | 0 | 0 | UDP-glucuronosyltransferase 2B8 Precursor |
| AFUN015723 | 3 | 0 | 5 | 0 | 5 | 2 | CYP6AH1__cytochrome_P450_(CYP6AH1) |
| AFUN002573 | 3 | 0 | 6 | 3 | 2 | 0 | Esterase B6 |
| AFUN015866 | 3 | 0 | 1 | 0 | 2 | 0 | CYP9J3__cytochrome_P450_(CYP9J3) |
| AFUN015962 | 3 | 0 | 3 | 0 | 1 | 1 | CYP6N2__cytochrome_P450_(CYP6N2) |
| AFUN015862 | 3 | 2 | 9 | 0 | 0 | 0 | CYP9L1__cytochrome_P450_(CYP9L1) |
| AFUN015774 | 3 | 2 | 8 | 0 | 0 | 0 | CYP12F4__cytochrome_P450_(CYP12F4) |
| AFUN007297 | 3 | 0 | 20 | 2 | 0 | 3 | ALDH__aldehyde_dehydrogenase |
| AFUN004352 | 3 | 0 | 7 | 1 | 0 | 0 | UDP-glucuronosyltransferase 2A3 |
| AFUN000422 | 3 | 0 | 2 | 7 | 0 | 0 | Carboxylesterase |
| AFUN009234 | 3 | 0 | 1 | 0 | 0 | 0 | GSTU1__glutathione_S-transferase_(GSTU1) |
| AFUN015865 | 2 | 0 | 11 | 0 | 4 | 4 | CYP9J4__cytochrome_P450_(CYP9J4) |
| AFUN015786 | 2 | 0 | 17 | 0 | 1 | 1 | CYP6AA1__cytochrome_P450_(CYP6AA1) |
| AFUN001382 | 2 | 0 | 12 | 2 | 0 | 1 | CYP9J5__cytochrome_P450_(CYP9J5) |
| AFUN005102 | 2 | 0 | 7 | 0 | 0 | 0 | CYP325G1__cytochrome_P450_(CYP325G1) |
| AFUN005498 | 2 | 0 | 6 | 5 | 0 | 0 | Ecdysteroid UDP-glucosyltransferase |
| AFUN009304 | 2 | 0 | 6 | 0 | 0 | 0 | ATP-binding cassette sub-family A member 1-like |
| AFUN016010 | 2 | 0 | 5 | 3 | 0 | 1 | GSTD1__glutathione_S-transferase_(GSTD1) |
| AFUN015760 | 2 | 0 | 5 | 0 | 0 | 1 | UDP-glucuronosyltransferase 2B15 |
| AFUN001223 | 2 | 0 | 4 | 0 | 0 | 2 | ATP-binding cassette sub-family A member 1-like |
| AFUN005864 | 2 | 0 | 3 | 0 | 0 | 3 | CYP4AR1__cytochrome_P450_(CYP4AR1) |
| AFUN015791 | 2 | 0 | 2 | 5 | 0 | 1 | CYP6AG2__cytochrome_P450_(CYP6AG2) |
| AFUN010202 | 2 | 0 | 2 | 0 | 0 | 0 | Glutactin esterase |
| AFUN005786 | 2 | 0 | 1 | 0 | 0 | 0 | UDP-glucuronosyltransferase 1-1 |
| AFUN007717 | 2 | 0 | 0 | 0 | 0 | 0 | CYP325H1__cytochrome_P450_(CYP325H1) |
| AFUN015906 | 2 | 0 | 0 | 0 | 0 | 0 | CYP4J9__cytochrome_P450_(CYP4J9) |
| AFUN007590 | 1 | 1 | 4 | 1 | 14 | 5 | ABC__ATP-binding_cassette |
| AFUN015790 | 1 | 0 | 4 | 0 | 13 | 7 | CYP6AG1__cytochrome_P450_(CYP6AG1) |
| AFUN007162 | 1 | 0 | 1 | 36 | 7 | 0 | ABC__ATP-binding_cassette |
| AFUN011481 | 1 | 0 | 5 | 0 | 6 | 0 | CYP4G17__cytochrome_P450_(CYP4G17) |
| AFUN005260 | 1 | 0 | 15 | 86 | 4 | 11 | ABC__ATP-binding_cassette |
| AFUN002180 | 1 | 0 | 16 | 0 | 0 | 0 | ABC__ATP-binding_cassette |
| AFUN015889 | 1 | 0 | 15 | 0 | 0 | 3 | CYP6P9b__cytochrome_P450_(CYP6P9b) |
| AFUN001449 | 1 | 0 | 14 | 3 | 0 | 0 | ABC__ATP-binding_cassette |
| AFUN010472 | 1 | 0 | 8 | 2 | 0 | 0 | GPI mannosyltransferase 4 |
| AFUN001383 | 1 | 0 | 8 | 0 | 0 | 0 | CYP9J5__cytochrome_P450_(CYP9J5) |
| AFUN010919 | 1 | 0 | 7 | 0 | 0 | 0 | C6A13_DROME ame: Full=Probable cytochrome P450 6a13 |
| AFUN015891 | 1 | 0 | 7 | 0 | 0 | 0 | cytochrome P450 6a18 |
| AFUN005101 | 1 | 0 | 5 | 0 | 0 | 2 | CYP325F2__cytochrome_P450_(CYP325F2) |
| AFUN015978 | 1 | 0 | 5 | 0 | 0 | 0 | ABC__ATP-binding_cassette |
| AFUN007483 | 1 | 0 | 5 | 0 | 0 | 0 | ABC__ATP-binding_cassette |
| AFUN010918 | 1 | 0 | 5 | 0 | 0 | 0 | CYP6N1__cytochrome_P450_(CYP6N1) |
| AFUN009063 | 1 | 0 | 4 | 0 | 0 | 5 | UDP-glucuronosyltransferase 2B14 |
| AFUN015864 | 1 | 0 | 4 | 0 | 0 | 0 | CYP9L2__cytochrome_P450_(CYP9L2) |
| AFUN010552 | 1 | 0 | 3 | 0 | 0 | 0 | ABC__ATP-binding_cassette |
| AFUN004872 | 1 | 0 | 3 | 0 | 0 | 0 | Neurotactin |
| AFUN009068 | 1 | 0 | 2 | 1 | 0 | 0 | Carboxylesterase |
| AFUN002390 | 1 | 0 | 2 | 0 | 0 | 0 | Chorion peroxidase |
| AFUN008618 | 1 | 0 | 2 | 0 | 0 | 0 | Chorion peroxidase |
| AFUN002517 | 1 | 0 | 1 | 0 | 0 | 6 | carboxylesterase |
| AFUN015785 | 1 | 0 | 1 | 0 | 0 | 3 | CYP6AA2__cytochrome_P450_(CYP6AA2) |
| AFUN002514 | 1 | 0 | 1 | 0 | 0 | 0 | Carboxylesterase |
| AFUN015793 | 1 | 0 | 1 | 0 | 0 | 0 | Carboxylesterase |
| AFUN002449 | 1 | 0 | 1 | 0 | 0 | 0 | Chorion peroxidase |
| AFUN015792 | 1 | 0 | 1 | 0 | 0 | 0 | CYP6P9a__cytochrome_P450_(CYP6P9a) |
| AFUN015811 | 1 | 0 | 1 | 0 | 0 | 0 | GSTE5__glutathione_S-transferase_(GSTE5) |
| AFUN003693 | 1 | 0 | 0 | 0 | 0 | 0 | ABC__ATP-binding_cassette |
| AFUN015979 | 1 | 0 | 0 | 0 | 0 | 0 | ABC__ATP-binding_cassette |
| AFUN004798 | 1 | 0 | 0 | 0 | 0 | 0 | ATP-binding cassette sub-family G member 2-like |
| AFUN015904 | 1 | 0 | 0 | 0 | 0 | 0 | CYP4J5__cytochrome_P450_(CYP4J5) |
| AFUN016008 | 1 | 0 | 0 | 0 | 0 | 0 | GSTE6__glutathione_S-transferase_(GSTE6) |
| AFUN011189 | 1 | 0 | 0 | 0 | 0 | 0 | UDP-glucuronosyltransferase 2B33 |
| AFUN010525 | 0 | 0 | 5 | 5 | 16 | 8 | ABC__ATP-binding_cassette |
| AFUN015745 | 0 | 0 | 6 | 11 | 10 | 0 | ABC__ATP-binding_cassette |
| AFUN003452 | 0 | 0 | 7 | 0 | 8 | 0 | ALDH__aldehyde_dehydrogenase_(NAD+) |
| AFUN002796 | 0 | 0 | 14 | 2 | 7 | 8 | ABC__ATP-binding_cassette |
| AFUN002408 | 0 | 0 | 0 | 0 | 4 | 0 | CYP329A1__cytochrome_P450_(CYP329A1) |
| AFUN015980 | 0 | 0 | 4 | 0 | 3 | 0 | ABC__ATP-binding_cassette |
| AFUN015726 | 0 | 0 | 0 | 0 | 3 | 0 | BIB_DROME ame: Full=Neurogenic big brain |
| AFUN006122 | 0 | 0 | 0 | 0 | 3 | 0 | CYP4C27__cytochrome_P450_(CYP4C27) |
| AFUN010428 | 0 | 0 | 0 | 4 | 2 | 0 | CYP4G16__cytochrome_P450_(CYP4G16) |
| AFUN010004 | 0 | 1 | 1 | 8 | 1 | 0 | ABC__ATP-binding_cassette |
| AFUN015863 | 0 | 0 | 5 | 0 | 1 | 0 | CP9F2_DROME ame: Full=Probable cytochrome P450 9f2 |
| AFUN015795 | 0 | 0 | 2 | 0 | 1 | 3 | CYP6M7__cytochrome_P450_(CYP6M7) |
| AFUN009061 | 0 | 0 | 1 | 0 | 1 | 1 | GSTO1__glutathione_S-transferase_(GSTO1) |
| AFUN015743 | 0 | 0 | 0 | 8 | 1 | 0 | C49A1_DROME ame: Full=Probable cytochrome P450 49a1 |
| AFUN002128 | 0 | 0 | 0 | 0 | 1 | 0 | CYP4C25__cytochrome_P450_(CYP4C25) |
| AFUN005259 | 0 | 1 | 7 | 8 | 0 | 11 | ABC__ATP-binding_cassette |
| AFUN008521 | 0 | 0 | 9 | 0 | 0 | 4 | ABC__ATP-binding_cassette |
| AFUN008975 | 0 | 0 | 7 | 3 | 0 | 4 | ABC__ATP-binding_cassette |
| AFUN015919 | 0 | 0 | 6 | 0 | 0 | 4 | CYP6Z1__cytochrome_P450_(CYP6Z1) |
| AFUN015982 | 0 | 0 | 5 | 0 | 0 | 3 | ABC__ATP-binding_cassette |
| AFUN007549 | 0 | 0 | 5 | 0 | 0 | 2 | CYP9K1__cytochrome_P450_(CYP9K1) |
| AFUN005694 | 0 | 0 | 4 | 0 | 0 | 1 | ACES_ANOST ame: Full=Acetylcholinesterase |
| AFUN006135 | 0 | 0 | 1 | 0 | 0 | 1 | CYP4C36__cytochrome_P450_(CYP4C36) |
| AFUN015993 | 0 | 0 | 1 | 0 | 0 | 1 | CYP4D15__cytochrome_P450_(CYP4D15) |
| AFUN000995 | 0 | 0 | 0 | 17 | 0 | 1 | Aquaporin |
| AFUN015923 | 0 | 0 | 0 | 0 | 0 | 8 | Aquaporin |
| AFUN001673 | 0 | 0 | 0 | 0 | 0 | 3 | ABC__ATP-binding_cassette |
| AFUN001682 | 0 | 0 | 0 | 0 | 0 | 2 | ABC__ATP-binding_cassette |
| AFUN015758 | 0 | 0 | 0 | 0 | 0 | 2 | UDP-glucuronosyltransferase 2B31 |
| AFUN015908 | 0 | 0 | 0 | 0 | 0 | 1 | CYP15B1__cytochrome_P450_(CYP15B1) |
| AFUN015957 | 0 | 0 | 0 | 0 | 0 | 1 | CYP304C1__cytochrome_P450_(CYP304C1) |
| AFUN015739 | 0 | 0 | 0 | 0 | 0 | 1 | CYP307A1__cytochrome_P450_(CYP307A1) |
| AFUN005865 | 0 | 0 | 0 | 0 | 0 | 1 | CYP4K2__cytochrome_P450_(CYP4K2) |
| AFUN015839 | 0 | 0 | 0 | 0 | 0 | 1 | GSTD3__glutathione_S-transferase_(GSTD3) |
| AFUN003303 | 0 | 0 | 0 | 0 | 0 | 1 | GSTU3__glutathione_S-transferase_(GSTU3) |

**Tables S8:** List of SNPs significant between the permethrin resistant (R) and field dead mosquitoes (C) (R-C) in Cameroon using

unpaired t-test.

| **Transcripts** | **Type of substitution** | **position** | **Nucleotide** | **Amino acid** | **R-C** | **R-S** | **C-S** | **Description** |
| --- | --- | --- | --- | --- | --- | --- | --- | --- |
| AFUN015956 | NON_SYNONYMOUS | 1529 |  | I504V | 4.8 | 4.7 | 0.6 | CYP304B1_Cytochrome P450 |
| AFUN008618 | INTRONIC | 1141 | T>A |  | 4.4 | 4.6 | 0.3 | Chorion peroxidase |
| AFUN010472 | INTRONIC |  | A>G |  | 3.9 | 5.1 | 1.5 | mannosyltransferase |
| AFUN006951 | INTRONIC |  | A>C |  | 4.7 | 0.5 | 3.6 | transmembrane protease serine 9 |
| AFUN000721 | SYNONYMOUS | 1371 | T>C | F457F | 3.7 | 2.6 | 10.9 | peroxidasin |
| INTERGENIC | between AFUN007110 and AFUN007146 | | T>TGCA | | 3.9 | 1.1 | 1.2 |  |
|  | Intergenic |  |  |  | 3.1 | 2.2 | 0.4 |  |
| AFUN007162 | SYNONYMOUS | 5671 | C>T | I1581I | 3.9 | 11.7 | 5.7 | ATP-binding cassette sub-family A member 1-like |
| AFUN007162 | SPLICE_SITE |  | CAAAC |  | 3.1 | 2.9 | 11.8 | ATP-binding cassette sub-family A member 1-like |
| AFUN008257 | INTRONIC |  | T>A |  | 3.4 | 1.5 | 1.7 | carboxypeptidase A1-like |
| AFUN010230 | INTRONIC |  | T>A |  | 3.1 | 10.2 | 2.2 | suppressor of tumorigenicity 14 homolog |
| AFUN015111 | 3PRIME_UTR | 2062 | C>A |  | 3.8 | 5.0 | 0.8 | chymotrypsin-like protease CTRL-1 |
| AFUN015111 | 3PRIME_UTR | 1995-1996 | G>GT |  | 3.7 | 4.4 | 0.8 | chymotrypsin-like protease CTRL-1 |
| AFUN015111 | 3PRIME_UTR | 1995-1996 | GT>G |  | 3.5 | 4.3 | 1.2 | chymotrypsin-like protease CTRL-1 |
| AFUN015111 | NON_SYNONYMOUS | 1484 | A>G | D476G | 3.1 | 4.5 | 0.9 | chymotrypsin-like protease CTRL-1 |
| AFUN015808 | INTRONIC |  | T>G |  | 3.5 | 3.5 | nd | GSTE3_glutathione S-transferase |
| AFUN007217 | INTRONIC |  | C>T |  | 3.5 | 0.4 | 2.3 | molecular chaperone |
| AFUN011664 | SYNONYMOUS | 1510 | G>T | V478V | 3.1 | 0.4 | 3.1 | complement factor I |
| AFUN002623 | SYNONYMOUS | 426 | A>G | S142S | 3.1 | 0.8 | 1.1 | cytoplasmic type 5-like |
| AFUN005715 | SYNONYMOUS | 1375 | C>T | Y409Y | 3.1 | 0.8 | 3.6 | CYP315A1_Cytochrome P450 |
| AFUN010919 | SYNONYMOUS | 150 | T>C | I50I | 3.4 | 1.6 | 0.8 | CYP6M1c_cytochrome P450 |
| AFUN004767 | SYNONYMOUS | 396-397 |  |  | 3.8 | 2.2 | 0.4 | odr-4 homolog isoform X3 |
| AFUN002021 | SYNONYMOUS | 896 |  | S275S | 3.1 | 3.1 | nd | alpha-amylase |
| AFUN002796 | 3PRIME_UTR | 2827 |  | I193M | 3.6 | 1.2 | 3.4 | ATP-binding cassette sub-family G member 2-like |
| AFUN001673 | SYNONYMOUS | 1532 |  | R497R | 3.4 | 1.0 | 3.4 | ABC-2 type transport system ATP-binding |
| AFUN003058 | INTRONIC |  |  |  | 3.0 | 0.4 | 2.5 | peptidase M28 |
| AFUN009589 | SYNONYMOUS | 1026 |  | H342H | 3.9 | 0.3 | 3.2 | gastrula zinc finger -like isoform X1 |
| AFUN007527 | NON_SYNONYMOUS | 505 |  | V169L | 3.6 | 6.9 | 1.2 | phosphopantothenoylcysteine decarboxylase |
| AFUN007530 | SYNONYMOUS | 792 |  | A264A | 3.7 | 1.1 | 4.8 | chromosome partitioning |
| AFUN007530 | SYNONYMOUS | 789 |  | G263G | 3.1 | 1.4 | 4.7 | chromosome partitioning |

Nd, not determined

**Table S9:** List of the most significant SNPs between the permethrin resistant (R) mosquitoes in Cameroon and the susceptible lab strain FANG.

| **Nucleotide** | **Type substitution** | **Nucleotide** | **position** | **Amino acid** | **Description** | **Ref** | **Variant** |
| --- | --- | --- | --- | --- | --- | --- | --- |
| AFUN004689 | SYNONYMOUS_CODING | 1113-1113 | 371-371 | T->T | cuticular_protein_RR-2_family_(CPR130) | A | G |
| AFUN004766 | SYNONYMOUS_CODING | 564-564 | 188-188 | A->A | Serine protease 7 | C | G |
| AFUN015873 |  |  |  |  | zinc finger 345 | A | G |
| AFUN007471 | INTRONIC |  |  |  | R3H domain-containing 1 isoform X1 | G | C |
| AFUN008365 |  |  |  |  | Hexim | T | C |
| AFUN015980 |  |  |  |  | ATP-binding cassette sub-family G member 2-like [Scleropages formosus] | G | T |
| AFUN002876 | INTRONIC |  |  |  | Alpha-amylase 3 | C | T |
| AFUN014090 | INTRONIC |  |  |  | Leucine-rich repeat-containing 15 | G | A |
| AFUN008149 | SYNONYMOUS_CODING | 519-519 | 155-155 | V->V | Carboxypeptidase | T | C |
| AFUN008172 | SYNONYMOUS_CODING | 629-629 | 112-112 | L->L | Abl interactor 2 | C | A |
| AFUN009405 | INTRONIC |  |  |  | Scavenger receptor class B member 1 | A | AT |
| AFUN004002 | INTRONIC |  |  |  | argininosuccinate lyase | G | GT |
| AFUN004002 | INTRONIC |  |  |  | argininosuccinate lyase | C | A |
| AFUN000359 | INTRONIC |  |  |  | Chymotrypsin-2 | C | T |
| AFUN004689 | NON_SYNONYMOUS_CODING | 980-980 | 327-327 | G->A | cuticular_protein_RR-2_family_(CPR130) | C | G |
| AFUN004689 | NON_SYNONYMOUS_CODING | 802-802 | 268-268 | A->T | cuticular_protein_RR-2_family_(CPR130) | C | T |
| AFUN009738 | INTRONIC |  |  |  | OBP66_ANOGA ame: Full=General odorant-binding 66 Flags: Precursor | C | A |
| AFUN015723 | SPLICE_SITE | |  |  | CYP6AH1__cytochrome_P450_(CYP6AH1) | AGT | A |
| AFUN015760 | NON_SYNONYMOUS_CODING | 1315-1315 | 432-432 | K->N | UDP-glucuronosyltransferase 2B15 | C | G |
| AFUN007644 | SYNONYMOUS_CODING | 849-849 | 283-283 | I->I | Short-chain dehydrogenase | G | A |
| AFUN015786 | SYNONYMOUS_CODING | 605-605 | 181-181 | C->C | CYP6AA1__cytochrome_P450_(CYP6AA1) | G | A |
| AFUN008365 | 5PRIME_UTR | 302-302 |  |  | HEXIM | A | G |
| AFUN007471 | NON_SYNONYMOUS_CODING | 285-285 | 95-95 | Q->H | R3H domain-containing 1 isoform X1 | G | C |
| AFUN007471 | NON_SYNONYMOUS_CODING | 1167-1167 | 389-389 | M->I | R3H domain-containing 1 isoform X1 | G | A |
| AFUN007471 | SYNONYMOUS_CODING | 1443-1443 | 481-481 | V->V | R3H domain-containing 1 isoform X1 | G | A |
| AFUN007471 | SYNONYMOUS_CODING | 1650-1650 | 550-550 | Q->Q | R3H domain-containing 1 isoform X1 | G | A |
| AFUN004884 | SYNONYMOUS_CODING | 692-693 |  |  | CYP305A1__cytochrome_P450_(CYP305A1) | T | A |
| AFUN015908 | SYNONYMOUS_CODING | 1148-1148 | 351-351 | P->P | CYP15B1__cytochrome_P450_(CYP15B1) | G | A |
| AFUN005259 | 3PRIME_UTR | 2648-2648 |  |  | ABC__ATP-binding_cassette | G | T |
| AFUN015980 | SYNONYMOUS_CODING | 2451-2451 | 591-591 | D->D | ABC__ATP-binding_cassette | C | T |
| AFUN010918 | SYNONYMOUS_CODING | 978-978 | 317-317 | F->F | CYP6N1__cytochrome_P450_(CYP6N1) | C | T |
| AFUN015963 | NON_SYNONYMOUS_CODING | 679-680 | 207-207 | L->M | CYP6R1__cytochrome_P450_(CYP6R1) | C | A |
| AFUN004766 | SYNONYMOUS_CODING | 2277-2277 | 759-759 | H->H | Serine protease 7 | T | C |
| AFUN015873 | NON_SYNONYMOUS_CODING | 5149-5149 | 1227-1227 | M->T | zinc finger 345 | A | G |
| AFUN002085 | NON_SYNONYMOUS_CODING | 299-299 | 100-100 | D->G | C-type lectin | A | G |

**Tables S10:** List of SNPs significant between the permethrin resistant (R) and field dead mosquitoes (C) (R-C) in Uganda.

| **Transcripts** | **type of substitution** | **Nucleotide** | **Amino acid** | **Description** | **Reference** | **Variant Allele** | **R-C** | **R-S** | **C-S** |
| --- | --- | --- | --- | --- | --- | --- | --- | --- | --- |
| AFUN005585 | intronic |  |  | cytochrome P450 NADPH-cytochrome P450 reductase | ACC | A | 3.2 | 5.0 | 0.8 |
| AFUN015790 | non_synonymous | 904-904 | K262Q | CYP6AG1_Cytochrome P450 | T | G | 3.0 | 0.8 | 3.9 |
| AFUN002838 | 3prime_utr | 2721-2722 | | transcriptional enhancer factor TEF-1 | C | CAAAA | 4.3 | 0.3 | 2.2 |
| AFUN005389 | splice_site |  |  | General odorant-binding | A | C | 3.6 | 5.7 | 2.4 |
| AFUN005389 | intronic |  |  | General odorant-binding | AGA | AAT | 3.7 | 5.6 | 2.0 |
| AFUN005548 | synonymous | 2598-2598 | I818I | trafficking particle complex subunit 10 | A | C | 3.3 | nd | 3.3 |
| AFUN005548 | intronic |  |  | trafficking particle complex subunit 10 | A | G | 4.8 | 8.5 | 1.4 |
| AFUN005548 | intronic |  |  | trafficking particle complex subunit 10 | A | T | 4.7 | 8.3 | 1.4 |
| AFUN005548 | intronic |  |  | trafficking particle complex subunit 10 | A | T | 4.0 | 10.2 | 1.5 |
| AFUN005548 | intronic |  |  | trafficking particle complex subunit 10 | C | T | 4.3 | 10.9 | 1.4 |
| AFUN005548 | 3prime_utr | 3706-3707 | | trafficking particle complex subunit 10 | C | CT | 4.8 | 7.5 | 1.5 |
| AFUN001917 | synonymous | 1208-1208 | L113L | 3-methylcrotonyl- carboxylase alpha subunit | A | G | 3.5 | 2.0 | 0.9 |
| AFUN015866 | synonymous | 1139-1139 | F345F | CYP9J3__cytochrome_P450_(CYP9J3) | G | A | 3.8 | 3.8 | nd |
| AFUN003596 | synonymous | 1737-1737 | T579T | Xanthine dehydrogenase | C | G | 3.4 | 5.3 | 1.5 |
| AFUN015993 | 5prime_utr | 116-117 |  | CYP4D15__cytochrome_P450_(CYP4D15) | CCA | CCG | 4.1 | 0.8 | 4.5 |
| AFUN015993 | 5prime_utr | 116-116 |  | CYP4D15__cytochrome_P450_(CYP4D15) | A | G | 4.1 | 0.8 | 4.5 |
| AFUN004316 | intronic |  |  | CYP4H17_Cytochrome P450 | G | C | 3.2 | 3.2 | 0.3 |
| AFUN004352 | intronic |  |  | UDP-glucuronosyltransferase 2A3 | T | C | 3.0 | 0.3 | 2.3 |
| AFUN015957 | synonymous | 1256-1257 | | CYP304C1__cytochrome_P450_(CYP304C1) | A | G | 3.2 | 2.4 | 7.0 |
| AFUN007415 | intronic |  |  | 37 kDa salivary gland allergen Aed a 2 | A | T | 4.0 | 0.4 | 3.6 |
| AFUN007415 | non_synonymous | 1785-1785 | K579N | 37 kDa salivary gland allergen Aed a 2 | A | C | 3.8 | 7.3 | 9.7 |
| AFUN003078 | non_synonymous | 180-181 | H61Y | transmembrane protease serine 13-like | T | A | 3.3 | 4.3 | 0.7 |
| AFUN004380 | synonymous | 147-147 | R49R | Xanthine dehydrogenase | C | T | 3.0 | 3.0 | nd |
| AFUN000319 | non_synonymous | 115-115 | N18H | serine protease 14 | A | C | 3.1 | 0.6 | 1.6 |
| AFUN015759 | synonymous | 995-995 | Y326Y | UDP-glucuronosyltransferase 2B15 | A | G | 3.0 | 3.0 | 7.2 |
| AFUN007296 | synonymous | 520-520 | L102L | Elongation factor 1-gamma | C | T | 3.3 | 3.5 | 1.1 |
| AFUN002565 | synonymous | 1464-1464 | R488R | Transferrin | G | A | 3.6 | 2.2 | 5.0 |
| AFUN002565 | synonymous | 1458-1458 | F486F | Transferrin | G | A | 3.7 | 2.2 | 5.0 |
| AFUN005259 | synonymous | 726-726 | R46R | ABC__ATP-binding_cassette_ABCG4 | G | A | 3.1 | 0.5 | 3.1 |
| AFUN015745 | intronic |  |  | ABC__ATP-binding_cassette_ABCG4 | C | T | 3.1 | 12.4 | 3.6 |
| AFUN010118 | synonymous | 2347-2347 | G646G | solute carrier family facilitated glucose transporter member 9 | C | A | 3.0 | 3.2 | 0.3 |
| AFUN006647 | intronic | GCG |  | leucine-rich repeat-containing 4B-like | GCTA | GTA | 3.1 | 0.4 | 2.5 |
| AFUN006647 | intronic |  |  | leucine-rich repeat-containing 4B-like | A | G | 3.1 | 0.4 | 2.5 |
| AFUN002428 | synonymous | 1983-1983 | T661T | aminopeptidase Ey | A | T | 3.1 | 0.4 | 2.2 |
| AFUN002428 | synonymous | 952-952 | L318L | aminopeptidase Ey | A | G | 3.4 | 1.0 | 5.6 |
| AFUN005715 | intronic |  |  | CYP315A1__cytochrome_P450_(CYP315A1) | CGC | CAT | 5.5 | 1.1 | 8.0 |
| AFUN005721 | synonymous | 2358-2358 | N489N | Short-chain dehydrogenase reductase family 16C member 6 | C | T | 3.3 | 1.2 | 1.0 |
| AFUN005721 | 3prime_utr | 2810-2810 | | Short-chain dehydrogenase reductase family 16C member 6 | GT | G | 4.5 | 5.8 | 0.8 |
| AFUN015919 | synonymous | 142-142 | G28G | CYP6Z1__cytochrome_P450_(CYP6Z1) | A | G | 4.7 | 0.8 | 7.2 |
| AFUN002567 | synonymous | 3186-3186 | G1062G | Xanthine dehydrogenase 1 | C | A | 3.4 | 0.8 | 4.9 |
| AFUN002796 | synonymous | 1711-1711 | L480L | ABC__ATP-binding_cassette | C | T | 3.4 | 6.5 | 33.6 |
| AFUN002180 | intronic |  |  | ABC__ATP-binding_cassette | TTAG | T | 3.0 | 3.0 | nd |
| AFUN002180 | intronic |  |  | ABC__ATP-binding_cassette | C | T | 3.0 | 3.0 | nd |
| AFUN004493 | synonymous | 2083-2083 | T612T | zinc finger 345 | C | G | 3.1 | 3.4 | 1.1 |
| AFUN004951 | synonymous | 1728-1728 | D576D | Toll-like receptor 6 | A | G | 3.4 | 1.8 | 4.0 |
| AFUN004951 | synonymous | 1725-1725 | I575I | Toll-like receptor 6 | G | A | 3.1 | 1.7 | 4.6 |
| AFUN015883 | synonymous | 810-810 | V224V | serine protease 27 | A | G | 3.7 | 8.9 | 2.8 |
| AFUN015883 | synonymous | 789-789 | Y217Y | serine protease 27 | A | G | 3.4 | 1.4 | 1.0 |
| AFUN015884 | non_synonymous | 329-329 | T40K | chymotrypsin-like elastase | G | T | 3.0 | 2.0 | 5.8 |
| AFUN008096 | 3prime_utr | 6875-6875 | | toll-like receptor 13 | C | T | 3.1 | 6.6 | 1.5 |
| AFUN007480 | intronic |  |  | serine protease 56 | C | G | 3.4 | 2.9 | 0.5 |
| AFUN002058 | synonymous | 924-924 | P308P | UDP-glucuronosyltransferase 2B31 | C | T | 3.1 | 1.0 | 0.9 |
| AFUN002085 | non_synonymous | 188-188 | L63R | C-type lectin 37Da | T | G | 3.4 | 1.2 | 0.9 |
| Nd, not determined |  |  |  |  |  |  |  |  |  |

**Table S11:** List of the most significant SNPs between the permethrin resistant (R) mosquitoes in Uganda and the susceptible lab strain FANG.

| **Transcript** | **Type of substitution** | **Nucleotide** | **Amino Acid** | **position** | **Description** | **Ref** | **Variant** |
| --- | --- | --- | --- | --- | --- | --- | --- |
| AFUN005715 | INTRONIC |  |  | 24809 | CYP315A1 cytochrome_P450 | CGC | CAT |
|  |  |  |  |  |  |  |  |
|  |  |  |  |  |  |  |  |
| AFUN008410 | SYNONYMOUS | 1113-1113 | T371T | 660 | Serine protease mitochondrial | A | G |
| AFUN006967 | INTRONIC |  |  | 5942 | neuronal acetylcholine receptor subunit beta-2-like | G | A |
| AFUN004002 | SYNONYMOUS | 255-255 | D85D | 10341 | argininosuccinate lyase | A | G |
| AFUN004002 | SPLICE_SITE |  |  | 10325 | argininosuccinate lyase | C | T |
|  | INTERGENIC |  | between AFUN015793 and AFUN015792 | 15850 |  | G | A |
| AFUN015888 | NON_SYNONYMOUS | 78-78 | V14L | 16123 | CYP6P5__cytochrome_P450 | C | G |
|  |  |  |  |  |  |  |  |
| AFUN008348 | INTRONIC |  |  | 15427 | NPC2 homolog | A | G |
| AFUN015980 | SYNONYMOUS | 1278-1278 | A200A | 19631 | ABC__ATP-binding_cassette_ABCG4 | C | A |
| AFUN015980 | NON_SYNONYMOUS | 1650-1650 | Q324H | 19651 | ABC__ATP-binding_cassette_ABCG4 | A | T |
| AFUN005721 | SYNONYMOUS | 1176-1176 | V95V | 24902 | Short-chain dehydrogenase reductase family 16C member 6 | T | C |
| AFUN005721 | SYNONYMOUS | 1704-1704 | A271A | 24925 | Short-chain dehydrogenase reductase family 16C member 6 | A | G |
| AFUN015873 | NON_SYNONYMOUS | 5304-5304 | M1279V | 30530 | zinc finger 345 | T | C |
| AFUN008423 | INTRONIC |  |  | 31382 | chymotrypsin-like elastase | CAT | C |
|  |  |  |  |  |  |  |  |
| AFUN007549 | 3PRIME_UTR | 1842-1843 | | 10701 | CYP9K1__cytochrome_P450 | T | TCGGTTTCTTTCGTA |
| AFUN007549 | SYNONYMOUS | 1457-1457 | D426D | 10705 | CYP9K1__cytochrome_P450 | A | G |

**Tables S12:** List of SNPs significant between the permethrin resistant (R) and field dead mosquitoes (C) (R-C) in Malawi.

| **Transcripts** | **type of substitution** | **Nucleotide** | **Amino acid** | **Description** | **Reference** | **Variant Allele** | **R-C** | **R-S** | **C-S** |
| --- | --- | --- | --- | --- | --- | --- | --- | --- | --- |
| AFUN006796 | 5PRIME_UTR | 185-185 |  | Hsp70-Hsp90 organizing 3 | T | C | 3.4 | 0.8 | 3.9 |
| AFUN010384 | SYNONYMOUS | 450-450 | A150A | Venom dipeptidyl peptidase 4 | T | C | 3.5 | 1.8 | 1.3 |
| AFUN010384 | SYNONYMOUS | 402-402 | L134L | Venom dipeptidyl peptidase 4 | G | A | 3.6 | 1.8 | 1.1 |
| AFUN005498 | SYNONYMOUS | 1098-1098 | T366T | Ecdysteroid UDP-glucosyltransferase | A | C | 3.2 | 1.5 | 5.8 |
| AFUN007970 | SYNONYMOUS | 1593-1593 | T531T | Modular serine protease | G | C | 3.4 | 4.9 | 0.8 |
|  | INTERGENIC | between AFUN008401 and AFUN009482 | | | A | G | 3.0 | 5.0 | 1.4 |
| AFUN015865 | SYNONYMOUS | 1356-1356 | D419D | CYP9J4__cytochrome_P450_(CYP9J4) | A | G | 3.2 | 1.4 | 1.8 |
| AFUN015865 | SYNONYMOUS | 1086-1086 | V329V | CYP9J4__cytochrome_P450_(CYP9J4) | T | C | 3.0 | 0.8 | 3.9 |
| AFUN003690 | SPLICE_SITE |  |  | CYP4H14__cytochrome_P450_(CYP4H14) | C | T | 3.7 | 1.6 | 1.2 |
| AFUN003693 | INTRONIC |  |  | ABC__ATP-binding_cassette_ABCG1 | CT | C | 4.8 | 1.5 | 4.5 |
| AFUN000575 | SYNONYMOUS | 390-390 | S130S | UDP-glucuronosyltransferase 1-8 | C | T | 3.7 | 2.9 | 12.7 |
| AFUN000575 | SYNONYMOUS | 387-387 | Q129Q | UDP-glucuronosyltransferase 1-8 | T | C | 3.8 | 2.8 | 13.2 |
| AFUN000575 | INTRONIC |  |  | UDP-glucuronosyltransferase 1-8 | C | G | 3.2 | 6.7 | 1.1 |
| AFUN005978 | INTRONIC |  |  | Arrestin homolog | T | C | 3.7 | 2.8 | 0.4 |
| AFUN005978 | INTRONIC |  |  | Arrestin homolog | T | C | 3.1 | 2.9 | 0.8 |
| AFUN005978 | INTRONIC |  |  | Arrestin homolog | G | A | 3.1 | 1.4 | 1.2 |
| AFUN005978 | INTRONIC |  |  | Arrestin homolog | G | A | 3.1 | 1.5 | 1.1 |
| AFUN009937 | SYNONYMOUS | 242-242 | V48V | Cuticle Protein | G | A | 4.0 | 0.7 | 2.6 |
| AFUN009937 | SYNONYMOUS | 383-383 | Q95Q | Cuticle Protein | G | A | 3.8 | 0.8 | 2.5 |
|  | INTERGENIC |  |  |  | C | G | 3.0 | 5.6 | 2.3 |
| AFUN003956 | NON_SYNONYMOUS | 76-76 | S26T | Neuroligin | A | T | 4.9 | 0.9 | 5.7 |
| AFUN000373 | INTRONIC |  |  | Carboxylesterase | T | TCG | 3.3 | 0.7 | 1.6 |
| AFUN011499 | INTRONIC |  |  | Heat shock 70 | T | TTC | 3.0 | 1.5 | 7.4 |
| AFUN009783 | SYNONYMOUS | 351-351 | D117D | Troponin isoform 3 | G | A | 4.8 | 4.8 | 0.8 |
| AFUN010671 | INTRONIC |  |  | Melanization protease 1 | C | A | 3.0 | 4.9 | 1.1 |
| AFUN010472 | SYNONYMOUS | 849-849 | L256L | GPI mannosyltransferase 4 | T | C | 3.8 | 2.1 | 0.8 |
| AFUN010493 | INTRONIC |  |  | cuticular_protein_RR-2_family_(CPR113) | TA | T | 3.2 | 1.1 | 6.5 |
| AFUN010493 | INTRONIC |  |  | cuticular_protein_RR-2_family_(CPR113) | G | A | 3.5 | 6.0 | 3.7 |
| AFUN009068 | INTRONIC |  |  | Carboxylesterase | TTC | TTA | 3.7 | 4.4 | 0.8 |
| AFUN002942 | NON_SYNONYMOUS | 310-310 | V104M | Toll-like receptor Tollo ame | C | T | 3.5 | 4.9 | 0.7 |
| AFUN007110 | INTRONIC |  |  | Failed axon connections | C | T | 3.2 | 1.8 | 0.7 |
| AFUN007162 | SYNONYMOUS | 5386-5386 | Q1486Q | ABC__ATP-binding_cassette | C | T | 3.0 | 5.2 | 13.2 |
| AFUN007162 | SYNONYMOUS | 4969-4969 | N1347N | ABC__ATP-binding_cassette | A | G | 7.5 | 8.5 | 0.8 |
| AFUN007162 | INTRONIC |  |  | ABC__ATP-binding_cassette | C | T | 3.1 | 1.2 | 4.8 |
| AFUN005946 | NON_SYNONYMOUS | 160-160 | L51I | Alpha-amylase | C | A | 3.2 | 1.2 | 2.6 |
| AFUN005946 | SYNONYMOUS | 1026-1026 | A339A | Alpha-amylase | C | T | 3.0 | 0.3 | 1.5 |
| AFUN005260 | 5PRIME_UTR | 144-145 |  | ABC__ATP-binding_cassette | G | GA | 3.2 | nd | 2.6 |
| AFUN015978 | SYNONYMOUS | 1596-1596 | T512T | ABC__ATP-binding_cassette | A | G | 4.3 | 5.7 | 1.1 |
| AFUN015978 | SYNONYMOUS | 1326-1326 | L422L | ABC__ATP-binding_cassette | A | G | 3.6 | 7.1 | 1.9 |
| AFUN015982 | SYNONYMOUS | 1611-1611 | L423L | ABC__ATP-binding_cassette | G | C | 3.8 | 5.0 | 0.8 |
| AFUN005062 | INTRONIC |  |  | Trypsin-6 | C | A | 3.1 | 0.8 | 3.1 |
| AFUN010118 | INTRONIC |  |  | Solute carrier family facilitated glucose transporter member 3 | G | A | 3.1 | nd | 3.1 |
|  | INTERGENIC |  |  |  | T | G | 4.7 | 4.7 | nd |
| AFUN010270 | SYNONYMOUS | 634-634 | D194D | Retinol dehydrogenase 14 | C | T | 3.8 | 3.8 | 0.5 |
| AFUN010270 | SYNONYMOUS | 1003-1003 | K317K | Retinol dehydrogenase 14 | G | A | 3.8 | 3.8 | nd |
| AFUN010921 | SYNONYMOUS | 954-954 | S318S | CYP6M1b__cytochrome_P450_(CYP6M1b) | C | T | 3.2 | nd | 3.5 |
| AFUN010921 | SYNONYMOUS | 789-789 | D263D | CYP6M1b__cytochrome_P450_(CYP6M1b) | G | A | 3.2 | 5.3 | 2.1 |
| AFUN010921 | INTRONIC |  |  | CYP6M1b__cytochrome_P450_(CYP6M1b) | G | A | 3.1 | 2.2 | 0.6 |
| AFUN015961 | SYNONYMOUS | 692-692 | Y223Y | CYP6Y1__cytochrome_P450_(CYP6Y1) | C | T | 4.0 | 4.0 | nd |
| AFUN015961 | SYNONYMOUS | 824-824 | N267N | CYP6Y1__cytochrome_P450_(CYP6Y1) | C | T | 3.0 | 3.0 | nd |
| AFUN002567 | NON_SYNONYMOUS | 2395-2395 | Q799E | Xanthine dehydrogenase 1 | C | G | 5.8 | 4.7 | 0.5 |
| AFUN002567 | SYNONYMOUS | 2664-2664 | N888N | Xanthine dehydrogenase 1 | C | T | 5.4 | 4.3 | 0.6 |
| AFUN002567 | SYNONYMOUS | 3384-3384 | V1128V | Xanthine dehydrogenase 1 | G | C | 5.3 | 4.9 | 0.5 |
| AFUN002567 | SYNONYMOUS | 3450-3450 | V1150V | Xanthine dehydrogenase 1 | T | G | 5.6 | 5.2 | 0.5 |
| AFUN002567 | INTRONIC |  |  | Xanthine dehydrogenase 1 | A | G | 3.1 | 2.3 | 9.3 |
| AFUN002796 | SYNONYMOUS | 1707-1707 | Y478Y | ABC__ATP-binding_cassette_ABCG1 | C | T | 3.4 | 5.0 | 1.3 |
| AFUN006361 | NON_SYNONYMOUS | 359-359 | K120T | Leucine-rich repeat transmembrane neuronal 2 | A | C | 4.6 | 1.2 | 5.7 |
| AFUN008975 | INTRONIC |  |  | ABC__ATP-binding_cassette_ABCGN | GATGGATGA | G | 4.4 | 1.5 | 9.1 |
| AFUN015716 | NON_SYNONYMOUS | 2872-2872 | V942A | Alpha-2-macroglobulin domain-containing 7 | A | G | 3.1 | 8.2 | 1.7 |
| AFUN010202 | SYNONYMOUS | 180-180 | S60S | Glutactin | T | C | 4.3 | 0.5 | 3.2 |

**Table S13:** List of the most significant SNPs between the permethrin resistant (R) mosquitoes in Malawi and the susceptible lab strain FANG.

| **Nucleotide** | **Amino Acid** | **position** | **Description** | **Reference** | **Variant** |
| --- | --- | --- | --- | --- | --- |
| AFUN005498-RA |  | 1550 | Ecdysteroid UDP-glucosyltransferase | G | C |
| AFUN005498-RA |  | 1485 | Ecdysteroid UDP-glucosyltransferase | AGT | AAAACAA |
| AFUN006967-RA |  | 4446 | Acetylcholine receptor subunit alpha-like 1 | G | A |
| AFUN014090-RA |  | 4278 | Leucine-rich repeat-containing 15 | G | A |
|  |  |  |  |  |  |
| AFUN015745-RA | A543A | 14763 | ABC__ATP-binding_cassette_ABCG4 | C | T |
| AFUN015801-RA | A124A | 12642 | CYP6P2__cytochrome_P450_(CYP6P2) | T | C |
|  |  |  |  |  |  |
| AFUN002116-RA |  | 22805 | toll-like receptor 13 | C | A |
| AFUN008975-RA |  | 22546 | ABC__ATP-binding_cassette_ABCG4 | C | T |

**Table S14**: Genetic diversity parameters of the Coding sequences of the *CYP9K1* full sequence (2707bp) using SureSelect enrichment sequencing.

| **Sample** | **N** | **S** | **h** | | **Hd** | | **π** | **D** | **D*** | **Ka** | **ks** | **Ka/ks** |
| --- | --- | --- | --- | --- | --- | --- | --- | --- | --- | --- | --- | --- |
| **FUMOZ** | 20 | 12 | 5 | | 0,726 | | 0,00209 | 1.96309^ns^ | 1.09797^ns^ | 0.00113 | 0.00513 | 0.22027 |
| **FANG** | 20 | 38 | 7 | | 0,789 | | 0,00296 | -1.00186^ns^ | -1.30781^ns^ | 0.00263 | 0.00399 | 0.65914 |
| **Cameroon** | | | | | | | | | | | | |
| **Alive** | 20 | 101 | | 20 | | 1 | 0.00924 | -0.94105^ns^ | -1.14505^ns^ | 0.00733 | 0.01528 | 0.47971 |
| **Dead** | 20 | 84 | | 18 | | 0.989 | 0.00795 | -0.69920^ns^ | -0.43540^ns^ | 0.00684 | 0.01146 | 0.59685 |
| **All** | 40 | 123 | | 38 | | 0.997 | 0.00884 | -0.96698^ns^ | -1.34256^ns^ | 0.00708 | 0.01443 | 0.49064 |
| **Uganda** | | | | | | | | | | | | |
| **Alive** | 20 | 20 | 3 | | 0.195 | | 0.00077 | -2.39141** | -3.48452** | 0.00054 | 0.00152 | 0.35526 |
| **Dead** | 20 | 22 | 3 | | 0.195 | | 0.00081 | -2.47210 *** | -3.79631** | 0.00054 | 0.00169 | 0.31952 |
| **All** | 40 | 35 | 5 | | 0.192 | | 0.00079 | -2.58300*** | -4.23869** | 0.00053 | 0.00159 | 0.33333 |
| **Malawi** | | | | | | | | | | | | |
| **Alive** | 20 | 39 | 14 | | 0.947 | | 0.00582 | 1.72091^ns^ | 0.81532^ns^ | 0.00332 | 0.01109 | 0.29936 |
| **Dead** | 20 | 37 | 18 | | 0.989 | | 0.00519 | 1.37264^ns^ | 1.2256^ns^ | 0.00332 | 0.01109 | 0.29936 |
| **All** | 40 | 42 | 29 | | 0.974 | | 0.00561 | 1.90073^ns^ | 0.57839^ns^ | 0.00362 | 0.01195 | 0.030292 |
| **All samples from the three countries** | | | | | | | | | | | | |
| **All alive** | 60 | 120 | 37 | | 0.908 | | 0.00753 | -0.94236^ns^ | -2.10098^ns^ | 0.00570 | 0.01332 | 0.42792 |
| **All dead** | 60 | 95 | 39 | | 0.911 | | 0.00695 | -0.54323^ns^ | -0.61282^ns^ | 0.00535 | 0.01204 | 0.44435 |
| **Total all** | 120 | 137 | 72 | | 0.909 | | 0.00727 | -1.01919^ns^ | -2.6535^ns^ | 0.00550 | 0.01285 | 0.42801 |

*, P < 0.05; **, P < 0.01; ***, P < 0.001

**Table S15**: Genetic diversity parameters of the Coding sequences only of *CYP9K1* (1614bp) using SureSelect enrichment sequencing.

| **Sample** | **N** | **S** | **h** | **Hd** | **π** | **D** | **D*** | **Ka** | **ks** | **Ka/ks** |
| --- | --- | --- | --- | --- | --- | --- | --- | --- | --- | --- |
| **FUMOZ** | 20 | 12 | 5 | 0.726 | 0.00350 | 1.963^ns^ | 1.1^ns^ | 0.00036 | 0.0132 | 0.0273 |
| **FANG** | 20 | 35 | 6 | 0.747 | 0.00429 | -1.178^ns^ | -1.392^ns^ | 0.00008 | 0.01726 | 0.00465 |
| **Cameroon** | | | | | | | | | | |
| **Alive** | 20 | 93 | 20 | 1 | 0.01417 | -0.809^ns^ | -1.070^ns^ | 0.00064 | 0.05588 | 0.0115 |
| **Dead** | 20 | 73 | 18 | 0.989 | 0.01192 | -0.465^ns^ | -0.088^ns^ | 0.00067 | 0.04661 | 0.0144 |
| **All** | 40 | 107 | 38 | 0.997 | 0.01346 | -0.747^ns^ | -1.095^ns^ | 0.00065 | 0.05298 | 0.0123 |
| **Uganda** | | | | | | | | | | |
| **Alive** | 20 | 16 | 3 | 0.195 | 0.00105 | -2.324** | -3.311** | 0.00000 | 0.00428 | 0 |
| **Dead** | 20 | 20 | 3 | 0.195 | 0.00124 | -2.451*** | -3.751** | 0.00008 | 0.00481 | 0.0166 |
| **All** | 40 | 29 | 5 | 0.192 | 0.00113 | -2.512*** | -3.815** | 0.00004 | 0.00450 | 0.00889 |
| **Malawi** | | | | | | | | | | |
| **Alive** | 20 | 37 | 14 | 0.947 | 0.00963 | 1.947^ns^ | 1.071^ns^ | 0.00051 | 0.03777 | 0.0135 |
| **Dead** | 20 | 36 | 18 | 0.989 | 0.00863 | 1.479^ns^ | 1.367^ns^ | 0.00032 | 0.03425 | 0.00934 |
| **All** | 40 | 39 | 28 | 0.969 | 0.00932 | 2.249* | 1.066ns | 0.00042 | 0.03676 | 0.0114 |
| **All samples from the three countries** | | | | | | | | | | |
| **All alive** | 60 | 104 | 37 | 0.908 | 0.0120 | -1.772^ns^ | -1.609^ns^ | 0.00055 | 0.04751 | 0.0116 |
| **All dead** | 60 | 84 | 39 | 0.911 | 0.0111 | -0.091^ns^ | -0.147^ns^ | 0.00055 | 0.04365 | 0.0115 |
| **Total all** | 120 | 119 | 83 | 0.942 | 0.0117 | -2.49* | -1.91^ns^ | 0.00051 | 0.04614 | 0.0110 |

*, P < 0.05; **, P < 0.01; ***, P < 0.001

**Table S16**: Genetic diversity parameters of the noncoding sequences only of *CYP9K1* (introns and UTR; 1093bp) using SureSelect enrichment sequencing.

| **Sample** | **N** | **S** | **h** | **Hd** | **π** | **D** | **D*** | **Ka** | **ks** | **Ka/ks** |
| --- | --- | --- | --- | --- | --- | --- | --- | --- | --- | --- |
| **FUMOZ** | 20 | 00 | 1 | 0.00 | **0.0009** | 0.00ns | 0.00ns | 0.00 | 0.000 | 0.000 |
| **FANG** | 20 | 3 | 3 | 0.542 | **0.00383** | 0.75363ns | -0.12425ns | 0.00516 | 0.000 | / |
| **Cameroon** | | | | | | | | | | |
| **Alive** | 20 | 12 | 10 | 0.8 | **0.00196** | -1.66866ns | -1.38997ns | 0.00931 | 0.00264 | 3.52 |
| **Dead** | 20 | 11 | 10 | 0.837 | **0.00209** | -1.70118ns | -1.89302ns | 0.00825 | 0.00762 | 1.0826 |
| **All** | 40 | 16 | 18 | 0.817 | **0.00202** | -1.91248ns | -2.13794ns | 0.00869 | 0.00537 | 1.6182 |
| **Uganda** | | | | | | | | | | |
| **Alive** | 20 | 4 | 2 | 0.100 | 0.00141 | -1.86788ns | -2.62685ns | 0.000143 | 0.00139 |  |
| **Dead** | 20 | 2 | 3 | 0.195 | **0.00018** | -1.51284ns | -CV 2.05308ns | 0.00095 | 0.00 | / |
| **All** | 40 | 6 | 4 | 0.146 | **0.00027** | -2.10096ns | -3.75121ns | 0.00119 | 0.00070 |  |
| **Malawi** | | | | | | | | | | |
| **Alive** | 20 | 2 | 2 | 0.100 | **0.00037** | -1.51284ns | -2.05308ns | 0.00095 | 0.0000 | / |
| **Dead** | 20 | 1 | 2 | 0.100 | **0.00009** | -1.1639ns | -1.53959ns | 0.00048 | 0.000 | / |
| **All** | 40 | 3 | 3 | 0.099 | **0.00014** | -1.71621ns | -2.88194* | 0.00071 | 0.000 | / |
| **All samples from the three countries** | | | | | | | | | | |
| **All alive** | 60 | 16 | 12 | 0.388 | **0.00087** | -2.30966** | -2.98936** | 0.00403 | 0.00138 | 2.92028 |
| **All dead** | 60 | 11 | 11 | 0.438 | **0.00083** | -2.21642** | -2.53822* | 0.00337 | 0.00272 | 1.23897 |
| **Total all** | 120 | 20 | 21 | 0.412 | **0.00085** | -2.40497** | -3.35309** | 0.00369 | 0.00208 | 1.774038 |
